# A Novel Circular RNA circITGa9 Predominantly Generated in Human Heart Disease Induces Cardiac Remodeling and Fibrosis

**DOI:** 10.34133/research.0303

**Published:** 2024-02-06

**Authors:** Feiya Li, William W. Du, Xiangmin Li, Jindong Xu, Nan Wu, Faryal Mehwish Awan, Yang Yang, Fariborz Asghari Alashti, Sheng Wang, Burton B. Yang

**Affiliations:** ^1^ Sunnybrook Research Institute and Department of Laboratory Medicine and Pathobiology, University of Toronto, Toronto, ON, Canada.; ^2^ State Key Laboratory of Applied Microbiology Southern China, Guangdong Provincial Key Laboratory of Microbial Safety and Health, National Health Commission Science and Technology Innovation Platform for Nutrition and Safety of Microbial Food, Institute of Microbiology, Guangdong Academy of Sciences, Guangzhou 510070, China.; ^3^ Department of Anesthesiology, Guangdong Cardiovascular Institute, Guangdong Provincial People’s Hospital & Guangdong Academy of Medical Sciences, Guangzhou, Guangdong Province, China.; ^4^ Department of Medical Lab Technology, The University of Haripur, Haripur, Pakistan.; ^5^ Department of Anesthesiology, Beijing Anzhen Hospital, Capital Medical University, Beijing, China.; ^6^ Institute of Medical Sciences, University of Toronto, Toronto, ON, Canada.

## Abstract

Recent studies have highlighted the pivotal roles of circular RNAs (circRNAs) in cardiovascular diseases. Through high-throughput circRNA sequencing of both normal myocardial tissues and hypertrophic patients, we unveiled 32,034 previously undiscovered circRNAs with distinct cardiac expression patterns. Notably, circITGa9, a circRNA derived from integrin-α9, exhibited substantial up-regulation in cardiac hypertrophy patients. This elevation was validated across extensive sample pools from cardiac patients and donors. In vivo experiments revealed heightened cardiac fibrosis in mice subjected to transverse aortic constriction (TAC) after circITGa9 injection. We identified circITGa9 binding proteins through circRNA precipitation followed by liquid chromatography tandem-mass spectrometry. Furthermore, circRNA pull-down/precipitation assays demonstrated that increased circITGa9 expression facilitated binding with tropomyosin 3 (TPM3). Specific binding sites between circITGa9 and TPM3 were identified through computational algorithms and further validated by site-directed mutagenesis. We further showed that circITGa9 induced actin polymerization, characteristic of tissue fibrosis. Finally, we developed approaches that improved cardiac function and decreased fibrosis by delivering small interfering RNA targeting circITGa9 or blocking oligo inhibiting the interaction of circITGa9 and TPM3 into TAC mice, which is amenable for further preclinical and translational development. We conclude that elevated circITGa9 levels drive cardiac remodeling and fibrosis. By pinpointing circITGa9 as a therapeutic target, we open doors to innovative interventions for mitigating cardiac remodeling and fibrosis.

## Introduction

Circular RNAs (circRNAs) constitute a substantial category of single-stranded RNAs that are covalently linked. These circular structures are formed by the back splicing of linear transcripts, connecting a 5′ splice donor with an upstream 3′ splice acceptor. CircRNAs exhibit resistance to exonuclease degradation, rendering them more stable compared to linear RNAs. CircRNAs have been identified throughout the genome, with the majority originating from protein-coding genes [1,2]. Further, most circRNAs are produced by exons of genes, although their expression levels may not necessarily correlate to the levels of the host genes [3]. These exonic circRNAs are highly conserved across many species [4,5]. The conservation suggests biological functions of circRNAs. Indeed, it has been reported that circRNAs play important roles in development of cancer, cardiovascular, and neurological disorders [6–12], as circRNAs may regulate protein synthesis [13] and autophagy [14]. They can also regulate cell proliferation [15–17], migration [18], invasion [19], and apoptosis [20,21]. Many cardio-specific circRNAs have been demonstrated to play crucial roles in cardiac disorders, including cardiac hypertrophy, cardiomyopathy, remodeling, artery diseases, and myocardial infarction [22–25]. It has been shown that circRNAs are dynamically expressed in the different stages of development, showing specific spatial and temporal expression in mouse and human tissues [26–28]. These studies revealed many circRNAs are expressed in an organ-specific manner and can be enriched with tissue-specific biological functions.

While some circRNAs are expressed at much higher levels, most circRNAs are expressed at lower levels relative to their parental linear transcripts. Traditional RNA sequencing would only reveal the relatively highly expressed circRNAs. For low-abundance circRNAs, it is essential to remove the linear RNAs before sequencing to detect these low-abundance species. Although different organs have been subject to deep sequencing analysis of circRNA species, many disease-associated circRNAs have not been detected due to their low abundance in normal tissues [29,30]. These circRNAs may be up-regulated in disease state and play roles in disease progression. This study was designed to search for novel circRNAs in cardiac hypertrophy associated with fibrosis and explore their effects by deep circRNA sequencing and their functions in a mouse transverse aortic constriction (TAC) model.

## Results

### Increased circITGa9 levels in patients with cardiac hypertrophy

**Fig. 1.**
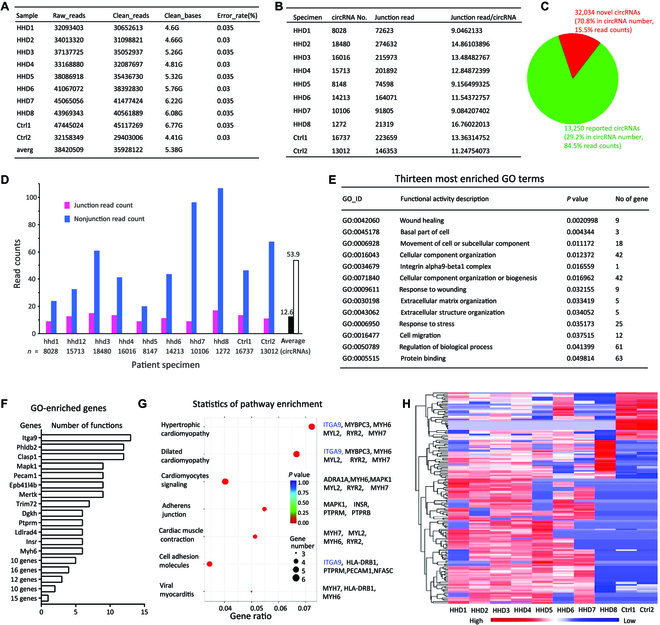
Expression of circRNAs in heart hypertrophy. (A) The total number of read counts per sample showing the deepness of sequencing. HHD, human heart disease; ctrl, control; averg, average. (B) The number of circRNAs read per sample and the average read counts of junction per circRNA. (C) The number of reported and unreported circRNAs detected in the sequencing. (D) The average junction read counts and nonjunction read counts per circRNA in all patient samples. (E) Analysis of the enriched GO revealed 13 functional activities involving 295 genes. (F) Only ITGa9 showed up in all 13 functional activities. (G) Analysis of all genes in the KEGG pathways revealed 15 genes were involved. ITGa9 showed up in 3 of them. (H) Heatmap of differentially expressed novel circRNA profiles in patients with cardiac hypertrophy relative to heart tissues without heart disease.

We hypothesized that there were differentially expressed circRNAs in human heart disease and collected samples from patients with different heart diseases that were diagnosed with pathogenic hypertrophy. The controls were samples from normal myocardial tissues without cardiac disease obtained from tissue donation. The samples were subjected to high-throughput circRNA sequencing. To maximize detection of low-abundance circRNAs, we removed most linear RNAs by ribonuclease (RNase) R treatment and only sequenced circRNAs. We thus reached average read counts of 38,420,509 per sample, having 35,928,122 clean read counts, in which all sequences could be matched to database (Fig. 1A). Each circRNA was identified by at least 2 read counts spanning a head-to-tail splice junction. All circRNAs identified showed similar length distribution in each sample with a total of 45,284 circRNAs identified (Supplementary Materials, Fig. S1A).

Among the identified circRNAs, 13,250 were already included in circBase [31], a number similar to early reported known circRNAs detected in human hearts [32], while the rest 32,034 have not been previously reported. The average read counts of each sample were much higher than the minimal 2 read count requirements (Fig. 1B). The normal myocardial tissues also produced large number of circRNAs, suggesting that many circRNAs were tissue-specific. We found that the reported circRNAs had a much higher abundance than the unreported circRNAs. Although they only represented 29.2% of the total circRNAs detected, they showed their abundance by taking 84.5% read counts, while 70.8% of the unreported circRNAs only had 15.5% read counts (Fig. 1C). Thus, the reported circRNAs were 13.2-fold higher in abundance relative to the unreported circRNAs. We took an additional approach to validate the sequencing results. Every read sequence was mapped to the circRNA junction (containing head-to-tail link) and nonjunction (the circRNA sequence but not the head-to-tail link) sequences. We found that every junction sequence read had 4.36 nonjunction sequences read (Fig. 1D). This is a reasonable proportion as most of the circRNAs are within 1,000 nucleotides. It also suggests that the sequencing procedure was successful and not contaminated by linear counterparts.

When we observed a large number of novel circRNAs that have not been previously detected, we reasoned that the diseased hearts displayed tissue-specific and function-specific circRNAs that might be responsible for disease development. We thus focused on this large group of circRNAs (Table S1). Due to their low abundance, the differences of most circRNAs between the diseased and normal hearts did not reach statistical significance. Compared to the normal myocardium, the numbers of differentially expressed circRNAs at a significant 2-fold cutoff were 152, in which 34 were down-regulated and 118 were up-regulated (Fig. S1B).

We then examined the genes that were enriched in classification of gene ontology (GO) system, since the circRNAs were generated from the pre-mRNAs of the genes. In GO classification, genes are grouped into sets of functional characteristics. In a total of 2,900 functional activities involving 11,243 genes, 13 functional activities reached significant levels (Fig. 1E). The number of genes involved in these 13 functional activities were 295. Among the 295 genes, only ENSG00000144668, the gene ID of integrin-α9, appeared in all the 13 functional characteristics (Fig. 1F).

We examined enrichment of the genes involvement in the generation of the differentially expressed circRNAs using Kyoto Encyclopedia of Genes and Genomes (KEGG) pathways that is a collection of databases dealing with biological pathways. In the 412 KEGG pathways, 7 KEGG pathways reached significant levels that involved 15 genes (Fig. 1G), many of which were cardiac-specific. ENSG00000144668 was involved in 3 pathways including hypertrophic cardiomyopathy, dilated cardiomyopathy, and cell adhesion molecules. These 3 pathways are important in cardiac remodeling.

Related to these enriched functional characteristics and KEGG pathways was the differential expression of the circRNAs. We thus examined the heatmap of the differentially expressed novel circRNAs and observed an increased number of red-labeled circRNAs due to up-regulation in the patients with heart disease relative to the normal cardiac tissues (Fig. 1H). We analyzed the highest and lowest differentially expressed circRNAs and the circRNAs with higher read counts since these would be the potential circRNAs modulating the development of pathogenic hypertrophy. In the short list, we selected 6 up-regulated and 8 down-regulated circRNAs (Table 1). We searched the sequence of hg38_circ_0030600 by using the junction sequences of the circRNAs obtained from high-throughput sequencing (Table S2) and found that the junction sequence was from exons 14 and 15 of integrin-α9.

**Table 1.** Differentially expressed circular RNAs

Circular RNA	Nucleotides	Gene	Hypertrophy	Normal	Change	*P* value
hg38_circ_0030600	NM_002207.2	ITGA9	1340.0477	33.86837	+3856.6%	0.000512978
hg38_circ_0028615	NM_024616.2	C3orf52	67.419388	0	+6641.9%	0.015818329
hg38_circ_0007593	NM_001297429.1	DGKH	59.102908	0	+5810.2%	0.027394571
hg38_circ_0025066	NM_001199145.1	XIRP2	44.178172	0	+4317.8%	0.016490499
hg38_circ_0035378	NM_000436.3	OXCT1	40.773402	0	+3977.3%	0.021321984
hg38_circ_0017433	NM_001079817.2	INSR	40.065325	0	+3906.5%	0.019665961
hg38_circ_0005451	NM_198827.4	ADGRD1	7.8647381	90.49402	−91.3%	0.01030171
hg38_circ_0039574	NM_015060.2	AVL9	5.2053736	53.09968	−90.1%	0.022319831
hg38_circ_0008333	NM_002471.3	MYH6	16.87635	155.9871	−89.1%	1.22927E-05
hg38_circ_0006743	NM_173591.3	OTOGL	9.3290469	81.30549	−88.5%	0.044658655
hg38_circ_0042258	NM_002380.4	MATN2	5.9855975	50.85606	−88.2%	0.000534851
hg38_circ_0034177	NM_133638.4	ADAMTS19	10.055074	62.18119	−83.8%	1.80489E-05
hg38_circ_0033095	NM_001278586.1	CORIN	103.98592	619.0351	−83.%	0.043161693
hg38_circ_0015928	NM_002035.2	KDSR	70.901769	373.4082	−81.0%	0.003828741

**Fig. 2.**
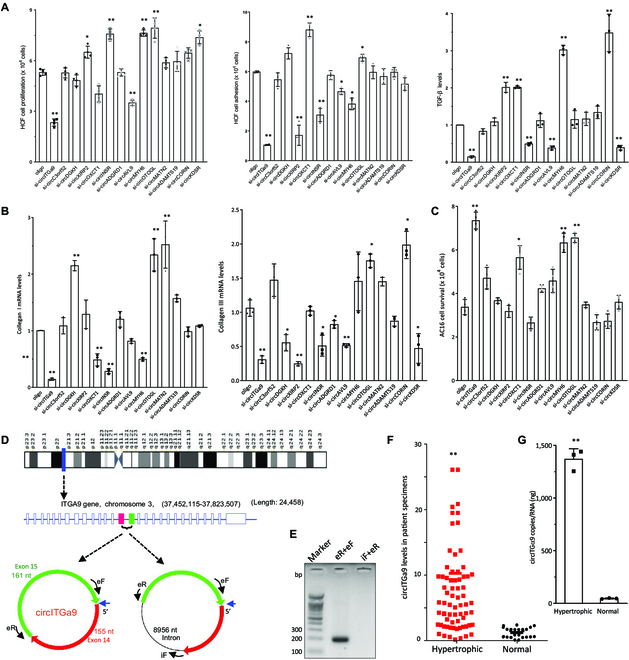
Effects of circITGa9 on potential fibrosis and heart hypertrophy. (A) HCF cells were transfected with siRNAs targeting the junction sequences of 14 circRNAs as indicated. Silencing circITGa9 decreased cell proliferation, adhesion, and TGF-β expression. *n* = 4. *P < 0.05, **P < 0.01 versus control oligo. (B) HCF cells were transfected with siRNAs targeting the junction sequences of 14 circRNAs as indicated. Silencing circITGa9 decreased collagen expression. *n* = 4. *P < 0.05, **P < 0.01 versus control oligo. (C) AC16 cells were transfected with siRNAs as indicated. Silencing circITGa9 increased cell Survival. *n* = 4. *P < 0.05, **P < 0.01 versus control oligo. (D) Structures of Chromosome 3, ITGa9 pre-mRNA, and potential structures of circITGa9. (E) RT-PCR showed revealed no intron was included in circITGa9. (F) Real-time PCR in hypertrophic patient hearts (*n* = 73) and normal heart donations (*n* = 25) showed that heart patients produced high levels of circITGa9. **P < 0.01 versus normal. (G) Digital droplet PCR showed that patient hearts with hypertrophy had higher copy numbers of circITGa9 compared to normal hearts. *n* = 3. **P < 0.01 versus normal.

To examine whether the listed circRNAs played roles in cardiac remodeling leading to hypertrophy and fibrosis, we designed small interfering RNAs (siRNAs) targeting the junction sequence of each of the highest and lowest differentially expressed circRNAs with higher read counts (14 in total) and tested their effects. Each siRNA and the control oligo were delivered into the commercialized human cardiac fibroblast (HCF) cell line. We confirmed that the siRNAs were able to down-regulate the related mRNAs (Fig. S2A). In the 14 siRNAs tested, silencing different circRNAs had a regulatory role in different cell functions, but only silencing hg38_circ_0030600 (circITGa9) showed the most significant change in fibrosis related hallmarks, including decrease of fibroblast adhesion, proliferation, transforming growth factor-β (TGF-β) expression (Fig. 2A), and collagen deposition (Fig. 2B) but increase of cardiomyocyte viability (Fig. 2C). Treatment of HCF cells with TGF-β significantly increased circITGa9 expression (Fig. S2B). Our analyses of GO enrichment, KEGG enrichment, heatmap, and functional assays all pointed to the importance of circRNAs produced by integrin-α9 that has been reported to produce 10 circRNAs [3,31]. However, none of these reported circRNAs was expressed at a significant level in cardiac tissues (Table 2). Interestingly, however, one of the 2 unreported circRNAs that were newly detected in our cardiac-specific sequencing, hg38_circ_0030600, was the most up-regulated circRNA with high read counts.

**Table 2.** Expression of circITGa9 in heart tissues

circRNA	Exons	hhd1	hhd2	hhd3	hhd4	hhd5	hhd6	hhd7	hhd8	ctrl1	ctrl2
hsa_circ_0064840	2–4	0	11	70	60	0	43	22	0	0	0
hsa_circ_0064841	2–5	0	0	0	15	0	0	0	0	0	0
hsa_circ_0064842	2–14	0	0	0	0	0	0	0	0	0	0
hsa_circ_0123707	4–13	0	0	0	0	0	0	0	0	0	0
hsa_circ_0123708	6–15	0	0	0	0	0	0	0	0	0	0
hsa_circ_0123709	12–14	0	0	0	0	0	0	0	0	0	0
hsa_circ_0123711	19–26	0	0	0	0	0	0	0	0	0	0
hsa_circ_0123713	25–26	0	0	0	0	0	0	0	0	0	0
hsa_circ_0123714	27–28	0	0	0	0	0	0	0	0	0	0
hsa_circ_0123715	27–28	0	0	0	0	0	0	0	0	0	0
hg38_circ_0030600	14–15	1,436	1,013	1,453	1,564	1,676	394	788	2,397	40	27
hg38_circ_0030598	12–14	124	81	201	170	80	37	164	0	18	27

Note: hsa_circ_0123714 and hsa_circ_0123715 end in the mid of Exon 28.

Since circITGa9 was generated from exons 14 and 15 of integrin-α9, there was an intron of 8,945 base pairs between the exons. To assay whether this circRNA contained the intron or not, we designed specific pairs of primers to amplify exons 14 and 15 (Fig. 2D). Reverse transcription polymerase chain reaction (RT-PCR) analysis followed by gel electrophoresis revealed that there was no intron involved in circITGa9 (Fig. 2E). The PCR product was Sanger-sequenced to confirm the presence of the circITGa9 junction sequence (Fig. S2C).

We then examined clinical involvement of circITGa9. We designed 2 pairs of primers specifically amplifying the junction sequence of circITGa9 but not linear Itga9 mRNA. We extensively collected human cardiac specimens and selected 73 patient samples with cardiac hypertrophy and 25 normal heart samples from organ donations that had undetectable cardiac disease and performed real-time PCR. The levels of circITGa9 were significantly higher in the patient specimens relative to the donor samples (Fig. 2F). Digital droplet PCR measurements revealed significantly higher copy number of circITGa9 in the patient specimens compared to the donor samples (Fig. 2G).

### Effect of circITGa9 on heart functions

**Fig. 3.**
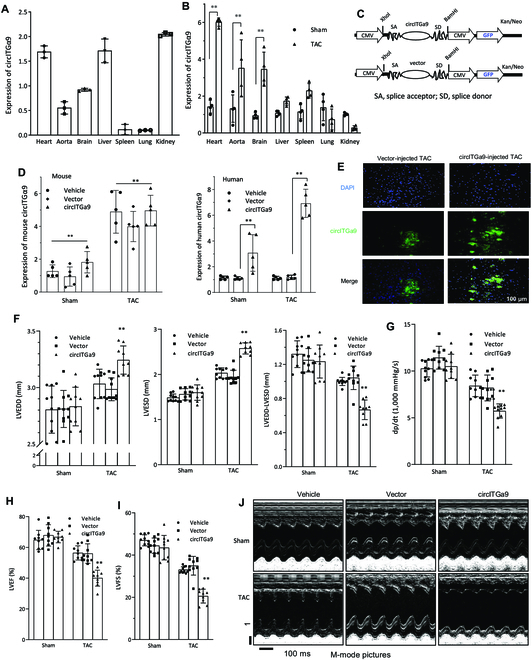
Overexpression of circITGa9 decreased heart function. (A) RNAs isolated from different tissues of mice were subject to real-time PCR measuring levels of circITGa9. (B) RNAs isolated from different tissues of mice with or without TAC were subject to real-time PCR measuring levels of circITGa9. TAC increased circITGa9 levels in the tissues of heart, aorta, and brain. **P < 0.01 versus sham. (C) Structure of circITGa9 expression construct. (D) Levels of human (left) and mouse (right) circITGa9 were measured by real-time PCR, showing the specificity of the primers we used. *n* = 5. **P < 0.01 versus vector control. (E) Representative photographs of FISH staining to confirm the successful circITGa9 plasmid delivery into the mouse hearts. (F) Echocardiography analysis showed that TAC increased LVESD and the LVEDD relative to the sham mice and the vector control. *n* = 10. **P < 0.01 versus vector control. (G to I) TAC reduced left ventricular pressure (dp/dt, G), LVEF (H) and LVFS (I) compared to the sham mice. Delivery of circITGa9 expression plasmids further decreased TAC effects significantly. *n* = 10. **P < 0.01 versus vector control. (J) Representative photographs of echocardiography from sham or TAC mice delivered with a vector or circITGa9 plasmid.

To test the involvement of circITGa9 in hypertrophic development, we generated pressure overload (PO) in mice by TAC. Normally, heart, liver, and kidney were the organs that expressed higher levels of endogenous circITGa9 compared with others (Fig. 3A). After TAC, the levels of endogenous circITGa9 were found increased the most in the heart tissues, followed by aorta and brain tissues (Fig. 3B).

To examine the role of circITGa9 in cardiac functions, we generated a human circITGa9 expression construct (Fig. 3C). Confirmation of circularization was performed by RT-PCR and DNA sequencing of the total RNA isolated from the cells transfected with circITGa9 or the vector control (Fig. S2D). The sequence of the construct is provided in Supplementary Materials.

Since circITGa9 is highly conserved between human and mouse (82.2% homology), we examined the functions of human circITGa9 in mice by delivering circITGa9 into the PO mice via TAC surgery followed by injecting nanoparticle-conjugated human circITGa9 expression plasmids into the mice peritoneally. As expected, we detected a significant increase of endogenous mouse circITGa9 in the heart 8 weeks after surgery (Fig. 3D, left). While ectopic delivery of human circITGa9 successfully increased circITGa9 levels in sham and TAC mouse hearts, it superimposed human circITGa9 levels in the TAC mice (Fig. 3D, right). In situ hybridization using a fluorescent probe confirmed the successful delivery of circITGa9 into mouse heart tissues (Fig. 3E). However, the levels of full-length linear ITGa9 mRNA were unaffected (Fig. S2E), suggesting specificity of the circRNA in cardiac remodeling.

We then performed heart functional measurements using the Vevo 2100 imaging system. We observed a significant increase in left ventricular end-systolic diameter (LVESD) and left ventricular end-diastolic diameter (LVEDD) in TAC mice by circITGa9 delivery ectopically (Fig. 3F). Subtraction of LVEDD by LVESD displayed a significant decrease by circITGa9 delivery. We also observed a significant decrease in left ventricular pressure (dp/dt, Fig. 3G), left ventricular ejection fraction (LVEF; Fig. 3H), and left ventricular fractional shortening (LVFS; Fig. 3I). M-mode pictures showed a decreased ventricular contraction in the TAC heart injected with circITGa9 expression plasmids (Fig. 3J). Taken together, the above results suggest a decreased heart function in the circITGa9 overexpression group compared to vector control group.

**Fig. 4.**
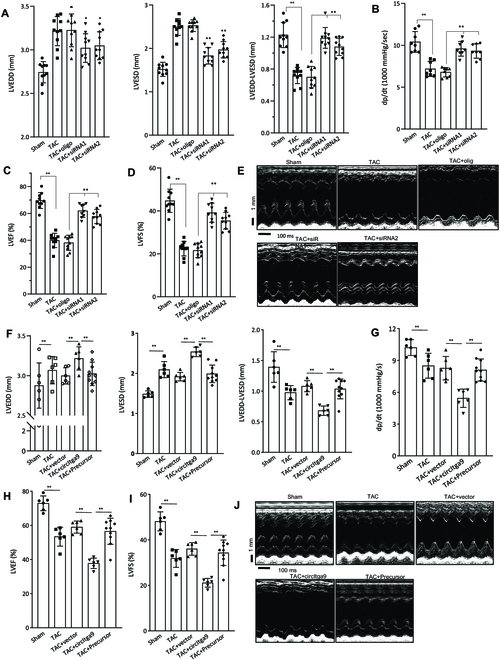
Confirmation of circITGa9 in decreased heart function. (A) Echocardiography analysis showed that TAC mice injected with circITGa9 siRNAs displayed decreased LVESD and LVEDD relative to the TAC mice treated with or without oligo injection. *n* = 10. *P < 0.05; **P < 0.01. (B to D) Delivering circITGa9 siRNAs increased dp/dt (B), LVEF (C), and LVFS (D) in TAC mice relative to oligo control. *n* = 10. **P < 0.01. (E) Representative photographs of echocardiography in TAC mice injected with or without circITGa9 siRNAs. (F) Delivery of the uncircularized circITGa9 precursor displayed decreased LVESD and LVEDD relative to the TAC mice injected with circITGa9. *n* = 6. **P < 0.01. (G to I) Delivering circITGa9 precursor increased dp/dt (G), LVEF (H), and LVFS (I) in TAC mice relative to mice injected with circITGa9. *n* = 6. **P < 0.01. (J) Representative photographs of echocardiography in TAC mice injected with circITGa9 and circITGa9 precursor.

We then designed 2 siRNAs targeting the junction sequence of circITGa9. The siRNAs were conjugated with gold nanoparticles and delivered into TAC mice. While the levels of circITGa9 were significantly increased in the heart 8 weeks after surgery, delivery of the siRNAs decreased circITGa9 levels (Fig. S2F). In heart functional measurements of TAC mice subjected to circITGa9 silencing, we detected a significant decrease in LVESD and LVEDD, but subtraction of LVEDD by LVESD showed significant increase (Fig. 4A). We also found a significant increase in left ventricular pressure (dp/dt, Fig. 4B), LVEF (Fig. 4C), and LVFS (Fig. 4D). M-mode pictures showed silencing circITGa9 could prevent left ventricular contraction reduction induced by TAC (Fig. 4E).

To further test the essential roles of the circularized circITGa9, we generated a construct that lacked the circularization signal and expressed as a linear counterpart (all construct and probe sequences provided in Table S3, primer sequences in Table S4, and siRNA sequences in Table S5). The Precursor was conjugated with gold nanoparticles and delivered into TAC mice. In heart functional assays, we detected a significant decrease in LVESD and LVEDD, but subtraction of LVEDD by LVESD showed significant increase in the Precursor group compared to the circITGa9 group (Fig. 4F). We also found a significant increase in dp/dt (Fig. 4G), LVEF (Fig. 4H), and LVFS (Fig. 4I). M-mode pictures showed improved heart function related to the circITGa9 group (Fig. 4J).

### Effect of circITGa9 on cardiac fibrosis

**Fig. 5.**
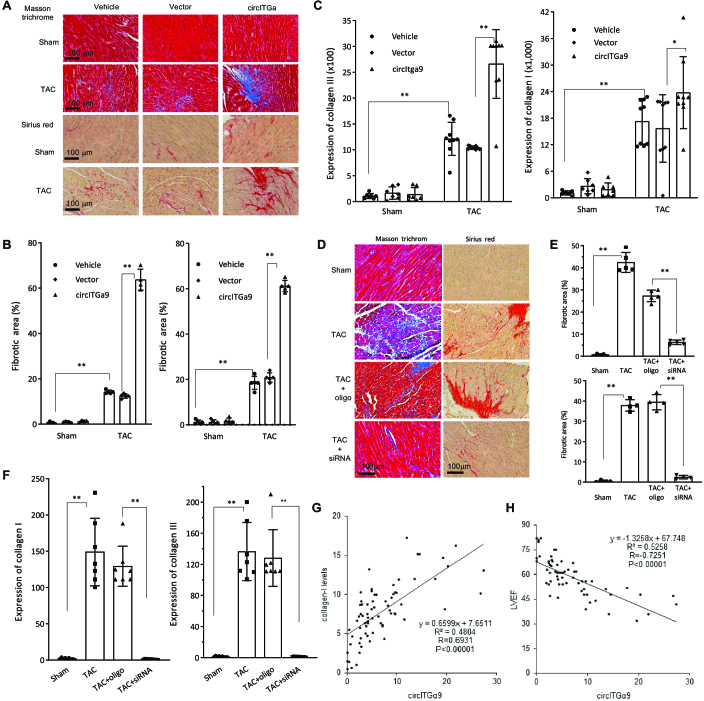
The effects of circITGa9 on fibrosis in pressure-overload hearts. (A) Representative photographs of Masson trichrome and Sirius red staining, showing increased staining in the TAC mouse hearts. The staining was further intensified by ectopic circITGa9 delivery. (B) Quantitation of Masson trichrome (left) and Sirius red (right) staining. *n* = 5. **P < 0.01 versus vector control. (C) Expression of collagen I (left) and collagen III (right) in mice heart tissues of TAC+sham, TAC+vector and TAC+circITGa9 mice. *n* = 9. *P < 0.05, **P < 0.01 versus vector control. (D) Representative photographs of Masson trichrome and Sirius red staining from mice treated with sham, TAC, TAC+oligo, and TAC+circITGa9 siRNAs. (E) Quantitation of Masson trichrome (left) and Sirius red (right) staining, showing delivering circITGa9 siRNA decreased both staining in TAC mice relative to oligo control. *n* = 5. **P < 0.01. (F) Expression of collagen I (left) and collagen III (right). Delivering circITGa9 siRNA decreased collagen expression in TAC mice relative to oligo control. *n* = 7. **P < 0.01. (G) Positive correlation between circITGa9 and collagen levels was revealed by real-time PCR in the patient specimens of hypertrophic hearts (*n* = 73). Pearson correlation was performed to evaluate the association. Trend line equation, *R*^2^, *R*, and *P* value were labeled in the figure. (H) Inverse correlation between circITGa9 levels and heart function LVEF was observed in the patients with hypertrophy (*n* = 62). Pearson correlation was performed to evaluate the association. Trend line equation, *R*^2^, *R*, and *P* value were labeled in the figure.

Since cardiac fibrosis is one of the major outcomes that closely associated with cardiac hypertrophic remodeling, we examined the role of circITGa9 in cardiac fibrosis by staining the heart tissues with Masson trichrome and Sirius red to visualize the collagen deposition (Fig. 5A). Quantification analysis showed that the staining of Masson trichrome and Sirius red were significantly increased in TAC mice hearts compared with sham control mice, while overexpression of circITGa9 through injection delivery further increased the collagen deposition in TAC mice (Fig. 5B). Furthermore, we analyzed expression of collagen I and collagen III in the heart tissues by real-time PCR and found that collagen expression was significantly up-regulated in TAC mice and delivering circITGa9 further increased collagen levels (Fig. 5C), resulting in enhanced fibrosis. In the heart tissues in which circITGa9 was silenced, Masson trichrome and Sirius red staining showed decreased levels of collagen deposition (Fig. 5D). Quantitation showed that the levels of Masson trichrome and Sirius red were significantly decreased (Fig. 5E). Additionally, real-time PCR revealed a significant reduction in collagen I and collagen III in these heart tissues (Fig. 5F). In the patient specimens, collagen I levels were positively correlated to circITGa9 expression (Fig. 5G), whereas circITGa9 expression was negatively correlated to cardiac function (Fig. 5H).

To further examine how circITGa9 regulated heart function, we analyzed the location of the delivered circRNA. Colocalization of circITGa9 with cardiac fibroblasts upon circITGa9 plasmid delivery was observed by fluorescence in situ hybridization (FISH) and immunofluorescence staining (Fig. S3A). Since fibroblasts play the most important roles in mediating the development of cardiac fibrosis and we observed the colocalization of circITGa9 and fibroblast, we examined the role of circITGa9 in primary isolated cardiac fibroblast cells. Overexpression of circITGa9 resulted in an increase in adhesion (Fig. S3B) and migration (Fig. S3C and D). Upon delivery of circITGa9, morphology change was observed where cardiac fibroblast became enlarged spread out and flat (Fig. S3E). Such morphological change implicates myofibroblast activation, a hallmark following fibrosis initiation.

### circITGa9 functions through binding with tropomyosin 3 and regulates actin polymerization

**Fig. 6.**
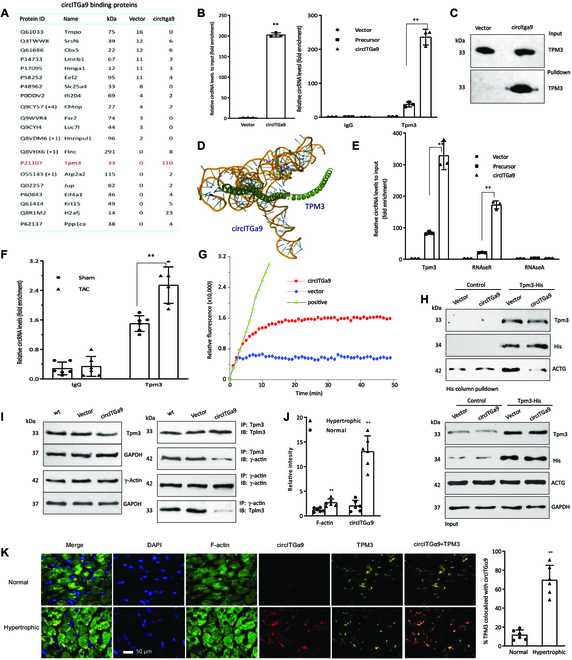
The binding of circITGa9 with TPM3. (A) Mass spectrometry assay showing the proteins pulled down by circITGa9. The numbers are peptides detected by the system. (B) Left, input levels of circITGa9 showing successful transfection of circITGa9 in MCF cells. Right, antibodies against TPM3 could precipitate circITGa9. *n* = 3. **P < 0.01 versus vector control. (C) TPM3 pulled down by the circITGa9 probe was confirmed on Western blotting detected by anti-TPM3 antibody. (D) The binding sites of circITGa9 with TPM3 protein was identified via computational approach. (E) The binding of TPM3 with circITGa9 in MCF cells upon circITGa9 overexpression with RNase A or RNase R treatment. RNase A, but not RNase R, abolished circITGa9 precipitated by antibodies against TPM3. *n* = 3. ***P < 0.001 versus vector control. (F) Antibodies against TPM3 precipitated significantly higher levels of circItga9 in mouse heart tissues subjected to TAC relative to sham. *n* = 6. **P < 0.01 versus sham. (G) Actin polymerization rates in MCF cells transfected with vector control or circITGa9 plasmids. Expression of circITGa9 increased actin polymerization. (H) MCF cells were cotransfected with circITGa9 and a His-tagged TPM3 expression construct (Tpm3-His). The cells cotransfected with TPM3-His and a control vector served as controls. Cell lysates were subjected to pulldown assay by a His-column, followed by Western blotting, probed with antibodies against Tpm3, His-tag, and actin (left). The lysates were also subjected to Western blotting to confirm equal amounts of input, probed with antibodies as indicated (right). In the presence of circITGa9, Tpm3-His precipitated decreased level of actin. (I) HEK 293T cells were transfected with circITGa9 and subjected to immunoprecipitation with antibodies against TPM3 and ϒ-actin followed by Western blotting. Transfection with circITGa9 did not affect TPM3 and ϒ-actin expression. While TPM3 coprecipitated with ϒ-actin, decreased levels of ϒ-actin was coprecipitated with TPM3 in the presence of circITGa9. (J) ImageJ analysis of in situ hybridization and immunofluorescence staining showed that hypertrophic heart tissues expressed higher levels of circITGa9 and F-actin (left). *n* = 6. **P < 0.01 versus normal. (K) Left: Typical in situ hybridization and immunofluorescence staining showed expression of circITGa9 (red), TPM3 (yellow), F-actin (green), and DAPI (blue) in hypertrophic hearts. Right: A higher percentage of TPM3 was detected to colocalized circITGa9 in hypertrophic heart samples. *n* = 6. **P < 0.01.

To uncover molecules that might be involved in mediating the function of circITGa9 in cardiac fibrosis, we performed coimmuno-precipitation assay by using a probe that targeted the junction sequence of circITGa9 in mouse cardiac fibroblast (MCF) cells. The proteins precipitated by the probe were identified by mass spectrometry. Proteins pulled down by the circITGa9 probe were identified, among which tropomyosin 3 (TPM3) ranked the top count (Fig. 6A). To confirm the mass spectrometry results and test the specificity of the interaction, we transiently transfected MCF cells with circITGa9 expression construct and the control vector. While the endogenous levels of circITGa9 were undetectable, transfection with circITGa9 significantly up-regulated circITGa9 levels (Fig. 6B left). We also used anti-TPM3 antibody to precipitate circITGa9 in MCF cells transfected with the vector, the uncircularized circITGa9 Precursor, and the circITGa9 constructs. Comparing with the Precursor, anti-TPM3 antibody precipitated significantly more circITGa9 in the ectopic expression of circITGa9 group, while there was no difference between the groups when using IgG antibody for precipitation, confirming specificity of the interaction between circITGa9 and TPM3 (Fig. 6B right). On the other hand, we used the circITGa9 probe to pull down circITGa9 and the circITGa9-binding proteins. The pull-down proteins were analyzed on Western blot probing with anti-TPM3 antibody to confirm the interaction of circITGa9 with TPM3 (Fig. 6C).

Utilizing a computational approach, we tentatively mapped the contact sites between circITGa9 and TPM3. Our analysis involved several steps, including computer-aided RNA structure modeling for circITGa9 using the minimum free energy algorithm and a machine translation system. Subsequently, we performed energy minimization using the YASARA Energy Minimization Server (see Fig. S4A). We then analyzed the best-predicted secondary structure of circITGa9 to understand its thermodynamic properties. As crystallographic data for TPM3 was not available, we employed a homology-based approach to generate its 3-dimensional target structure. To do this, we used the Phyre2 web portal v2.0 and the sequence of 1C1G as a template for modeling, following an “intensive” approach. The Phyre2 web portal yielded a model structure with 100% coverage and greater than 90% confidence, showing an 89% identity to 1C1G. This modeling confidence indicated that our model should accurately represent both the 2D and 3D structures of TPM3 (as depicted in Fig. S4B). To further assess the stereochemical quality of our Phyre2-predicted models, we performed a Psi/Phi Ramachandran plot analysis using the PROCHECK interactive server, as shown in Fig. S4C. The results indicated that 99.3% of the residues were within the favored region, 0.4% in the additional allowed region, 0.0% in generously allowed regions, and only 0.4% in the outlier region, demonstrating the high quality of our predicted model. Molecular simulations supported the notion that circITGa9 can efficiently interact with TPM3 (see Fig. S5A), revealing a minimal binding region for circITGa9 (see Fig. 6D). These interactions were further evaluated through contact maps and residue-level resolution contact maps of the circITGa9-TPM3 complex (see Fig. S5B and C). Detailed information on these interactions can be found in Tables S6 to S8.

To confirm the interaction of TPM3 with circITGa9 rather than linear ITGa9, the reaction mixture was treated with RNase A or RNase R after the immunoprecipitation. It showed that RNase A (digesting all RNAs) treatment abolished anti-TPM3 antibody precipitating circITGa9, but treatment with RNase R (digesting linear RNAs) did not (Fig. 6E). These results demonstrate that TPM3 bound to circITGa9 is resistant to RNase R digestion. In the collected mouse heart tissues, we also confirmed that antibody against TPM3 could precipitate significantly higher levels of endogenous circITGa9 relative to the control (Fig. 6F).

Tropomyosins play critical roles in stabilizing actin filaments, and the conformational change between tropomyosins and actins results in different states of polymerization of actin. Actin polymerization is important in regulating cell activities [33] and has been reported to be closely related to tissue fibrosis [34]. Therefore, we tested whether the binding of circITGa9 and TPM3 affects cardiac fibrosis through regulating actin polymerization. Total contents of actin were isolated from the cells by ultracentrifugation for actin polymerization assay. We obtained increased actin polymerization in the samples isolated from the circITGa9-transfected MCFs (Fig. 6G). Next, we tested how the binding of circITGa9 and Tpm3 increased actin polymerization. MCF cells were cotransfected with circITGa9 and a His-tagged TPM3 expression construct. Cell lysates were subjected to pulldown assay by a His column, followed by Western blotting, probed with antibodies against TPM3, His-tag, and γ-actin. In the presence of circITGa9, TPM3 precipitated decreased level of actin (Fig. 6H and Fig. S5D).

To further elucidate the mechanisms associated with the functions of circITGa9, we transfected HEK-293T cells with circITGa9 and performed coimmunoprecipitation assay using antibodies against TPM3 and actin. We found that expression of circITGa9 inhibited the interaction between TPM3 and actin (Fig. 6I). In the in situ hybridization and immunofluorescence staining of human heart tissues, we observed increased levels of circITGa9 and F-actin (Fig. 6J) and colocalization of circITGa9-TPM3 (Fig. 6K) in the hypertrophic heart tissues compared to normal heart tissues.

**Fig. 7.**
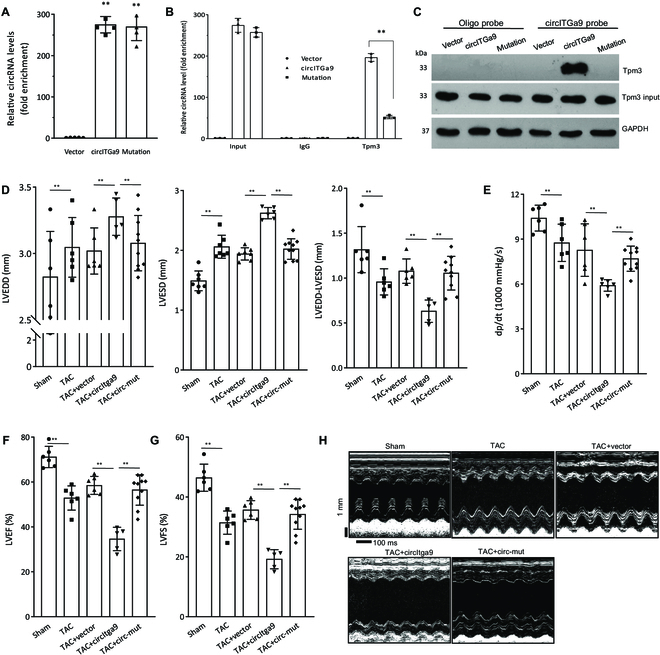
Mutation of the TPM3 binding abolished the cardiac functions of circITGa9. (A) Mutation on the binding site of circITGa9/TPM3 did not affect circularization of the transcript since real-time PCR showed equal amount of circRNAs. *n* = 4. ***P < 0.001 versus vector control. (B) The mutation significantly decreased anti-TPMs antibody precipitating the mutant circITGa9 relative to the normal circITGa9. *n* = 3. ***P < 0.001 versus vector control. (C) TPM3 was pulled down by the circITGa9 probe in MCF cells transfected with circITGa9 and the mutation constructs. Transfection with circITGa9 increased the probe pulling down TPM3. Mutation on the binding site of circITGa9/TPM3 abolished the circITGa9 probe pulling down TPM3 protein. (D) Delivery of the mutated circITGa9 displayed decreased LVESD and LVEDD relative to the TAC mice injected with circITGa9. *n* = 6. **P < 0.01. (E-G) Delivery of the mutated circITGa9 increased dp/dt (E), LVEF (F), and LVFS (G) in TAC mice relative to mice injected with circITGa9. *n* = 6–10. **P < 0.01. (H) Representative photographs of echocardiography in TAC mice injected with circITGa9 and the mutated circITGa9.

To confirm these binding activities, we performed site-directed mutagenesis to mutate the binding site (sequence provided in the Supplementary Materials, Table S3, CP90.hucircItga9-mut-TPM3), in which the nucleotides in close contact with TPM3 were mutated. We showed that mutation of the binding site did not affect the circularization of the mutant circITGa9 (Fig. 7A). In the coimmunoprecipitation assay, mutation of the binding site inhibited anti-TPM3 antibody precipitating circITGa9, measured by real-time PCR (Fig. 7B). On the other hand, the circITGa9 probe could no longer pull down TPM3 when the MCF cells were transiently transfected with the mutant construct (Fig. 7C).

We performed heart functional assays in TAC mice injected with the mutant construct and detected a significant decrease in LVESD and LVEDD, but subtraction of LVEDD by LVESD showed significant increase (Fig. 7D). We also found a significant increase in left ventricular pressure (dp/dt, Fig. 7E), LVEF (Fig. 7F), and LVFS (Fig. 7G). M-mode pictures showed abolished effect of circITGa9 on heart function in the TAC mice relative to the circITGa9 group (Fig. 7H).

### Intervention of cardiac fibrosis

**Fig. 8.**
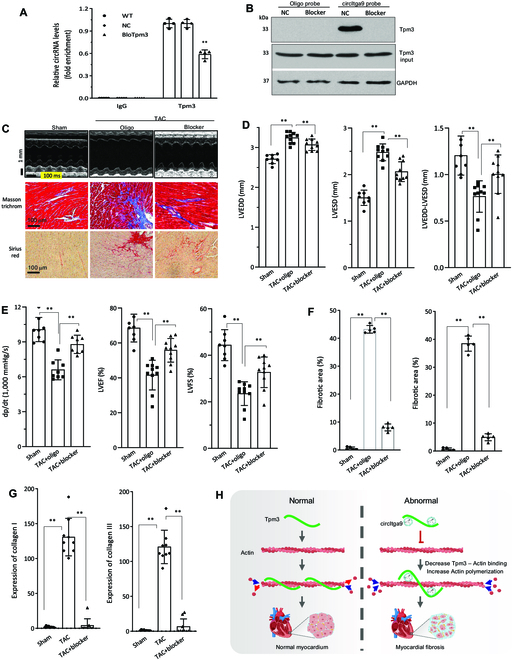
Blocking the roles of circITGa9. (A) Immunoprecipitation of circITGa9 by antibody against TPM3 was performed in MCF transfected with blocking oligo that inhibited the interaction of circITGa9 with TPM3. The precipitated circITGa9 was measured by real-time PCR. Transfection with the blocking oligo decreased the antibody precipitating circITGa9. *n* = 3. **P < 0.01. (B) TPM3 was pulled down by the circITGa9 probe in AC16 cells transfected with or without the blocking oligo. Transfection with the blocking oligo abolished the probe pulling down TPM3. (C) Representative photographs of echocardiography, Masson trichrome, and Sirius red staining from mice injected with or without the blocking oligo conjugated with gold nanoparticles. (D) Echocardiography analysis showed that TAC mice injected with the blocking oligo decreased LVESD relative to the TAC mice injected with the control oligo. *n* = 10. **P < 0.01. (E) Delivering the blocking oligo increased dp/dt (left), LVEF (middle), and LVFS (right) in TAC mice. *n* = 10. **P < 0.01. (F) Quantitation of Masson trichrome (left) and Sirius red (right) staining, showing delivering the blocking oligo decreased both staining in TAC mice relative to oligo control. *n* = 5. **P < 0.01. (G) Expression of collagen I (left) and collagen III (right). Delivering the blocking oligo decreased collagen expression in TAC mice relative to oligo control. *n* = 9. **P < 0.01. (H) circITGa9 interacted with TPM3 and promoted actin polymerization, leading to cardiac fibrosis.

To explore the potential of developing a therapeutic approach, we synthesized blocking oligos to block the binding of circITGa9 with TPM3 (Supplementary Materials). Real-time PCR analysis showed that blocking the binding sites of circITGa9 significantly decreased TPM3 antibody precipitating circITGa9 (Fig. 8A). Transfection with the blocking oligos inhibited the circITGa9 probe pulling down TPM3 (Fig. 8B). The typical M-mode pictures showed increased left ventricular contraction of the TAC heart subjected to blocking the interaction of circITGa9 with TPM3 (Fig. 8C).

In heart functional measurements of TAC mice injected with the blocking oligo, we observed a significant decrease in LVEDD and LVESD, as well as significant increase of subtraction of LVEDD by LVESD (Fig. 8D). We also detected a significant increase in the left ventricular pressure (dp/dt), an improved heart function showing significant increase in LVFS and LVEF (Fig. 8E). Furthermore, in the heart sections of TAC mice subjected to delivery of the blocking oligo, Masson trichrome and Sirius red staining showed decreased levels of collagen deposition (Fig. 8C). Quantitation showed that the levels of Masson trichrome and Sirius red were significantly decreased (Fig. 8F). Real-time PCR showed significantly decreased collagen I and collagen III in these heart tissues (Fig. 8G). In cell activity assays, we found that transfection with the blocking oligo decreased cell survival, adhesion, and migration of primary isolated cardiac fibroblasts (Fig. S6). Thus, our results demonstrated that circITGa9 formed a complex with TPM3 and γ-actin, which promoted actin polymerization and thereby regulated cardiac fibrosis. Based on these results, we have proposed a model to hypothesize how circITGa9, TPM3, and actin play roles in myocardial fibrosis (Fig. 8H).

## Discussion

In this study, our primary objective was to investigate the expression of novel circRNAs in patients with hypertrophy-associated heart diseases as well as in normal myocardium tissues. To achieve this, we employed deep circRNA sequencing techniques. To ensure the exclusivity of circRNAs in our analysis, we treated the samples with RNase R, effectively eliminating all linear transcripts. Each sample underwent extensive read-count analysis, with over 35 million read counts conducted. This deep sequencing approach enabled us to identify a remarkable total of 32,034 previously unreported circRNAs. While the identity of these novel circRNAs requires further validation, the use of RNase R treatment facilitated the detection of circRNAs even at extremely low abundance levels.

It has been reported that circRNA expression is spatially- and temporally-specific, and many reported circRNAs are expressed in organ-specific manners [3,26]. The tissue-specific expression of circRNAs may be due to the transcript regulation or splicing regulation [4,35]. This can cause aging accumulation of some circRNAs [4]. The dynamic RNA splicing during aging is observed in tissues and different species that results in alterations in the abundance of circRNAs [36–38]. Many genes contain a large number of exons, which allows for a variety of back splicing processes and can potentially generate a large number of circRNAs, depending on the availability of splicing factors needed to generate different circRNA isoforms. Thus, some circRNAs may only be expressed at specific time points or specific tissues when the necessary splicing factors are available. It is conceivable that the circRNAs reported up-to-date may only represent a portion of the total circRNAs. Indeed, in the diseased cardiac tissues, we detected a large number of novel circRNAs. It appears that the number of circRNAs are far more than the number of genes, as one gene may generate many circRNAs with different arrangements/permutations and combinations.

ITGA9 plays essential roles in mediating cell-matrix interaction by binding to extracellular matrix proteins [39]. This gene contains 28 exons, and with potential back-splicing, it is possible for the gene to produce many circRNA isoforms. Previous studies have reported 10 circRNA isoforms generated by ITGA9 [3,31]. Our study showed that none of them were expressed at significant levels in regulating heart functions. Interestingly, we detected a novel isoform, hg38_circ_0030600, expressed at low levels in normal heart tissues. However, during cardiac remodeling, this circRNA expression increased 38-fold and was the most up-regulated circRNA. This result suggests that hg38_circ_0030600 plays an important role in cardiac remodeling. GO analysis revealed that the gene (ITGa9) producing hg38_circ_0030600 was involved in all 13 functional characteristics enriched at significant levels due to the differential expression of circRNAs, all of which appeared to be associated with cardioactivities. KEGG analysis revealed 7 pathways reached significant levels as a result of the differentially expressed circRNAs, all of which are associated with cardioactivities, suggesting successful sequencing results. Among these 7 pathways, hg38_circ_0030600 was ranked number 1 of 3 pathways, including pathways of hypertrophic cardiomyopathy, dilated cardiomyopathy, and cell adhesion molecules. These results strongly suggest that hg38_circ_0030600 plays essential roles in cardiac remodeling. Clinical analysis also showed significant up-regulation of hg38_circ_0030600 in hypertrophic patients. Future studies may be helpful to target hg38_circ_0030600 as an intervention in patients with heart disease.

In this study, we demonstrated that expression of circITGa9 increased cardiac fibrosis by binding to TPM3 and modulating actin polymerization. Tropomyosins are a large family of alpha-helical coiled coil proteins in the cytoskeleton that play critical roles in regulating the function of actin cytoskeleton by integrating into and stabilizing actin filaments. Through its interactions with actin and the troponin complex in the sarcomere, tropomyosin regulates muscle contraction and relaxation in a calcium-dependent manner. When levels of calcium are low in the cytoplasm during muscle relaxation, tropomyosin blocks the active site on filamentous actin, onto which myosin would bind and generate a force. Upon activation, the concentration of calcium in the cytoplasm increases, allowing calcium to bind to troponin and change the shape of tropomyosin. Due to the conformational change, tropomyosin rolls away from actin, allowing myosin to bind actin, which generates force that shortens the sarcomere in cardiac contraction. The binding of tropomyosin can inhibit assembly of actin filaments and actin polymerization by restricting the activity of Arp2/3 complex [40]. Our study showed ectopic expression of circITGa9 promoted actin polymerization. Since circITGa9 binds TPM3, it is likely that circITGa9 bound TPM3 and inhibited TPM3 regulation of actin polymerization. It has been reported that decreased actin polymerization and cytoskeleton assembly resulted in down-regulation of collagen expression and impairment of the fibrosis-associated TGF-β signal pathway, whereas increased actin cytoskeleton assembly promoted TGF-β signaling and collagen expression [41]. Increased actin polymerization promoted tissue fibrosis [34]. Our results are consistent in that expression of circITGa9 promoted actin polymerization and increased collagen levels. Furthermore, our results revealed cardiac fibrosis as a consequence of circITGa9 promoting actin polymerization and collagen levels, by binding to TPM3. circITGa9 appears to be a link between actin polymerization and cardiac hypertrophy and fibrosis.

There are multiple isoforms of tropomyosin that are differentially expressed and exhibit developmental and tissue- or cell-specific functions. Although the isoform TPM3 is expressed in muscle and nonmuscle cells, it plays a central role in striated muscle contraction by binding to the troponin complex in a calcium-dependent manner and modulating the dynamic transformation of G-actin and F-actin. Rapid polymerization and depolymerization of actin filaments in the cytoskeleton network are essential to facilitate cell activities, including contraction and migration [33,42,43]. By binding to actin filaments, tropomyosins are able to control actin polymerization and depolymerization, a process essential for cardiac activity. Deregulation of tropomyosin function may lead to cardiac diseases, such as hypertrophy and cardiac fibrosis. It is known that tropomyosin mutation results in hypertrophic cardiomyopathy and dilated cardiomyopathy [44]. Our study yielded significant insights into the role of circITGa9 in cardiac hypertrophy and fibrosis, primarily through its interaction with TPM3 and subsequent activation of actin polymerization. We demonstrated that circITGa9, by binding to TPM3, promotes cardiac hypertrophy and fibrosis. Conversely, silencing endogenous circITGa9 or inhibiting its binding to TPM3 effectively released TPM3 from its detrimental effects, thereby mitigating cardiac hypertrophy and fibrosis. These findings have promising implications for potential clinical applications. Developing safe and effective technologies that target cardiac hypertrophy and fibrosis, specifically focusing on circITGa9 and its interaction with TPM3, would be a crucial area of future research and development.

## Methods

The general methods were performed as previously described [45–49,50]. The specific details are provided in the Supplementary (Materials and General Methods).

### Construct generation, siRNA, and primer design

The plasmid circITGa9 was generated as shown in the Supplementary Materials. The vector plasmid contains a Bluescript backbone, with one cytomegalovirus promoter driving green fluorescent protein expression and another cytomegalovirus promoter driving the circRNA-forming fragments. As a mock control, the circITGa9 insert sequence was replaced with a nonrelated random sequence. The construct circITGa9-lin was generated in which the intron sequences for circularization signal were deleted from the circITGa9 construct. Thus, circITGa9-lin would express the linear transcript without circularization. The construct circITGa9-mut was generated in which a point mutation was created in the circITGa9 construct by site-directed mutagenesis. The product of 51 circITGa9 (circITGa9-mut) retained the circularization capacity, but the mutated circRNA could no longer bind to TPM3. The sequences of all constructs, probes, primers, and siRNAs are provided in Tables S3 to S5.

### Plasmid or siRNA-PEG-Au NP synthesis and delivery

For the synthesis of circITGα9 plasmid or siRNA conjugate, 20 nmol of plasmids or thiol-modified siRNAs was dissolved in 800 μl of RNase-free water. The mPEG-SH (PG1-TH-2k, Nanocs, New York, NY) was mixed with plasmid at a 1:20 molar ratio. Gold nanoparticles (Au NP, 10 nm; Cytodiagnostics, Burlington, Ontario, Canada) were mixed with plasmid or siRNAs-PEG conjugate at weight ratio of 1:1 for conjugation. The mixture was gently shaken at 60 °C for 30 min and transferred into a syringe. The circITGα9 plasmid or siRNAs-PEG-Au conjugate was administered intraperitoneally in a volume of 100 μl twice a week as previously described.

### Human heart specimens

The clinical study was carried out in accordance with The Ethics Code of the World Medical Association (Declaration of Helsinki). Formal informed consents were obtained from all patients in the study prior to enrollment. Heart samples were obtained from heart donations or clinical surgeries. Each sample was immediately immersed into liquid nitrogen. A total of 73 specimens from patients with hypertrophy-associated heart diseases and 25 specimens from heart donations without heart disease records were obtained. The information of these heart patients was described in Tables S9 and S10. Heart tissues without detectable cardiovascular disease were collected from 25 individuals who deceased from noncardiac-related causes. All samples were collected 0.5 to 6 h after the patient’s death. During surgery, the heart tissues were obtained from the left ventricular free wall and parceled into 2 or 3 small pieces. The first fragment was collected into cryovials, snap-frozen in liquid nitrogen, stored at −80 °C, to be used for RNA or protein extraction. The second fragment was fixed in 4% formaldehyde for 2 to 3 days, followed by wax embedding.

### Animal models

All animal experiments adhered to the guidelines and regulations approved by the Animal Care Committee of Sunnybrook Research Institute. We induced PO-induced cardiac hypertrophy in mice using a modified TAC procedure, as previously detailed [52]. The success of aortic banding was confirmed visually by assessing differential carotid pulsation. Additionally, the effectiveness of the PO model was verified by measuring carotid artery flow velocities with Doppler ultrasound. Only mice with a right carotid (RC)/left carotid (LC) flow ratio exceeding 5 were included in subsequent assays. Sham-operated mice underwent the same surgical procedure with anesthesia but without aortic banding.

For in vivo function assays examining the role of circITGa9, 8-week-old C57BL6 mice underwent TAC and were injected intraperitoneally with the plasmids of the control vector or circITGa9 (50 μg/each) twice a week for 4 weeks. For intervention studies, 12-week-old mice were chosen for TAC model generation and were injected with control oligo and either circITGa9 siRNAs or blocking oligos (5 μg/each) twice a week for 12 weeks. Either the plasmids or the siRNAs and oligos were conjugated with PEG and Au NP that formed the conjugated complexes before injection. Conjugation of the plasmids with PEG-Au NP was performed as previously described [50]. Controls also included a group of sham mice. Four weeks after injection, all mice were assessed for cardiac functions and sacrificed for tissue collection. Upon harvest, the hearts were halved. The upper portion of the heart was promptly frozen for subsequent real-time PCR analysis or prepared for frozen sections, followed by circITGa9 in situ hybridization. The lower half was fixed in 10% buffered formalin, embedded in paraffin, and sectioned into 5-μm sections for subsequent staining.

### His-tag pulldown assay

His-tag pulldown assay was performed following the manual of His purification from Qiagen Ni-NTA. Briefly, MCF cells were cotransfected with His-tagged (His-Tpm3) plasmids and either vector or circITGa9 plasmids. Cell lysates were prepared in lysis buffer (100 mM NaH_2_PO_4_, 10 mM tris-Cl, 8 M urea, pH 8.0) and incubated with prewashed Ni-NTA beads (washed twice with 3 ml of lysis buffer) at room temperature for 1 h. The beads were pelleted by centrifugation at 3000 rpm for 2 min to remove supernatant. The beads were washed once with 5 ml of lysis buffer and twice with wash buffer (100 mM NaH_2_PO_4_, pH 6.3, 10 mM tris-Cl, 8 M urea), from which the supernatant was removed after centrifugation at 3,000 rpm for 2 min. Bound proteins were eluted with 200 μl of elution buffer (100 mM NaH_2_PO_4_, pH 4.5, 10 mM tris-Cl, 8 M urea). The samples were kept at −80 °C until further assays.

### Statistical analysis

All experiments were performed in triplicate or more. Data were presented as mean with SEM. Statistical analyses were calculated using Prism 8 (GraphPad Software: La Jolla, CA). Student *t* test was performed to assess the difference between 2 groups. In multiple comparisons, one-way analysis of variance followed with the Tukey test were used. The levels of significance were set at *P < 0.05 and **P < 0.01.

### Declaration

Our study adheres to the principles of the Declaration of Helsinki. The research protocol has received approval from the Animal Care Committee of Sunnybrook Research Institute, and informed consent has been acquired from the subjects. Additionally, the analysis of human heart tissues was authorized by the Ethics Committee of Guangdong General Hospital.

## Data Availability

The data underlying this article will be shared on reasonable request to the corresponding authors.

## Supplementary Materials

Supplementary 1 Supplementary-Materials and General Methods Figs. S1 to S6 Tables S1 to S10
