# Supplementary material for: A Novel Circular RNA circITGa9 Predominantly Generated in Human Heart Disease Induces Cardiac Remodeling and Fibrosis
Source: Research (Wash D C). 2024 Feb 6;7:0303. doi: 10.34133/research.0303 (PMC10845611; doi:10.34133/research.0303)
Supplement: Supplementary 1 — Supplementary-Materials and General Methods Figs. S1 to S6 Tables S1 to S10 [file research.0303.f1.zip › circItga9 Supplementary-Dec 10, 2023.pdf]

A

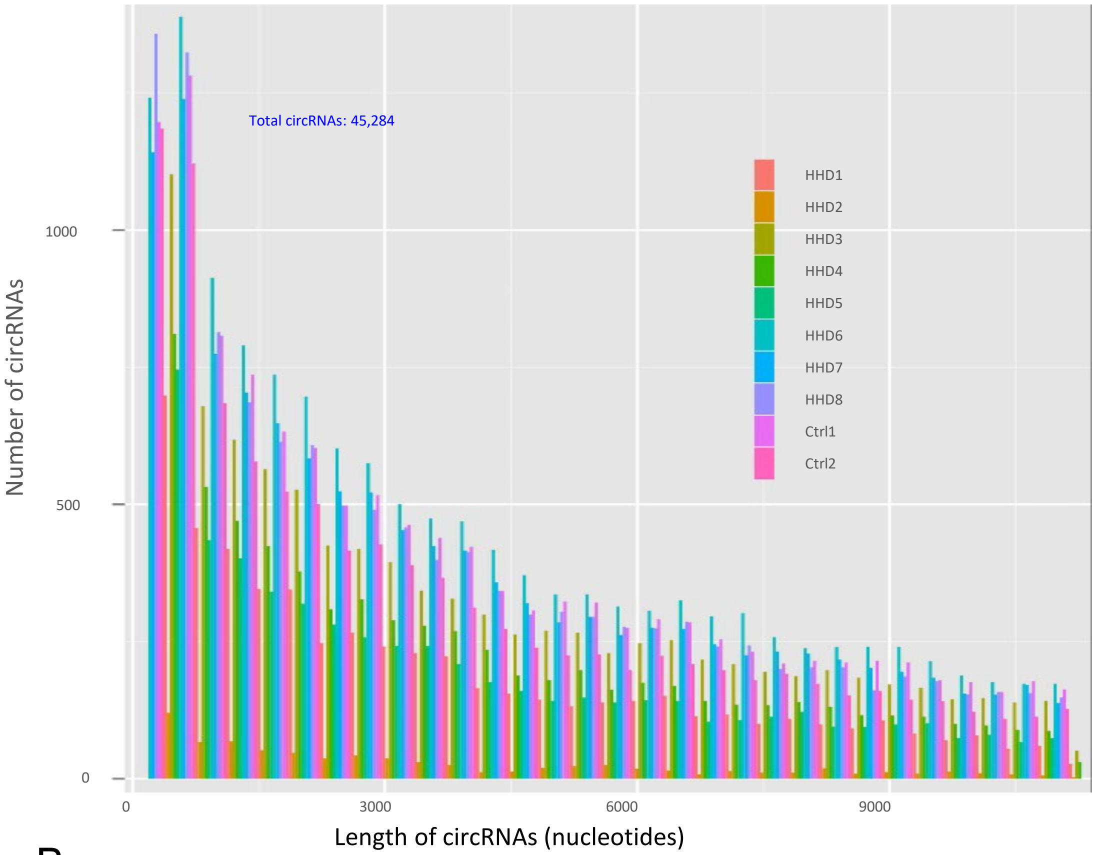

B

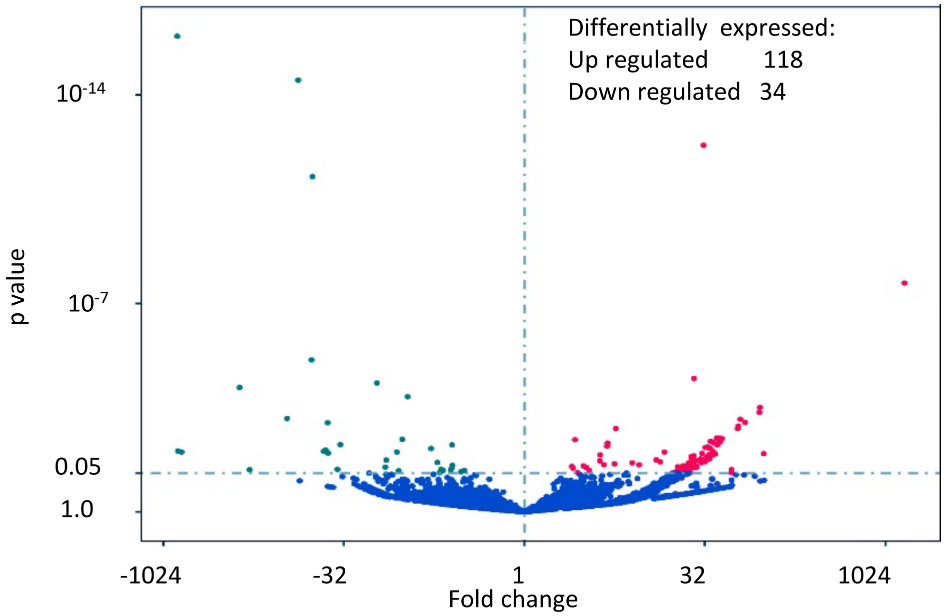

Supplementary Fig S1. Sequencing circRNAs in heart hypertrophy

(A) The length distributions of all circRNAs identified.

(B) Volcanoplot showing the up- and down-regulated circRNAs including their fold-change and p-values.

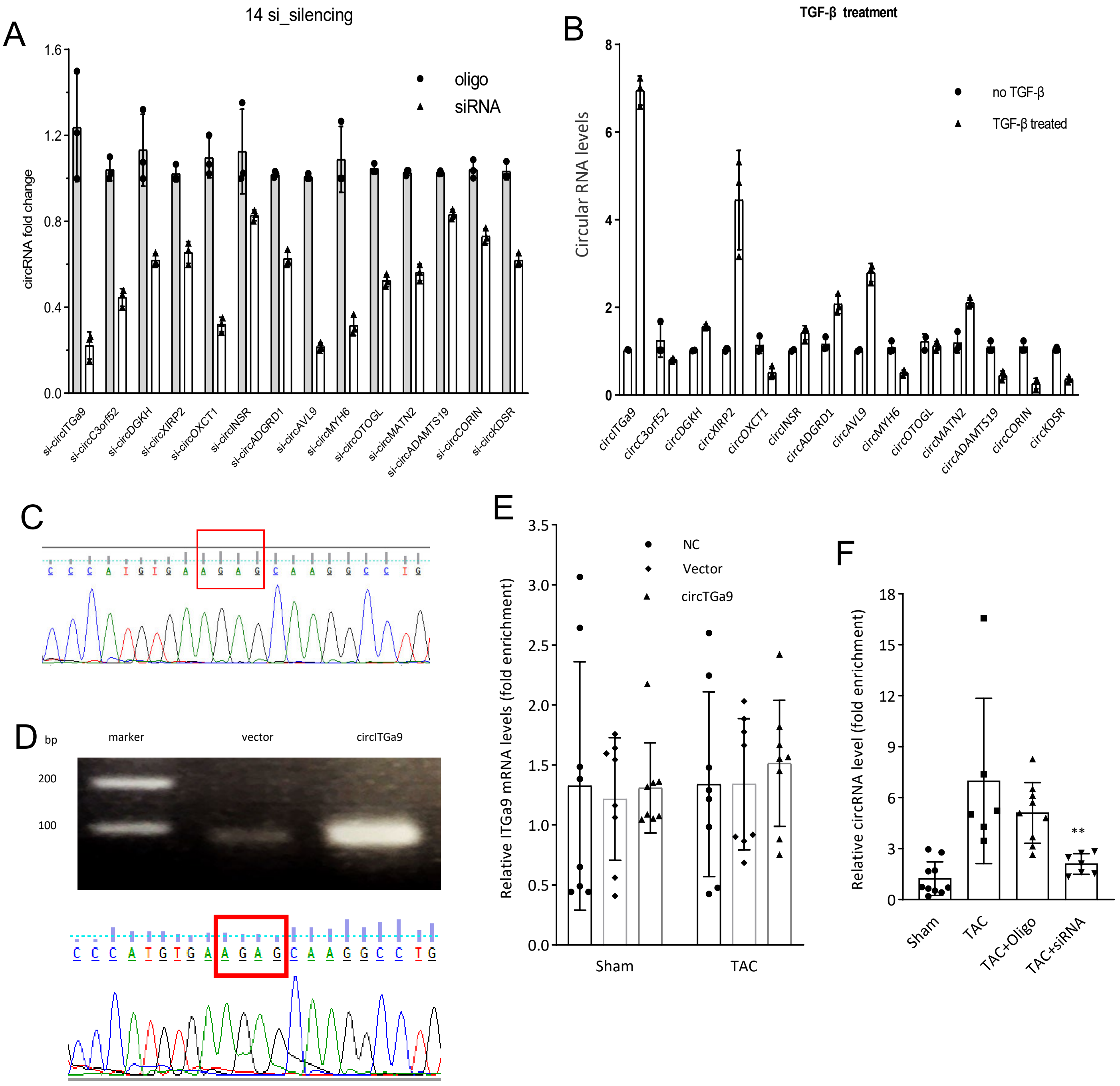

Fig S2. Silencing circITGa9

(A) HCF cells were transfected with siRNAs targeting the junction sequences of 14 circRNAs as indicated. Silencing circITGa9 showed the most fold-changes. n=4.

(B) HGF cells were treated with or without TGF-b, followed by measurement of 14 circular RNA levels. circITGa9 expression increased the most following TGF-b treatment. n=6. *p<0.05.

(C) PCR was performed to confirm the presence of the circITGa9 junction sequence. Sanger-sequencing revealed the correct junction sequence of circITGa9.

(D) Upper, MCF cells were transfected with circITGa9 or a control vector followed by amplification of the junction sequence of circITGa9 by RT-PCR with the divergent primers. Lower, the PCR was subjected to DNA sequencing, confirming the correct junction sequence of circITGa9.

(E) Expression of ITGa9 linear mRNA was not affected by circITGa9 expression.

(F) Delivery of circItga9 siRNAs decreased circITGa9 levels. ** p<0.001.

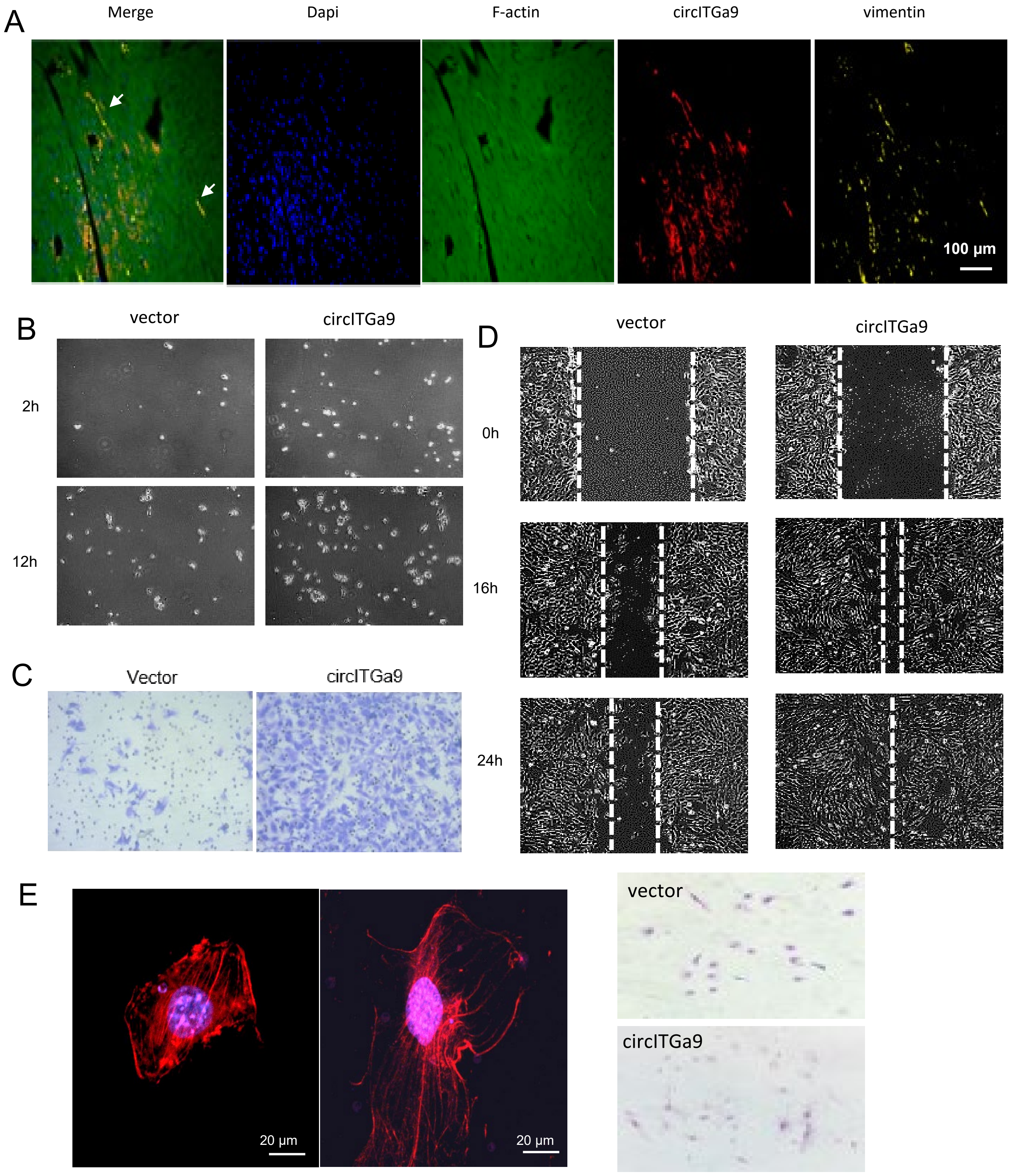

Fig S3. Co-localization of circITGa9 and cardiac fibroblasts. (A) Overexpression of circITGa9 increased adhesion (B) and migration (C, chambering migration and D wound healing migration). (E) Cardiac-fibroblast morphology change following circITGa9 overexpression (left, confocal microscopic examination; right, light microscopic examination).

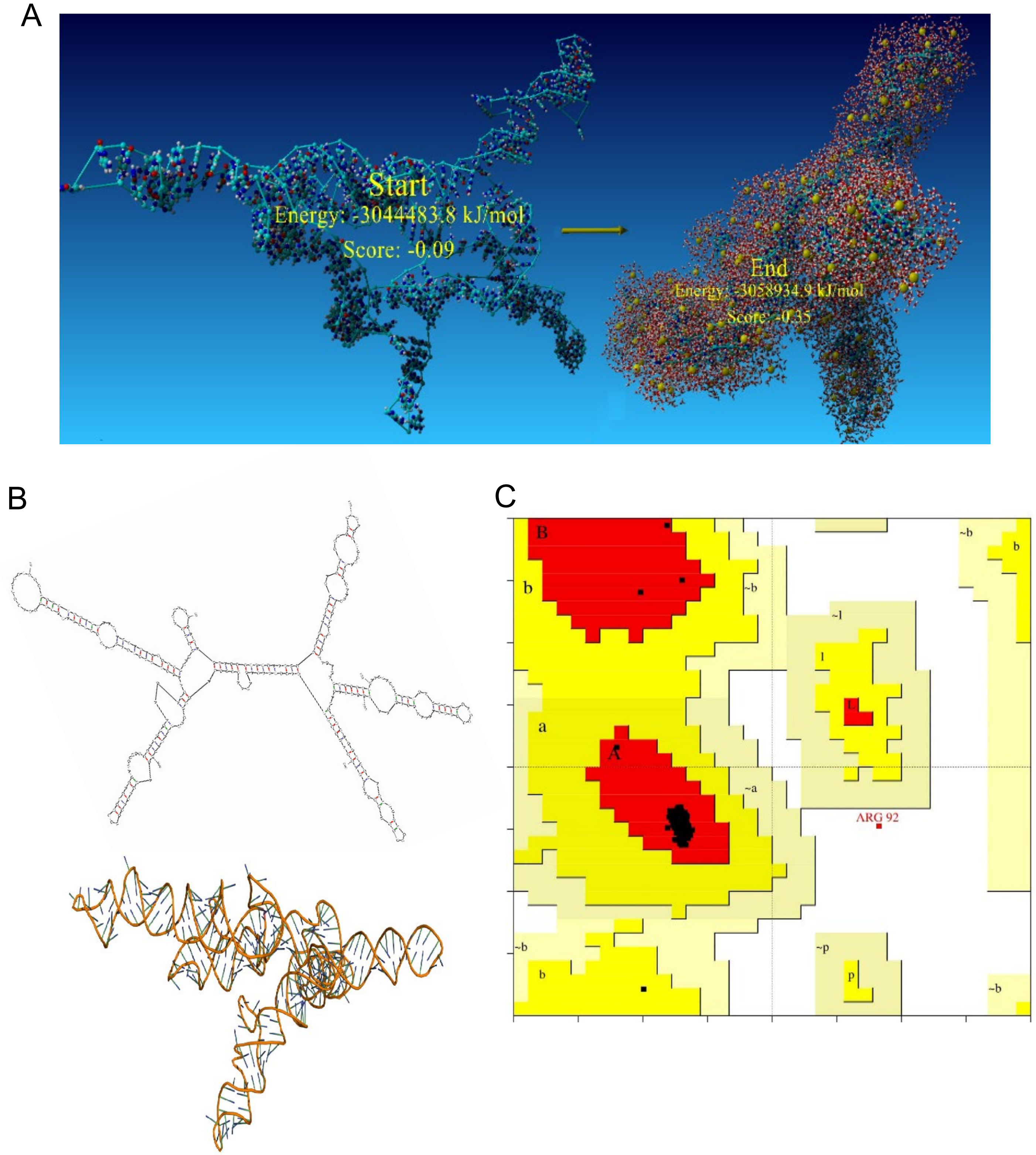

Fig S4. Computational analysis of circITGa9 and TPM3 structure.
(A) YASARA representation of energy minimized 3D circITGA9 RNA.
(B) 2D and 3D representation of circ-ITGa9 RNA. Upper, 2D RNA structure of circITGa9 was generated by Mfold. **Lower,** A 3D visualization of circITGA9 RNA produced in PyMol from estimates of 3D structure by RNA composer using the secondary structure delineated in dot bracket notation.
(C) The Ramachandran plot calculation of the psi/phi angle distribution of TPM3 model using PROCHECK validation server showing the stereochemical quality of the structure.

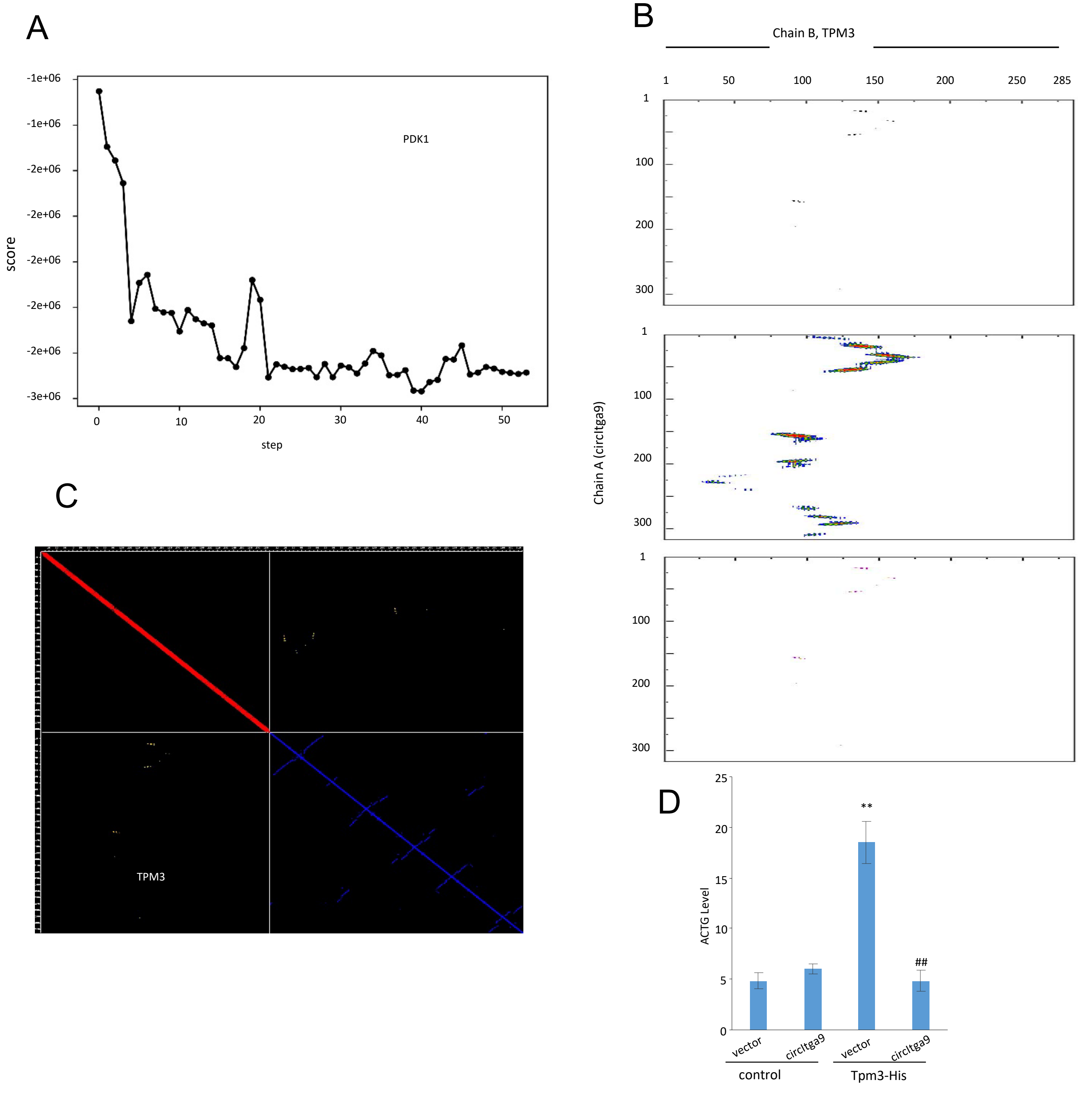

Fig S5. Computational analysis of circITGa9 interacting with TPM3.
(A) Refinement of the best docked circITGa9-TPM3 model showing MC score vs. steps of simulation.
(B) **Contact Maps of circITGa9-TPM3 complex. (a).** The "classical black and white map" presents a black dot at the crossover of two residues i and j, belonging to cir-ITGa9 and TPM3, if any atom of the two residues are closer than the cut-off distance (3.5 Å). (**b**). The "distance Range map" illustrating inter-molecular contacts at increasing distances, as colored dots (7 Å, 10 Å, 13 Å and 16 Å indicated in **Red**, **yellow**, **green**, and **blue** color) respectively. (**c).** The "property map" with each contact colored according to the physico-chemical nature of the two interacting residues, **yellow** = hydrophilic-hydropholic**, violet** = hydropohilic-hydrophilic, **green** = hydrophobic-hydrophobic.
(C) **Residue-level resolution contact map of CircITGa9-TPM3 complex.** CircITGa9 in complex with TPM3. In a contact map picture, protein contacts are displayed in Red, RNA contacts are blue, and the protein-RNA interface contacts are displayed in yellow. Distance range (3.5 Å).
(D) The Western blot of circITGa9 affecting TPM3-His binding to ACTG (Fig 6J) was repeated three times and the intensities were quantified.

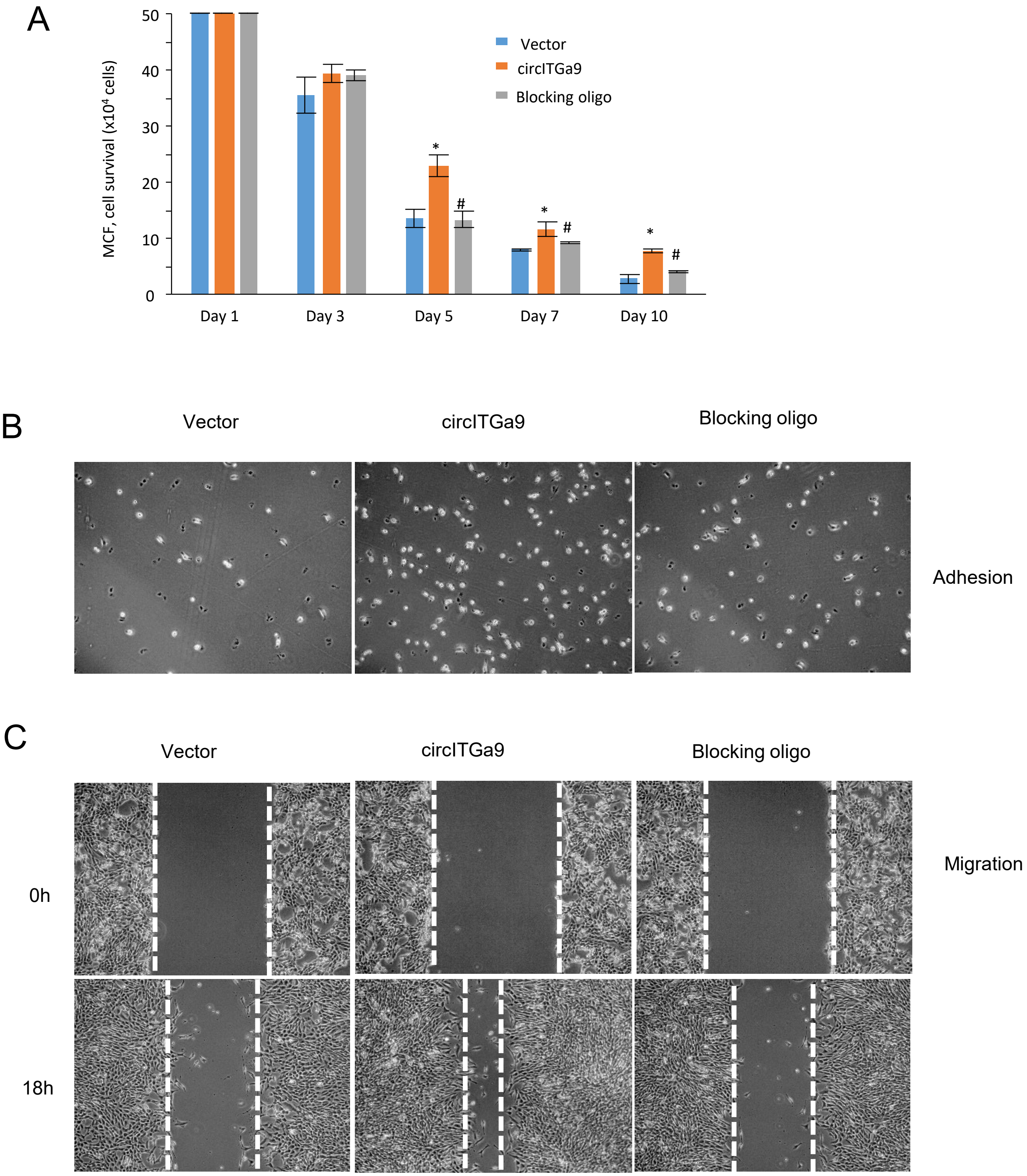

Fig S6. Blocking circITGa9-TPM3 interaction decreased survival (A), adhesion (B) and migration (C) of cardiac fibroblasts isolated from the mouse heart.

revoked without the express written consent of both parties. This License shall be governed and construed, and any dispute arising hereunder resolved, in accordance with the laws of the District of Columbia, United States of America, without resort to the conflicts of laws principles thereof.

Burton B Yang

Author's Name

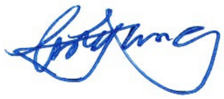

Author's Signature
