# Supplementary material for: A Novel Circular RNA circITGa9 Predominantly Generated in Human Heart Disease Induces Cardiac Remodeling and Fibrosis
Source: Research (Wash D C). 2024 Feb 6;7:0303. doi: 10.34133/research.0303 (PMC10845611; doi:10.34133/research.0303)
Supplement: Supplementary 1 — Supplementary-Materials and General Methods Figs. S1 to S6 Tables S1 to S10 [file research.0303.f1.zip › Contents of circITGa9 Supplmentary Information.pdf]

Contents of circItga9 Supplementary Information

1. Supplementary-Materials and General Methods pages 1-5
2. Supplementary Figures 1-6 pages 6-11
3. Supplementary Tables 1-10 pages 12-42

Supplementary-Materials and General Methods

Materials

The monoclonal antibody against Tropomyosin 3 (CAT# ab113692) 1:1000 G-M was from Abcam. Horseradish peroxidase-conjugated goat anti-mouse and goat anti-rabbit IgG, RNA reverse transcription and PCR kits were purchased from Bio-Rad. DNA and RNA isolation kits were purchased from Geneaid. ECL Western blot detection kit was from Millipore. Biotin Chromogenic Detection kit was purchased from Thermo Scientific. Masson's Trichrome staining kit was from American MasterTech.

Circular RNA sequencing, GO enrichment analysis, KEGG analysis

Human heart tissues from patients with hypertrophy and normal myocardium tissues were subject to RNA isolation and library construction. A total of 5 µg RNA per specimen was used as input material. The RNA samples were subjected to RNase R treatment to degrade linear RNAs and depletion of rRNA by using Epicentre Ribo-zero™ rRNA Removal Kit (Epicentre, USA). The rRNA-depleted RNAs were further treated with RNase R (Epicentre, USA) and subjected to Trizol extraction. The RNase R-resistant circRNAs were analyzed on 1% agarose gels to monitor potential degradation and contamination. RNA purity was confirmed by NanoPhotometer® spectrophotometer (IMPLEN, CA, USA). The RNAs were fragmented using divalent cations under elevated temperature in NEBNext® UltraTM RNA Library Prep Kit for Illumina® (NEB, USA). Reverse transcription, adenylation of 3' ends, adaptor ligation, PCR amplification, and purification were performed following the manufacturer's instruction. The generated cDNA library was sequenced and analyzed Novogene (www.novogene.com) using an Illumina HiSeq 2500 System. Each sample was performed independently. All 125 bp/150 bp paired-end reads were generated. Reference genome and gene annotation were built using Bowtie v2.0.6. Paired-end clean reads were aligned to the reference genome by using TopHat v2.0.9. Unmapped reads were kept and 20-mers from 5' and 3' end of these reads were extracted and aligned independently to identify circRNAs. Every circRNA was recorded by at least two reads spanning a head-to-tail splice junction in each sample. All potential circRNAs were compared with the reported circRNAs (*1*) to determine known and unknown circRNAs. Compared to normal myocardium, the numbers of differentially expressed circRNAs at a 2-fold cut-off were calculated for each sample. The significant difference was then determined between the hypertrophy and normal myocardium tissues. The analysis was performed by the circRNA sequencing company Novogene. They used two algorithms to avoid false positives (*2, 3*).

Cell culture and functional assays

Each cell line was cultured as follows: MCF cells were cultured with DMEM supplemented with 10 % FBS and 1 % antibiotics. AC16 cells were cultured with DMEM/F12 supplemented with 12.5% FBS and 1% antibiotics. HCF cells were cultured in DMEM supplemented with 15% FBS and 1% antibiotics. All cells were cultured at 37°C in a humidified atmosphere of 5% CO_2/95% air.

Cell proliferation assay. Cells were seeded at $1x10^4$/well confluency in 10% FBS basal medium in 12-well culture plates at 37 °C. Cells were harvested and counted daily by using Coulter Counter under an inverted microscope.

Cell survival assay. Cells were seeded in 12-well plates at $5x10^4$ cells/well confluency and maintained at 37 °C for 12 h. Following cell attachment, the medium was replaced with serum-free DMEM. Cells were harvested and counted every other day. The harvested cells were stained with Trypan Blue and cell number was counted by Coulter Counter under an inverted microscope.

Cell migration assay. Cells were seeded in 6-well plates ($7x10^5$ cells/mL/well) and cultured at 37 °C for 12 h. A wound was created by scratching the cell monolayer in a straight line with a sterile P-200 pipette tip. The cultures were rinsed twice with 1 ml of sterile 1x PBS to remove the floating cells and debris, followed by addition of 2 ml medium (10% FBS containing DMEM with 1% antibiotics). Photos were taken at 0 h, 6 h, 18 h and 24 h. Representative photos were used for data illustration.

Cell adhesion assay. Cells were seeded in 35 mm Petri dishes ($6x10^5$ cells/well) and cultured at 37 °C for 2 h, 6 h, and 18 h, respectively. Plates were taken out at each time points, and unattached cells were washed away twice with 1x PBS. Representative pictures were taken and the adhered cells were harvested and counted by Coulter Counter under an inverted microscope.

Cardiac function assessment and fibrosis staining

Mice were anesthetized with 2% isoflurane inhalation before and during undergoing transthoracic echocardiography and invasive hemodynamic assessment. Transthoracic echocardiography was performed and analyzed using a Vevo 2000 high-resolution imaging system in a blinded manner. The system was equipped with a 40-MHz transducer to measure left ventricular ejection fraction (LVEF), left ventricular fractional shortening (LVFS), left ventricular end diastolic diameter (LVEDD), left ventricular end systolic diameter (LVESD), and dP/dt.

Fibrosis staining was performed by Masson's Trichrome staining and Sirius Red staining. Masson's Trichrome staining was performed using the Masson's Trichrome stain kit (American Master Tech). For Sirius red staining, mouse heart sections were de-waxed and hydrated, and stained with Weigert's haematoxylin for 8 min. The sections were then rinsed with running tap water, stained in 0.1% picrosirius red for 1 h, and washed in 0.1% acetic acid.

Immunofluorescence staining

Cells cultured on BD culture slides were fixed with cold methanol for 10 min. The slides were then blocked with 10% goat serum and incubated with primary antibody in TBS containing 10% goat serum at 4 °C overnight. The slides were washed and probed with goat anti-mouse or goat anti-rabbit Alexa Fluor 488, 546, or 647 at room temperature for 2 h. Fluorescent phalloidin 488 or phalloidin 546 was used to stain F-actin to show cell structure. DAPI was used to stain DNA to detect nuclei. Images of the staining were performed using Zeiss Z1 Axio confocal laser scanning microscopy.

Fluorescence in situ Hybridization (FISH)

In the fluorescence in situ hybridization (FISH) procedure, Alexa 488 or Cy5-labeled DNA oligo probes targeting circITGa9 were generated using a fluorescence PCR labeling kit (Biolynx). The labeled probes were briefly heated to 95°C for 2 minutes and promptly chilled on ice to prevent reannealing. As a negative control, a scramble sequence was also labeled using the same method. Fixed samples were dehydrated through sequential 1-minute washes in 70%, 95%,

and 100% ethanol. Following dehydration, the samples were air-dried and then subjected to a 30-minute pretreatment with a hybridization solution at 55°C. Pre-hybridized slides were subsequently incubated with 50 nM fluorescence-labeled DNA probes in a hybridization buffer for 3 hours at 55°C. This was followed by a series of washes using saline-sodium citrate (SSC) buffers. Immunofluorescence staining was carried out after the samples were blocked with TBS containing 10% goat serum for 30 minutes.

Real-time PCR

Cells or tissues were harvested for total RNA extraction using the Geneaid RNA mini kit or TRIzol. Total RNAs were subject to RNase R treatment to remove linear RNAs or directly utilized for reverse transcription to synthesize single stranded cDNA. Real-time PCR was performed with Bio-Rad SYBR Green PCR Kit using 2 μl cDNA as a template with two appropriate primers. GAPDH or the small nuclear RNA U6 was used as a control to normalize the levels of cDNA used.

Western blotting

Protein expression levels were determined using Western blotting, following established methods (*4*). In brief, proteins were extracted from both cells and tissues. The lysates were subjected to separation through sodium dodecyl sulfate-polyacrylamide gel electrophoresis (SDS-PAGE). The separated proteins were then transferred onto a nitrocellulose membrane in 1x Tris/glycine buffer containing 20% methanol, employing an 80 V charge at 4°C for 2 hours. The membrane was subsequently treated in a buffer solution comprising 10 mM Tris-Cl (pH 8.0), 150 mM NaCl, 0.05% Tween-20, and 5% non-fat dry milk powder for 1 hour. It was then incubated with primary antibodies overnight at 4°C. The following day, the membranes were subjected to a thorough washing with the aforementioned buffer, repeated three times for 30 minutes each. Subsequently, they were incubated with secondary antibodies at room temperature for 2 hours. After further washing, the bound antibodies were visualized using an ECL detection kit. To ensure equal loading of proteins in each sample, the same membranes were re-probed with a rabbit anti-γ-actin monoclonal antibody (Abcam) or GAPDH.

RNA and protein immunoprecipitation assay

The assay was performed using an antibody against the protein of interest. Briefly, magnetic beads (100 μl) were washed in PBS-T (PBS + 0.1% Tween 20) buffer and incubated with 1 μg of the primary antibody at room temperature for 15 min. Meanwhile, protein lysates were prepared in native conditions. After centrifugation, protein concentrations were determined and equalized with the same buffer. The protein lysates were incubated with the antibody-containing beads for 1 hour. Subsequently, the magnetic beads underwent three washes with PBS-T and were resuspended in either TRIzol (for RNA extraction) or 2× Laemmli buffer (composed of 0.125 M Tris-HCl, 4% SDS, 20% glycerol, 10% 2-mercaptoethanol, 0.004% bromphenol blue, pH 6.8) for protein isolation. The isolated RNAs were used for real-time PCR analysis, while the isolated proteins were employed for Western blotting.

RNA pulldown assay

Cells or tissues were lysed in a co-immunoprecipitation (co-IP) buffer and subsequently incubated with 3 μg of biotinylated DNA probes designed against circITGa9 at room temperature for 2 hours. Streptavidin C1 magnetic beads (50 μl), sourced from Invitrogen, were added to each probe-containing reaction and further incubated at room temperature for 1 hour. The beads were then collected and subjected to thorough washing with co-IP buffer, repeating this process five times. The proteins that had interacted with the circRNAs and were pulled down by the

probes were subsequently employed for further analyses, including mass spectrometry or Western blotting.

Mass Spectrometry Analysis

Mass Spectrometry and analysis were performed in SPARC Molecular Analysis Centre, The Hospital for Sick Children, Toronto, Ontario, Canada. Streptavidin magnetic beads containing the pulldown samples were pretreated using 10mM DTT at 60°C for 1 hr and followed by incubating with 20 mM iodoacetamide at room temperature for 45 min in dark. After treatments, samples were digested with trypsin (Pierce) at 37°C overnight according to 2 ug per sample at a 1:50-1:100 ratio. Trypsinized samples were resuspended in 0.1% formic acid and subjected to LC-MS/MS according to standard protocol in SPARC Centre. Each sample was analyzed using an EASY-nLC 1200 nano-LC system coupled to a Thermo Scientific Orbitrap Fusion Lumos Tribrid mass spectrometer. Peptides were separated using a 75 µm x 50 cm PepMax RSLC EASY-Spray column filled with 2 µM C18 beads (ThermoFisher, San Jose, CA), pressure 900 Bar, 60°C. All mass spectra were analyzed with Proteome Discoverer version: 2.5.0.400. MS/MS spectra were searched against the Human Uniprot protein sequence database. Precursor mass tolerance was set to 10 ppm for the first search where initial mass recalibration was completed and for the main search. Product fragments were searched with a mass tolerance 0.6 Da. Enzyme was set to trypsin in a specific mode and a maximum of 3 missed cleavages was allowed for searching. Carbamidomethylation of cysteines was searched as a fixed modification, while oxidation of methionines and acetylation of protein N-terminal were searched as variable modifications. Total MS/MS spectral counts were used for identification of differential protein between vector control and circITGa9 overexpressed group.

Bioinformatics prediction

To determine the possible interaction of circITGa9 with TPM3, 20,000 models were generated using a protein-RNA docking tool NPDock server (*5*). NPDock server was used in combination with GRAMM for global macromolecular docking, scoring with a statistical potential, clustering followed by refinement of best scored docked complexes from three biggest clusters. Molecular interaction between circITGa9 and TPM3 was performed and refinement of the most promising models. Analysis of residue-level resolution contact map for circITGa9-TPM3 complex was also done along with calculation of distances between interacting residues. The distance-based and residue-level resolution contact maps of circITGa9-TPM3 docked complex was determined using RNAmap2D (*6*) and COCOMAPS (*7*) tools. Contact distances were calculated between Cα atoms of protein residues and O5' atoms of RNA strands. Two residues were considered in contact when their O5'–Cα distance was less than 10 Å. Distance based approach was used to identify the binding site residues/nucleotides for the protein-RNA complexes using a specific cut-off value. Two atoms (one in RNA and another in protein) were considered to be interacting with each other if the distance between them was < 3.5 Å.

Actin polymerization assay

Actin polymerization was performed with Actin Polymerization Biochem kit (Cytoskeleton, Inc) according to the instructions from the manufacturer. Pyrene actin was dissolved in G-buffer containing ATP at the concentration of 0.4 mg/ml on ice for 1 h to depolymerize actin oligomers. The reagent was subject to centrifuge at 14,000 rpm at 4ºC for 30 min to obtain G-actin in the supernatant. The supernatant was transferred to a 96-well plate (200 ul per well). Cell lysates were prepared in an actin compatible buffer containing 20 mM HEPES, 20 mM NaCl and protein inhibitors, followed by centrifugation at 150,000xg at 4ºC for 1 h. 20 ul of the supernatant from the lysates were added in each well containing G-actin reagent. Actin polymerization buffer was

added in the wells to initiate the reaction. The reaction occurred for 120 cycles, 60 seconds in each interval and scanned with Multiscan Spectrum (BioTek Synergy) in the range of ex.350 nm and em.410 nm.

References

1. J. Salzman, R. E. Chen, M. N. Olsen, P. L. Wang, P. O. Brown, Cell-type specific features of circular RNA expression. *PLoS Genet* **9**, e1003777 (2013).
2. L. Wang, Z. Feng, X. Wang, X. Wang, X. Zhang, DEGseq: an R package for identifying differentially expressed genes from RNA-seq data. *Bioinformatics* **26**, 136 (Jan 1, 2010).
3. M. I. Love, W. Huber, S. Anders, Moderated estimation of fold change and dispersion for RNA-seq data with DESeq2. *Genome biology* **15**, 550 (2014).
4. H. Li *et al.*, Anti-microRNA-378a enhances wound healing process by upregulating integrin beta-3 and vimentin. *Molecular therapy : the journal of the American Society of Gene Therapy* **22**, 1839 (Oct, 2014).
5. I. Tuszynska, M. Magnus, K. Jonak, W. Dawson, J. M. Bujnicki, NPDock: a web server for protein-nucleic acid docking. *Nucleic Acids Res* **43**, W425 (Jul 1, 2015).
6. M. J. Pietal, N. Szostak, K. M. Rother, J. M. Bujnicki, RNAmap2D - calculation, visualization and analysis of contact and distance maps for RNA and protein-RNA complex structures. *BMC Bioinformatics* **13**, 333 (2012).
7. A. Vangone, R. Spinelli, V. Scarano, L. Cavallo, R. Oliva, COCOMAPS: a web application to analyze and visualize contacts at the interface of biomolecular complexes. *Bioinformatics* **27**, 2915 (Oct 15, 2011).

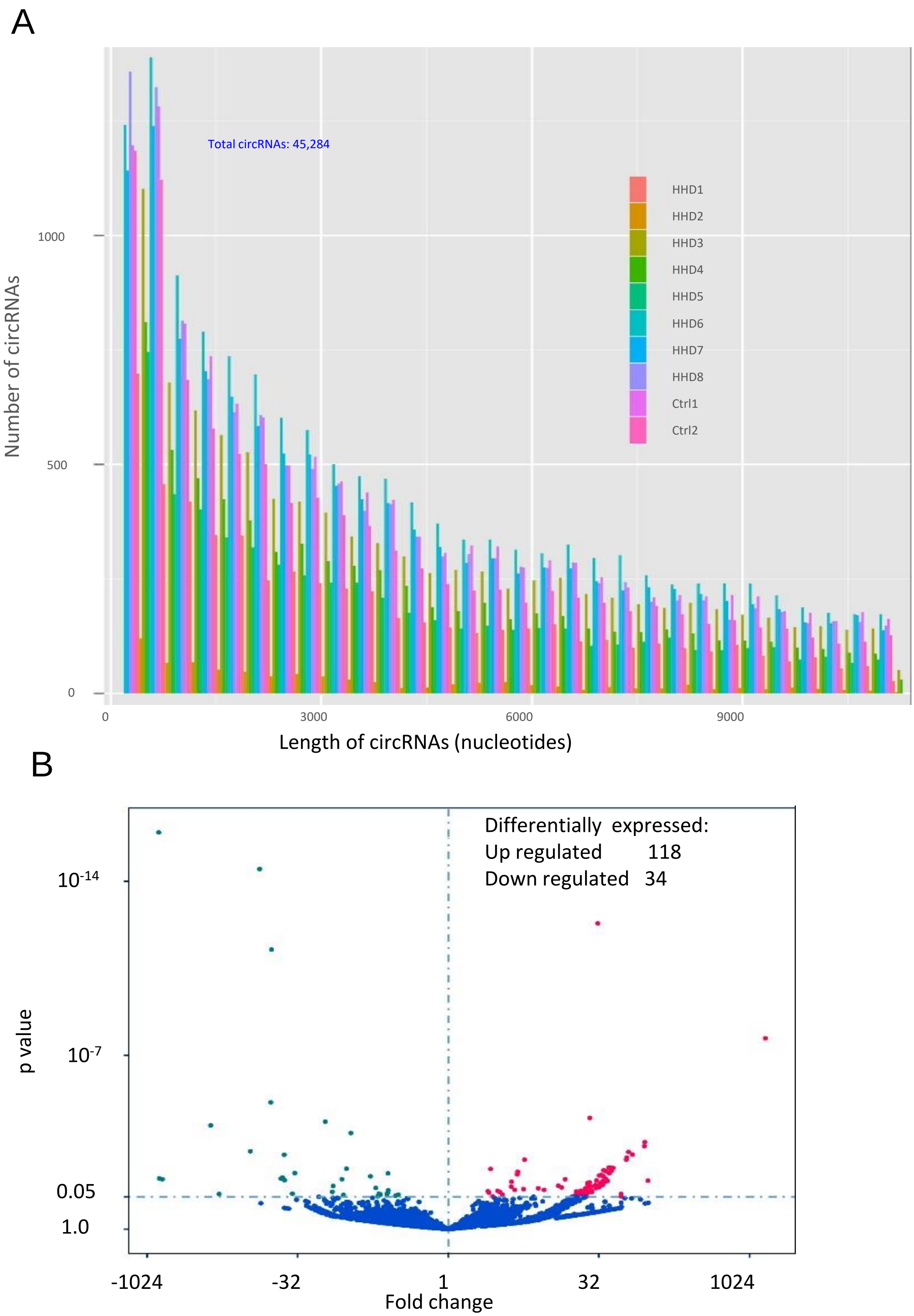

Supplementary Fig S1. Sequencing circRNAs in heart hypertrophy

(A) The length distributions of all circRNAs identified.

(B) Volcanoplot showing the up- and down-regulated circRNAs including their fold-change and p-values.

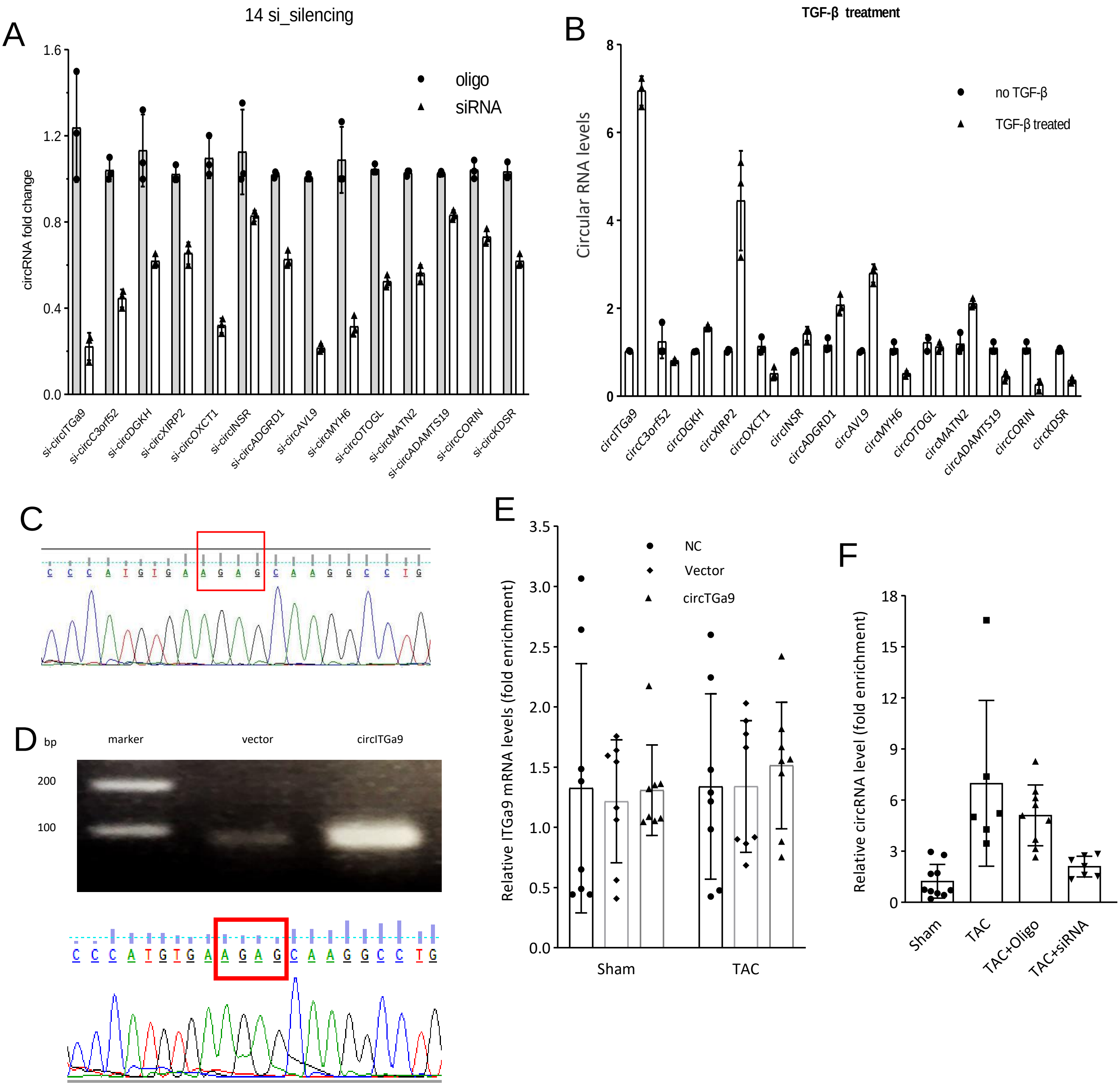

Fig S2. Silencing circITGa9

(A) HCF cells were transfected with siRNAs targeting the junction sequences of 14 circRNAs as indicated. Silencing circITGa9 showed the most fold-changes. n=4.

(B) HGF cells were treated with or without TGF-b, followed by measurement of 14 circular RNA levels. circITGa9 expression increased the most following TGF-b treatment. n=6. $^{*}p<0.05$.

(C) PCR was performed to confirm the presence of the circITGa9 junction sequence. Sanger-sequencing revealed the correct junction sequence of circITGa9.

(D) Upper, MCF cells were transfected with circITGa9 or a control vector followed by amplification of the junction sequence of circITGa9 by RT-PCR with the divergent primers. Lower, the PCR was subjected to DNA sequencing, confirming the correct junction sequence of circITGa9.

(E) Expression of ITGa9 linear mRNA was not affected by circITGa9 expression.

(F) Delivery of circItga9 siRNAs decreased circITGa9 levels.

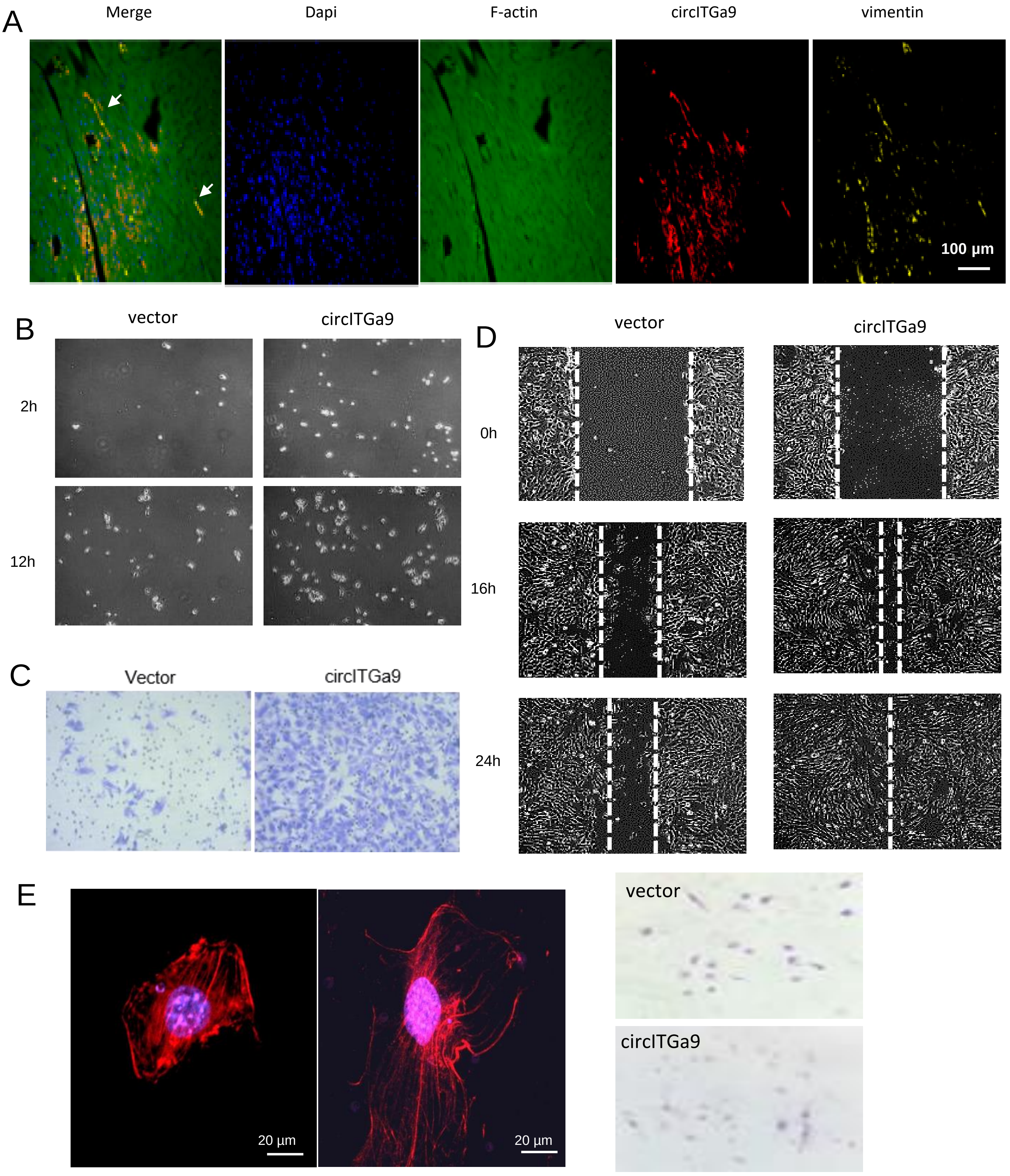

Fig S3. Co-localization of circITGa9 and cardiac fibroblasts. (A) Overexpression of circITGa9 increased adhesion (B) and migration (C, chambering migration and D wound healing migration). (E) Cardiac-fibroblast morphology change following circITGa9 overexpression (left, confocal microscopic examination; right, light microscopic examination).

A

B

C

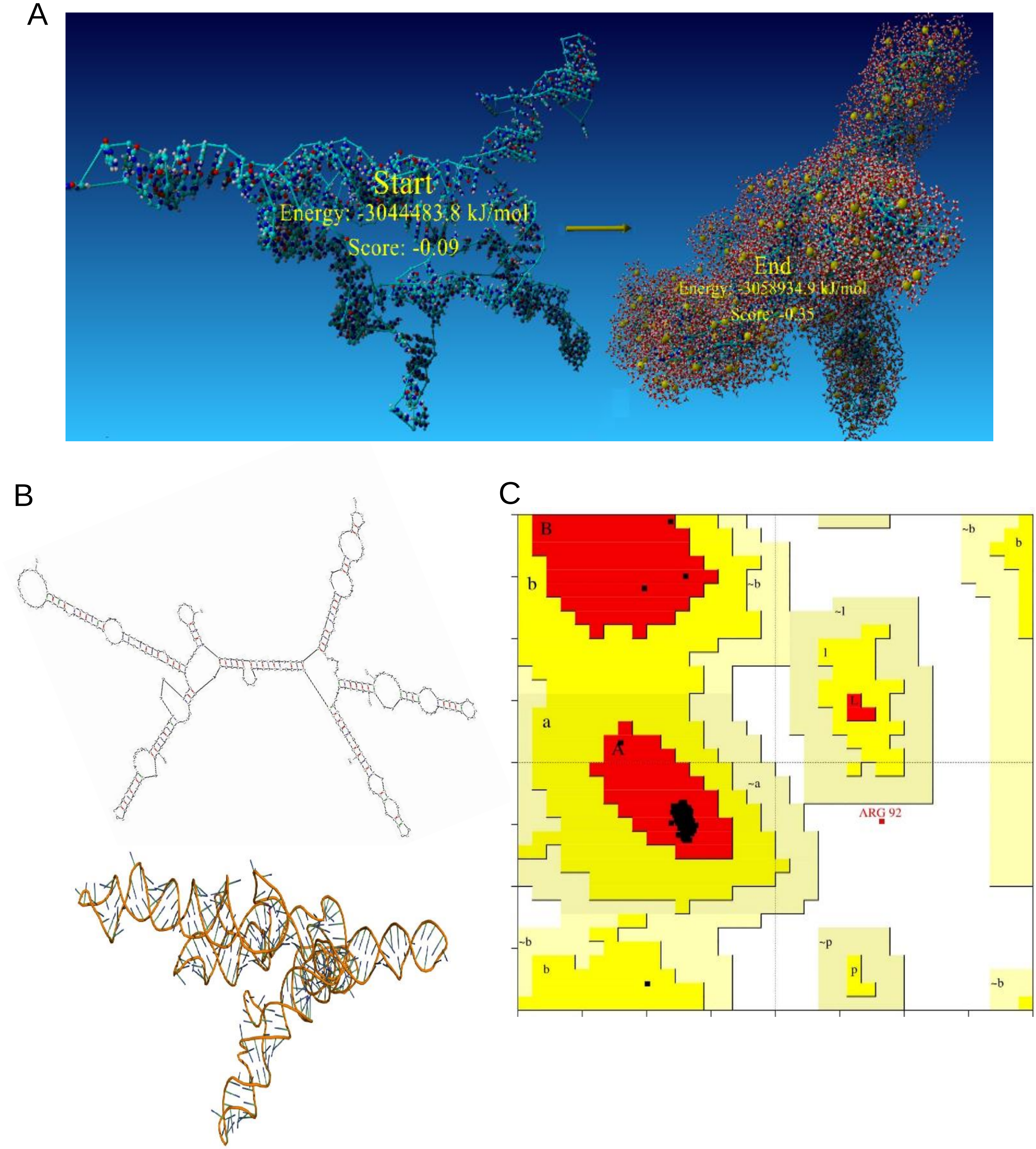

Fig S4. Computational analysis of circITGa9 and TPM3 structure.
(A) YASARA representation of energy minimized 3D circITGA9 RNA.
(B) 2D and 3D representation of circ-ITGa9 RNA. Upper, 2D RNA structure of circITGa9 was generated by Mfold. **Lower,** A 3D visualization of circITGA9 RNA produced in PyMol from estimates of 3D structure by RNA composer using the secondary structure delineated in dot bracket notation.
(C) The Ramachandran plot calculation of the psi/phi angle distribution of TPM3 model using PROCHECK validation server showing the stereochemical quality of the structure.

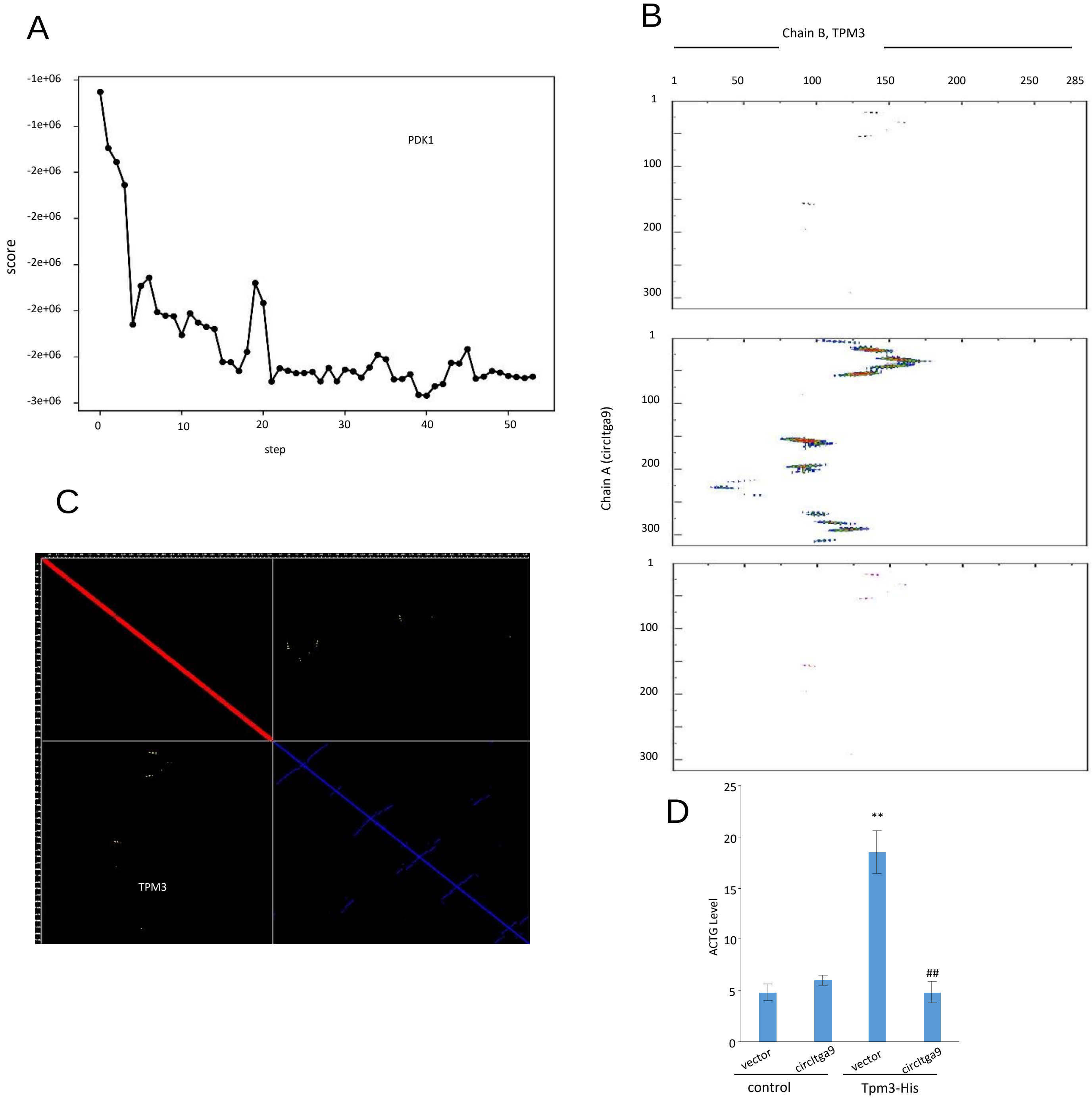

Fig S5. Computational analysis of circITGa9 interacting with TPM3.

(A) Refinement of the best docked circITGa9-TPM3 model showing MC score vs. steps of simulation.

(B) **Contact Maps of circITGa9-TPM3 complex. (a).** The “classical black and white map” presents a black dot at the crossover of two residues i and j, belonging to cir-ITGa9 and TPM3, if any atom of the two residues are closer than the cut-off distance (3.5 Å). (**b).** The "distance Range map" illustrating inter-molecular contacts at increasing distances, as colored dots (7 Å, 10 Å, 13 Å and 16 Å indicated in **Red**, **yellow**, **green**, and **blue** color) respectively. (**c).** The "property map" with each contact colored according to the physico-chemical nature of the two interacting residues, **yellow** = hydrophilic-hydropholic**, violet** = hydropohilic-hydrophilic, **green** = hydrophobic-hydrophobic.

(C) **Residue-level resolution contact map of CircITGa9-TPM3 complex.** CircITGa9 in complex with TPM3. In a contact map picture, protein contacts are displayed in Red, RNA contacts are blue, and the protein-RNA interface contacts are displayed in yellow. Distance range (3.5 Å).

(D) The Western blot of circITGa9 affecting TPM3-His binding to ACTG (Fig 6J) was repeated three times and the intensities were quantified.

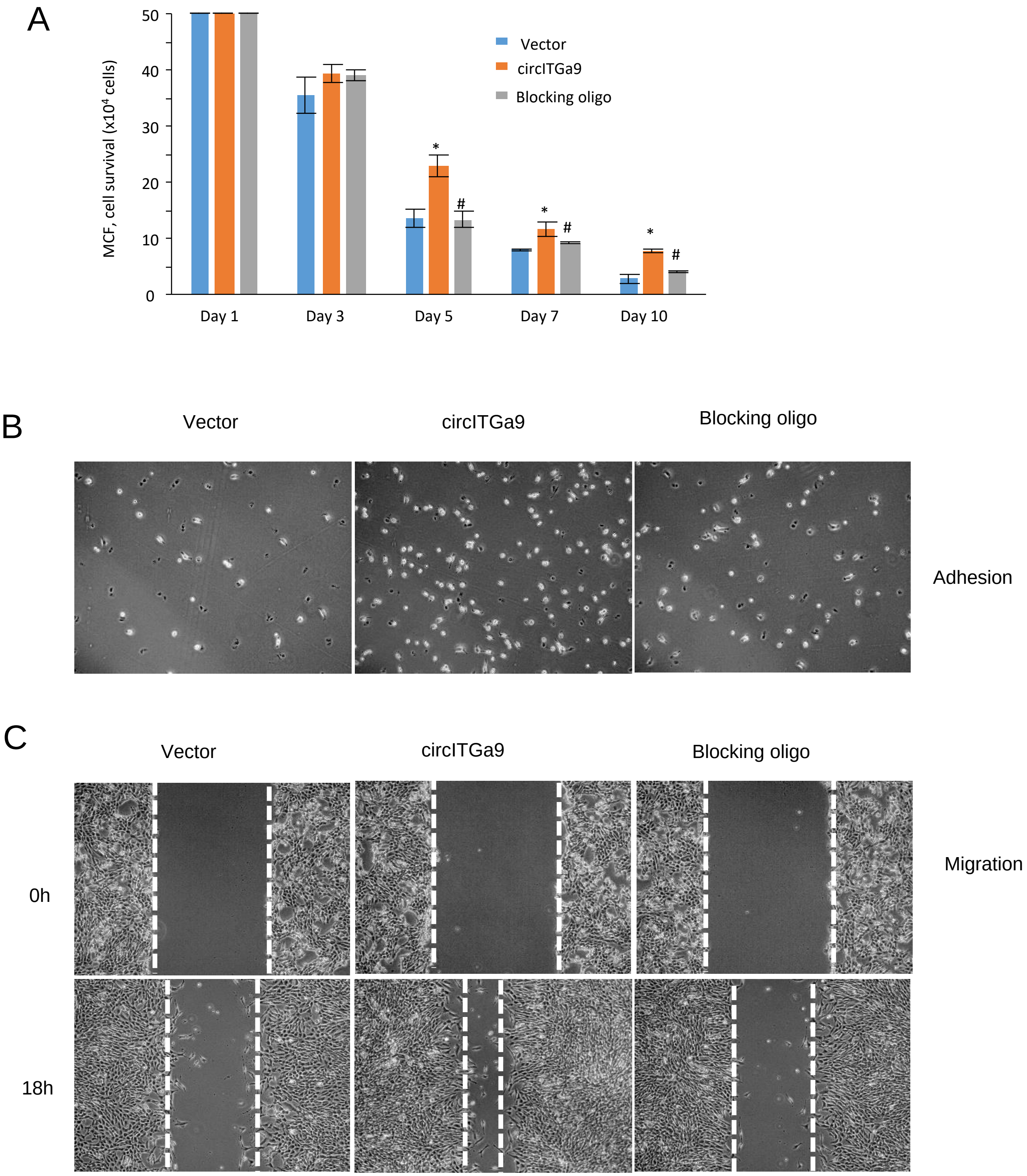

Fig S6. Blocking circITGa9-TPM3 interaction decreased survival (A), adhesion (B) and migration (C) of cardiac fibroblasts isolated from the mouse heart.

Supplementary Table S1. Differentially Expressed Unknown Circular RNAs

ID	HHD1	HHD2	HHD3	HHD4	HHD5	HHD6	HHD7	HHD8	Ctrl1	Ctrl2	D_readcount	C_readcount	log2FoldChange	p-value
hg38_circ_0000290	36.83132725	14.01515505	54.97910794	69.05505069	0	18.44519318	21.89237707	67.02502715	0	0	4.398371916	0	5.0149	0.0069563
hg38_circ_0000357	22.09879635	28.03031011	19.99240289	41.43303041	0	24.59359091	32.83856561	0	0	0	2.687136101	0	4.3362	0.032047
hg38_circ_0000484	33.14819453	18.68687341	19.99240289	41.43303041	0	18.44519318	43.78475415	0	0	0	2.791756474	0	4.3861	0.030168
hg38_circ_0001430	22.09879635	9.343436703	0	96.67707096	0	79.92917046	21.89237707	26.81001086	0	0	4.060707947	0	4.8837	0.016964
hg38_circ_0002110	36.83132725	84.09093033	89.96581299	0	0	215.1939205	43.78475415	80.43003257	13.4617283	0	8.842412091	0.931490168	3.181	0.026438
hg38_circ_0002171	106.810849	65.40405692	49.98100722	41.43303041	0	0	43.78475415	0	0	0	4.922999111	0	5.2291	0.010163
hg38_circ_0002296	18.41566363	60.73233857	64.97530938	55.24404055	0	0	0	0	0	0	3.146385798	0	4.5729	0.044337
hg38_circ_0002298	58.93012361	18.68687341	94.96391371	138.1101014	93.98496241	12.29679546	0	26.81001086	0	0	6.086134729	0	5.4739	0.0034839
hg38_circ_0002704	22.09879635	32.70202846	39.98480577	0	0	0	21.89237707	53.62002172	0	0	2.65168033	0	4.3205	0.046611
hg38_circ_0003071	44.1975927	93.43436703	29.98860433	69.05505069	0	0	0	67.02502715	0	0	4.723097797	0	5.1366	0.011762
hg38_circ_0003340	128.9096454	28.03031011	9.996201443	69.05505069	0	24.59359091	65.67713122	227.8850923	8.97448554	0	8.57350364	0.620993445	3.653	0.018029
hg38_circ_0004879	55.24699088	32.70202846	29.98860433	41.43303041	0	12.29679546	32.83856561	0	0	0	3.26515319	0	4.6406	0.019838
hg38_circ_0004898	7.366265451	9.343436703	44.9829065	55.24404055	0	0	0	120.6450489	0	0	3.598872813	0	4.6903	0.032141
hg38_circ_0005451	14.7325309	0	9.996201443	0	0	12.29679546	21.89237707	0	85.2576126	95.73042313	0.953394278	12.12533236	-3.5297	0.0098382
hg38_circ_0006667	44.1975927	56.06062022	24.99050361	69.05505069	939.8496241	12.29679546	21.89237707	80.43003257	8.97448554	0	10.7006421	0.620993445	3.8817	0.0098662
hg38_circ_0006768	11.04939818	28.03031011	0	0	939.8496241	24.59359091	32.83856561	0	0	0	7.45470869	0	5.735	0.047829
hg38_circ_0006783	29.4650618	28.03031011	14.99430217	0	516.9172932	36.89038637	0	0	0	0	5.034682117	0	5.123	0.01428
hg38_circ_0006951	29.4650618	18.68687341	49.98100722	41.43303041	0	67.632375	0	0	139.104526	123.0819726	3.346345391	17.63012007	-2.3006	0.037523
hg38_circ_0007593	55.24699088	18.68687341	29.98860433	96.67707096	187.9699248	43.03878409	0	40.21501629	0	0	5.654543101	0	5.3333	0.0033361
hg38_circ_0007794	14.7325309	46.71718351	19.99240289	0	845.8646617	0	54.73094269	0	0	0	7.472651111	0	5.7399	0.038782
hg38_circ_0008321	0	0	0	0	0	0	0	0	188.464196	369.2459178	0	37.05502737	-7.6063	0.038793
hg38_circ_0008324	125.2265127	70.07577527	39.98480577	124.2990912	0	288.9746932	0	0	785.267485	1490.659446	10.55160364	151.283	-3.8218	0.018422
hg38_circ_0008325	0	0	0	0	0	1764.590148	0	0	1978.87406	0	29.51120078	136.9290546	-9.4923	0.0098742
hg38_circ_0008327	176.7903708	186.8687341	139.9468202	511.0073751	0	891.5176705	766.2331976	536.2002172	2508.36871	4670.277071	50.69529814	477.3023847	-3.2336	0.00013627
hg38_circ_0008335	40.51445998	60.73233857	19.99240289	82.86606082	0	811.5885	65.67713122	93.835038	2719.26912	7562.703427	19.1920709	680.00669	-6.2618	1.28E-17
hg38_circ_0008336	51.56385816	74.74749362	0	110.4880811	0	1438.725068	120.4080739	93.835038	4294.29133	6557.533984	31.04462371	723.619146	-5.8897	7.91E-06
hg38_circ_0008342	14.7325309	23.35859176	14.99430217	0	0	215.1939205	0	0	780.780242	895.763245	4.45215658	112.2830152	-5.8664	5.57E-12
hg38_circ_0008352	0	0	0	0	0	0	0	0	430.775306	1798.364377	0	146.7655632	-9.5922	0.0091237
hg38_circ_0008353	0	18.68687341	0	0	0	245.9359091	0	40.21501629	641.675716	1853.067476	4.997257434	164.9165632	-6.5659	0.00073659
hg38_circ_0008361	0	0	0	0	0	0	0	0	919.884768	1306.036487	0	148.5908192	-9.6097	1.18E-20
hg38_circ_0008372	14.7325309	28.03031011	0	0	0	399.6458523	0	0	951.295467	6037.854544	7.370812535	458.5013739	-7.8856	6.72E-05
hg38_circ_0008376	0	0	0	0	0	0	0	0	49.3596705	75.21676103	0	8.307238304	-5.448	0.010222
hg38_circ_0008401	40.51445998	51.38890187	54.97910794	0	0	0	0	53.62002172	0	0	3.148861079	0	4.5853	0.041694
hg38_circ_0008403	11.04939818	28.03031011	24.99050361	27.62202027	0	30.74198864	109.4618854	67.02502715	0	0	4.659716901	0	5.0784	0.0066615
hg38_circ_0008412	40.51445998	28.03031011	109.9582159	55.24404055	0	12.29679546	21.89237707	93.835038	0	0	5.639878961	0	5.3928	0.0034034

hg38_circ_0008576	7.366265451	0	0	0	0	0	0	0	71.7958843	82.0546484	0.122210463	10.30442868	-5.0946	0.0055842
hg38_circ_0008641	88.39518541	60.73233857	59.97720866	0	0	0	21.89237707	40.21501629	0	0	4.317772347	0	5.0507	0.014594
hg38_circ_0008778	110.4939818	98.10608538	84.96771227	0	0	49.18718182	98.51569683	80.43003257	197.438682	239.3260578	8.288003884	29.22659238	-1.7479	0.045295
hg38_circ_0008900	25.78192908	28.03031011	24.99050361	27.62202027	0	24.59359091	54.73094269	26.81001086	0	0	3.353787266	0	4.6457	0.013665
hg38_circ_0009264	0	0	0	0	0	12.29679546	0	0	130.13004	75.21676103	0.205652967	13.89617931	-5.444	0.0010205
hg38_circ_0010585	88.39518541	112.1212404	94.96391371	151.9211115	0	98.37436364	43.78475415	201.0750814	318.594237	430.7869041	12.34985618	50.06179316	-1.9999	0.0057326
hg38_circ_0010795	18.41566363	0	24.99050361	0	234.962406	12.29679546	43.78475415	0	0	0	3.073380504	0	4.4088	0.044485
hg38_circ_0011145	3753.112247	3541.16251	1774.325756	1408.723034	422.9323308	110.6711591	996.1031569	924.9453746	0	0	201.7334528	0	10.529	2.08E-08
hg38_circ_0011200	147.325309	186.8687341	139.9468202	110.4880811	0	0	120.4080739	80.43003257	0	0	12.38550627	0	6.5291	0.00031463
hg38_circ_0011716	14.7325309	9.343436703	29.98860433	0	0	43.03878409	21.89237707	40.21501629	0	0	2.524057281	0	4.2509	0.040998
hg38_circ_0011717	29.4650618	0	0	0	0	30.74198864	43.78475415	107.2400434	0	0	3.260618193	0	4.5633	0.047988
hg38_circ_0012001	375.679538	485.8587086	369.8594534	538.6293954	2772.556391	116.8195568	1587.197338	1005.375407	107.693827	314.5428189	87.02162314	27.90843228	1.6384	0.028966
hg38_circ_0012289	11.04939818	0	0	0	0	18.44519318	0	0	85.2576126	47.86521156	0.491795145	9.012385046	-3.8465	0.031684
hg38_circ_0012392	18.41566363	23.35859176	0	0	0	36.89038637	87.5695083	26.81001086	0	0	3.057603066	0	4.4947	0.038073
hg38_circ_0014084	7.366265451	18.68687341	19.99240289	82.86606082	0	12.29679546	32.83856561	0	0	0	2.721081202	0	4.2903	0.040771
hg38_circ_0014370	11.04939818	32.70202846	0	0	140.9774436	24.59359091	21.89237707	26.81001086	0	0	2.73252202	0	4.2665	0.041969
hg38_circ_0014555	36.83132725	9.343436703	29.98860433	55.24404055	0	0	0	53.62002172	0	0	2.865990016	0	4.3927	0.043772
hg38_circ_0015207	55.24699088	18.68687341	34.98670505	0	0	24.59359091	0	53.62002172	0	0	2.968250179	0	4.5078	0.033614
hg38_circ_0015231	51.56385816	14.01515505	9.996201443	0	0	18.44519318	0	174.2650706	0	0	4.097932497	0	4.9021	0.022571
hg38_circ_0015472	1399.590436	1406.187224	1749.335253	1602.077176	1738.721805	1082.118	1204.080739	1930.320782	507.058433	471.8142283	174.0170546	65.77089606	1.406	0.0038043
hg38_circ_0015569	36.83132725	42.04546516	34.98670505	41.43303041	0	12.29679546	21.89237707	26.81001086	0	0	3.40819127	0	4.6922	0.013299
hg38_circ_0015718	250.4530253	70.07577527	289.8898419	69.05505069	1080.827068	184.4519318	175.1390166	241.2900977	62.8213988	82.0546484	27.10686582	9.683435231	1.4765	0.048566
hg38_circ_0015928	22.09879635	32.70202846	34.98670505	0	0	473.426625	0	0	336.543208	410.273242	9.360525298	49.96965977	-4.0815	4.74E-05
hg38_circ_0016025	22.09879635	51.38890187	14.99430217	0	0	24.59359091	0	93.835038	0	0	3.203947057	0	4.5795	0.031048
hg38_circ_0016043	69.97952178	60.73233857	14.99430217	0	0	49.18718182	43.78475415	0	0	0	3.869398448	0	4.9009	0.017359
hg38_circ_0016067	29.4650618	9.343436703	49.98100722	27.62202027	0	0	43.78475415	67.02502715	0	0	3.52573139	0	4.6948	0.019991
hg38_circ_0016325	33.14819453	28.03031011	44.9829065	41.43303041	0	0	21.89237707	0	0	0	2.687319499	0	4.3492	0.044904
hg38_circ_0016424	7.366265451	9.343436703	19.99240289	55.24404055	657.8947368	12.29679546	0	0	0	0	5.76905759	0	5.287	0.011503
hg38_circ_0016462	40.51445998	0	24.99050361	55.24404055	0	0	32.83856561	26.81001086	0	0	2.821906785	0	4.367	0.047089
hg38_circ_0016603	7.366265451	23.35859176	29.98860433	41.43303041	0	12.29679546	54.73094269	53.62002172	0	0	3.452050417	0	4.6401	0.016083
hg38_circ_0016928	33.14819453	32.70202846	34.98670505	27.62202027	0	18.44519318	32.83856561	26.81001086	0	0	3.263518444	0	4.6313	0.014187
hg38_circ_0016983	25.78192908	0	0	27.62202027	0	61.48397728	32.83856561	40.21501629	376.928393	75.21676103	2.981200238	30.97349905	-3.3781	0.0037105
hg38_circ_0017433	40.51445998	60.73233857	24.99050361	27.62202027	0	18.44519318	120.4080739	26.81001086	0	0	5.041957573	0	5.2303	0.0046633
hg38_circ_0018575	110.4939818	121.4646771	0	165.7321216	0	166.0067386	284.600902	0	0	0	13.51789738	0	6.6305	0.011258
hg38_circ_0019311	0	9.343436703	0	0	0	307.4198864	0	0	358.979422	232.4881705	5.288880904	39.95976763	-5.5505	0.0092952
hg38_circ_0020161	195.2060344	163.5101423	139.9468202	345.2752534	93.98496241	61.48397728	65.67713122	549.6052226	35.8979422	68.37887366	24.0226264	6.931041377	1.8006	0.040779
hg38_circ_0020181	143.6421763	88.76264868	54.97910794	138.1101014	657.8947368	73.78077273	32.83856561	201.0750814	31.4106994	20.5136621	15.59589259	3.507597337	2.0927	0.019571

hg38_circ_0020269	11.04939818	18.68687341	19.99240289	27.62202027	0	30.74198864	0	40.21501629	0	0	2.322585903	0	4.1087	0.04988
hg38_circ_0021276	36.83132725	60.73233857	34.98670505	55.24404055	281.9548872	0	0	0	0	0	4.742784092	0	5.0806	0.013269
hg38_circ_0021284	11.04939818	14.01515505	14.99430217	41.43303041	610.9022556	0	21.89237707	0	0	0	5.456048786	0	5.2009	0.012455
hg38_circ_0021425	18.41566363	23.35859176	19.99240289	138.1101014	0	12.29679546	0	0	0	0	3.305212556	0	4.573	0.034949
hg38_circ_0021510	36.83132725	0	19.99240289	41.43303041	0	43.03878409	32.83856561	0	0	0	2.797366301	0	4.3862	0.043341
hg38_circ_0021663	51.56385816	60.73233857	44.9829065	0	0	55.33557955	0	0	0	0	3.459781054	0	4.7494	0.030661
hg38_circ_0021668	128.9096454	224.2424809	224.9145325	248.5981825	610.9022556	172.1551364	131.3542624	254.6951032	49.3596705	102.5683105	25.57504773	10.08606534	1.3516	0.034221
hg38_circ_0021761	7.366265451	14.01515505	24.99050361	0	328.9473684	18.44519318	0	0	0	0	3.117710155	0	4.4029	0.046589
hg38_circ_0021918	44.1975927	42.04546516	19.99240289	0	0	12.29679546	0	67.02502715	0	0	2.904617964	0	4.4641	0.037374
hg38_circ_0022137	36.83132725	23.35859176	39.98480577	0	0	0	43.78475415	53.62002172	0	0	3.091920305	0	4.5352	0.032108
hg38_circ_0022551	36.83132725	23.35859176	19.99240289	69.05505069	0	18.44519318	0	0	0	0	2.660938998	0	4.3164	0.045902
hg38_circ_0023237	33.14819453	32.70202846	79.96961155	0	0	24.59359091	32.83856561	93.835038	0	0	4.646852927	0	5.126	0.0072784
hg38_circ_0023577	0	28.03031011	29.98860433	55.24404055	0	24.59359091	43.78475415	0	0	0	2.86266245	0	4.3937	0.042817
hg38_circ_0024492	0	14.01515505	24.99050361	55.24404055	0	104.5227614	21.89237707	0	0	0	3.554723568	0	4.7218	0.030115
hg38_circ_0024608	7.366265451	23.35859176	9.996201443	0	0	24.59359091	43.78475415	53.62002172	0	0	2.534550558	0	4.2164	0.044399
hg38_circ_0024720	29.4650618	158.8384239	134.9487195	55.24404055	0	0	197.0313937	227.8850923	0	0	12.42718765	0	6.5117	0.00045897
hg38_circ_0024722	151.0084417	210.2273258	319.8784462	179.5431318	375.9398496	36.89038637	383.1165988	294.9101194	13.4617283	0	26.98732673	0.931490168	4.7003	3.35E-05
hg38_circ_0024783	14.7325309	28.03031011	14.99430217	41.43303041	0	36.89038637	0	40.21501629	0	0	2.764627279	0	4.3573	0.032429
hg38_circ_0025066	33.14819453	88.76264868	0	55.24404055	0	24.59359091	109.4618854	40.21501629	0	0	5.511106385	0	5.3438	0.005712
hg38_circ_0025881	0	0	0	0	0	0	0	0	67.3086415	54.70309893	0	8.215104915	-5.4343	0.010732
hg38_circ_0026650	33.14819453	32.70202846	24.99050361	0	0	24.59359091	0	80.43003257	0	0	3.055732363	0	4.5226	0.031825
hg38_circ_0026651	257.8192908	313.0051295	139.9468202	262.4091926	0	166.0067386	175.1390166	321.7201303	62.8213988	54.70309893	25.69571898	7.904608193	1.7135	0.032391
hg38_circ_0027148	567.2024397	541.9193288	769.7075111	593.8734359	5780.075188	86.07756819	218.9237707	1032.185418	85.2576126	205.136621	95.62945594	19.24064052	2.31	0.0050136
hg38_circ_0028297	36.83132725	28.03031011	64.97530938	41.43303041	0	55.33557955	43.78475415	26.81001086	0	0	4.729851171	0	5.1631	0.0043159
hg38_circ_0028322	18.41566363	37.37374681	29.98860433	41.43303041	0	0	0	53.62002172	0	0	2.792608796	0	4.3673	0.043062
hg38_circ_0028496	14.7325309	37.37374681	14.99430217	55.24404055	0	0	0	67.02502715	0	0	2.898473436	0	4.3935	0.04337
hg38_circ_0028591	18.41566363	0	29.98860433	55.24404055	0	24.59359091	0	53.62002172	0	0	2.824213067	0	4.3583	0.045199
hg38_circ_0028615	117.8602472	46.71718351	59.97720866	69.05505069	0	18.44519318	186.0852051	40.21501629	0	0	8.52170307	0	5.9835	0.00078495
hg38_circ_0028988	364.6301398	359.7223131	499.8100722	331.4642433	0	184.4519318	372.1704103	174.2650706	89.7448554	34.18943683	36.25507089	8.433468249	2.1031	0.012367
hg38_circ_0029662	33.14819453	28.03031011	29.98860433	69.05505069	0	12.29679546	0	0	0	0	2.730730256	0	4.3565	0.043453
hg38_circ_0029669	7.366265451	32.70202846	24.99050361	165.7321216	0	79.92917046	0	0	170.515225	198.2987336	4.901567691	24.69537149	-2.3442	0.048038
hg38_circ_0030203	77.34578723	28.03031011	44.9829065	27.62202027	0	49.18718182	76.62331976	0	76.2831271	246.1639452	4.891108342	21.28788763	-2.0337	0.039272
hg38_circ_0030354	14.7325309	28.03031011	14.99430217	0	0	18.44519318	65.67713122	67.02502715	0	0	3.247422527	0	4.5718	0.02306
hg38_circ_0030357	36.83132725	23.35859176	24.99050361	0	0	79.92917046	76.62331976	0	0	0	3.918365152	0	4.8911	0.017344
hg38_circ_0030598	81.02891996	200.8838891	169.9354245	124.2990912	0	36.89038637	164.1928281	80.43003257	17.9489711	27.35154947	13.50101671	3.020813929	2.2057	0.026374
hg38_circ_0030600	1012.861499	1452.904407	1564.405526	1436.345054	2396.616541	393.4974546	788.1255747	1675.625679	40.3851849	27.35154947	145.2135828	4.573297541	4.9661	4.93E-13
hg38_circ_0031448	18.41566363	18.68687341	0	27.62202027	0	61.48397728	76.62331976	26.81001086	0	0	3.644493229	0	4.7416	0.019406

hg38_circ_0032125	18.41566363	32.70202846	34.98670505	0	845.8646617	24.59359091	54.73094269	53.62002172	0	0	8.74909029	0	5.9103	0.0016013
hg38_circ_0032354	125.2265127	46.71718351	69.9734101	96.67707096	422.9323308	0	0	107.2400434	0	0	9.63560177	0	6.1155	0.00099996
hg38_circ_0032802	482.490387	331.692003	394.849957	469.5743447	469.924812	36.89038637	186.0852051	254.6951032	35.8979422	61.5409863	36.93641831	6.486334617	2.5321	0.0016077
hg38_circ_0033081	73.66265451	46.71718351	19.99240289	0	0	67.632375	0	80.43003257	224.362138	157.2714094	4.589038077	25.7530916	-2.4062	0.022091
hg38_circ_0033082	73.66265451	60.73233857	24.99050361	0	0	61.48397728	0	26.81001086	197.438682	109.4061979	4.0020728	20.77716395	-2.2501	0.041068
hg38_circ_0033095	198.8891672	144.8232689	44.9829065	124.2990912	0	276.6778977	0	40.21501629	547.443618	690.626624	13.41756605	82.79598287	-2.5806	0.0075155
hg38_circ_0033988	220.9879635	200.8838891	144.9449209	207.1651521	0	676.32375	109.4618854	268.1001086	659.624687	738.4918356	29.27088193	93.67134826	-1.6585	0.04094
hg38_circ_0034015	44.1975927	0	0	0	0	270.5295	0	0	489.109462	61.5409863	5.257628054	37.84650359	-5.1741	0.037712
hg38_circ_0034294	25.78192908	14.01515505	19.99240289	27.62202027	0	24.59359091	21.89237707	26.81001086	0	0	2.537416211	0	4.2486	0.030818
hg38_circ_0034628	254.1361581	191.5404524	134.9487195	372.8972737	0	202.897125	197.0313937	201.0750814	40.3851849	34.18943683	24.51368669	5.018004301	2.2934	0.0063084
hg38_circ_0034663	0	32.70202846	14.99430217	55.24404055	0	0	32.83856561	67.02502715	0	0	3.09533781	0	4.4682	0.041509
hg38_circ_0034669	33.14819453	23.35859176	24.99050361	0	0	18.44519318	43.78475415	26.81001086	0	0	2.706654516	0	4.3667	0.031068
hg38_circ_0034774	29.4650618	28.03031011	24.99050361	27.62202027	0	30.74198864	0	26.81001086	0	0	2.659279544	0	4.3439	0.032522
hg38_circ_0035378	25.78192908	70.07577527	64.97530938	96.67707096	0	0	65.67713122	0	0	0	5.07785603	0	5.2331	0.0095303
hg38_circ_0035648	29.4650618	23.35859176	0	0	0	36.89038637	43.78475415	67.02502715	0	0	3.143251851	0	4.5376	0.032999
hg38_circ_0035978	11.04939818	28.03031011	14.99430217	27.62202027	0	36.89038637	0	53.62002172	0	0	2.689358354	0	4.316	0.036147
hg38_circ_0036343	29.4650618	28.03031011	34.98670505	27.62202027	0	18.44519318	0	40.21501629	0	0	2.809932734	0	4.4136	0.028702
hg38_circ_0036543	25.78192908	23.35859176	14.99430217	0	0	49.18718182	54.73094269	26.81001086	0	0	3.110319748	0	4.5527	0.023008
hg38_circ_0036568	25.78192908	28.03031011	24.99050361	0	0	79.92917046	32.83856561	0	0	0	3.122076271	0	4.5857	0.029913
hg38_circ_0037547	47.88072543	70.07577527	39.98480577	0	0	98.37436364	0	0	0	0	4.186045629	0	5.0102	0.020608
hg38_circ_0037549	51.56385816	37.37374681	69.9734101	69.05505069	0	0	0	0	0	0	3.617938611	0	4.7709	0.031685
hg38_circ_0038387	11.04939818	14.01515505	14.99430217	0	0	79.92917046	76.62331976	0	0	0	3.183127196	0	4.5753	0.0351
hg38_circ_0039368	14.7325309	18.68687341	0	55.24404055	0	18.44519318	98.51569683	0	0	0	3.235305773	0	4.5455	0.036834
hg38_circ_0039497	77.34578723	163.5101423	84.96771227	96.67707096	0	24.59359091	0	67.02502715	0	0	8.091772092	0	5.9282	0.0013336
hg38_circ_0039548	162.0578399	154.1667056	184.9297267	179.5431318	1409.774436	43.03878409	229.8699593	174.2650706	31.4106994	47.86521156	26.55058052	5.286424375	2.304	0.0049393
hg38_circ_0039552	47.88072543	42.04546516	44.9829065	0	0	18.44519318	21.89237707	80.43003257	0	0	4.008148525	0	4.9242	0.011028
hg38_circ_0039558	69.97952178	70.07577527	79.96961155	110.4880811	0	49.18718182	76.62331976	67.02502715	8.97448554	13.67577473	8.237668452	1.510406964	2.498	0.024665
hg38_circ_0039640	81.02891996	65.40405692	39.98480577	82.86606082	0	30.74198864	0	40.21501629	179.489711	157.2714094	5.383359108	22.64812437	-2.0061	0.033139
hg38_circ_0039926	25.78192908	0	0	0	0	36.89038637	21.89237707	134.0500543	0	0	3.351686764	0	4.5998	0.047994
hg38_circ_0040200	0	23.35859176	0	41.43303041	0	0	0	0	98.7193409	61.5409863	1.000467533	10.83328873	-3.4769	0.042174
hg38_circ_0041346	77.34578723	42.04546516	34.98670505	41.43303041	0	12.29679546	87.5695083	0	0	0	4.717753047	0	5.1539	0.0075545
hg38_circ_0041573	198.8891672	196.2121708	149.9430217	220.9761622	422.9323308	196.7487273	240.8161478	241.2900977	58.334156	95.73042313	25.42409451	10.26235203	1.3213	0.030132
hg38_circ_0041674	0	0	0	0	0	0	0	0	53.8469132	75.21676103	0	8.617735027	-5.5013	0.008469
hg38_circ_0041718	36.83132725	79.41921197	59.97720866	69.05505069	0	67.632375	43.78475415	0	139.104526	218.8123957	5.69542121	23.85601471	-1.9907	0.027606
hg38_circ_0041754	788.1904032	126.1363955	364.8613527	690.5505069	0	61.48397728	87.5695083	93.835038	0	41.0273242	35.20907434	2.668240558	3.7629	0.021038
hg38_circ_0041994	29.4650618	23.35859176	19.99240289	41.43303041	0	18.44519318	43.78475415	0	0	0	2.804429605	0	4.3927	0.029405
hg38_circ_0042157	18.41566363	28.03031011	0	27.62202027	0	12.29679546	43.78475415	26.81001086	0	0	2.454374424	0	4.1672	0.048595

hg38_circ_0042430	125.2265127	51.38890187	49.98100722	96.67707096	0	92.22596591	32.83856561	40.21501629	13.4617283	0	7.809085144	0.931490168	2.9899	0.022712
hg38_circ_0042445	11.04939818	9.343436703	0	0	281.9548872	18.44519318	0	107.2400434	0	0	3.980950211	0	4.7704	0.029802
hg38_circ_0042667	29.4650618	14.01515505	34.98670505	69.05505069	0	0	0	26.81001086	0	0	2.715332367	0	4.3192	0.048482
hg38_circ_0043481	29.4650618	9.343436703	49.98100722	69.05505069	0	18.44519318	0	0	0	0	2.797227276	0	4.3879	0.043554
hg38_circ_0043582	77.34578723	56.06062022	54.97910794	124.2990912	0	12.29679546	0	40.21501629	0	0	5.737710334	0	5.4163	0.0045587
hg38_circ_0043995	0	37.37374681	14.99430217	0	516.9172932	18.44519318	43.78475415	0	0	0	5.07166909	0	5.1247	0.016743

Supplementary Table S2. Junction sequences of circRNSs

```
>hg38_circ_0000290_junction_seq
CAATATCATCTCAGTTCTCATGCTGACATTGGTTCTGACCAACAGGAATATACAGAAGACTATGAGCAACCCAG
GGGCAAGGGGAGCTTTCCAGCCATGATCACACCTGCTTATCAAAGGGCCAAGAAAGCCAACCAGCTGGCCAGCC
AAAGAGCCTATTGGACTGGATATGGGGAAGGGAATGCTTGGTGCCCAGGAGCTCTGCCAGACCCCGAAATTGTA
AGGATGGTTGAGGCTCGAAAGTCTCTTGGTGAGGTAAGCACCCTAGAAATTGCTTTAGGAATTGGTTTAGCAAT
TACT
>hg38_circ_0000357_junction_seq
GAATATAACAGGTATGATACTCTCCCTTGTTTTGTTTTATTCTGCAGGTTACTCATTTTTGGATCCTATGACAG
AGAGGCAAATGTTCATTGCACACTTGAGTTAAGCAGTAGTGTTTGGGAAGAAAAACAGAGGAGTTCTATTAAGA
CGGTTTAAGCCGTTTTTTCCATACCAGACCTTGCAAGGATTTGAAGAAGATGAAGAGCATATCCATATACAACA
ATGGGCACTTACTGAAGGCCGTCTTAAAGTTACGTTGTTAGAATGTAGCAGGTATGTTCTTTGTTTTGTCATTA
CTAA
>hg38_circ_0000484_junction_seq
CTGATTTCCTTTCCAATTTCTTTTTCTCCTGGCTCAGAGGACTTTATCAGCTCAGACTCCAAGGTCCGCGCAGC
CACCCGGGAACAGTCAGAATATAAAAAGGAAGCAGCAGGACACGCCCGGAAGCCCTGACCACAGAGACGCGTCC
AGTCACCTCTGACAAAATTCTGGGGACGCTGGGAACACTGAATCAACATGGGCAATGAGAACAGCACCTCGGAC
AACCAGGTGGGTGTCAGGAAGCTTCTCTCTCGAGCTACGTGGTGCTCTTGGCAGACCTTGACTAGGTTCTTTTT
ACAG
>hg38_circ_0001430_junction_seq
ACCAGGGTCTTCCCCTTCATCTCTGTACTATGAAGAACCTCTGGGGCAACCTCCCCGGTTCACTCAAAAGTTAC
GGAGCAGAGAAGTTCCAGAAGGAACTCGAGTACAGTTGGATTGCATAGTGGTAGGAATTCCACCACCTCAAGTA
AGCATGCAAGACGACAGCATAGAAGCTTCTACTTCCATATCTCAGCTTCTAAGAGAGAGCTATTTAGCTGAAAC
CAGACATCGGGGAAACAATGAGAGGAGTCGAGCGGAGCCCTCCTCCAACCCTTGCCATTTCGGCAGTCCTTCTG
GGGC
>hg38_circ_0002110_junction_seq
CCTGGCCAATGACAGTGTTTTCCTCATTTCAGGGGATCCCAACAAGCCCTCAGGATTCAGAAGTGTTAAAGCTC
CTGTCACTAAAGTGGCTGCGTCGATTGGAAATGCTCAGAAGTTGCCTATGTGTGACAAATGTGGCACTGGGATT
GTCTTAGACCATGCTCAGCCTCCAAGCAGCCTTGTCATCGACAAAGAATCTGAAGTTTACAAGATGCTTCAGGA
GAAACAGGAGTTGAATGAGCCCCCGAAACAGTCCACGTCTTTCTTGGTTTTGCAGGAAATCCTGGAGTCTGAAG
AAAA
>hg38_circ_0002171_junction_seq
TCCTCAGGCTTTGCTGCCAAAACTCACTTTATGGTAATTCTTTACAGATCGTATTAAACTTCACATCCATGGAT
TTGTTTAAAAGCCGACTGTGCTGGTATGATTACGTGGAGGTCCGGGATGGTTACTGGAGAAAAGCCCCCCTTTT
GGAGGAGTTTTCTTAGACACCATCCTTCCCCGTCAAGATGACAATGGCGTCAGGCCAACCATTGGCCAGCGCGT
GCGGCTCAGTCAGGGAGACATAGCTCAAGCCCGGAAGCTGTACAAATGCCCAGGTAACTATGAGCCGTGGGGAC
TGCC
>hg38_circ_0002296_junction_seq
TTTTGATTCTTGGGACTGACTTTCTACTACAAACATTGTGTTTATTCTCTTGGCTTTTATAGGGGCTTGAGAAA
TAACTGGAATTATCCCACTTTACAGAGGAAGGAATAGAGGCTCCAAAAGATGAAGAAACTCCCTCATATTACCA
CAATACTTTCAAGATCTGGGAGCTTCAGGTTCCTTGCTCCCTTGGCTTTGCCGTTGATAACTTCCTGTCCAATC
CTACCAAAGTCTGGAACTGGAACATCCAAGGGTTGCCCTTAGACGCCTTCTCTACTGAGAATGGCATCATCATC
AAGC
>hg38_circ_0002298_junction_seq
TTTTGATTCTTGGGACTGACTTTCTACTACAAACATTGTGTTTATTCTCTTGGCTTTTATAGGGGCTTGAGAAA
TAACTGGAATTATCCCACTTTACAGAGGAAGGAATAGAGGCTCCAAAAGATGAAGAAACTCCCTCATATTACCA
CAACTCTGGCAGTTACGGTTGTTGAAAATTTCAAAGATGGTGTCATCGTGAAGAACATCTGGCTCTTGATCCCA
GTGCTTGGTCCCACAGATTCCATTCAGGATGCTATGTGACATAAACACCAGGAATGTGCTGTCTGAGGACTGGT
GCTC
>hg38_circ_0002704_junction_seq
GCAGCTGCCGTCCATCGAGATCACGCCCTCCAGCGACGAGGACACCCCGTGGTCCAACTGCTCCACACCCAGCG
CGTCCCCGCGCCGAAAACGCTTCCTCCTCCGCAAGTGGCTGAGGGTGAGGGAGCGGAAGGAGTGCAGTGAAAGC
AGCGCCAAGCCGGCTCCCCAACCTGGTCTGCTGAACGCAAACCGCTGAGAGTTTGCTGCACCGCCCCCTGGGGC
```

CCCTGGCTGCCGAGTCCCGCTAAGGCAAAGACGCCAGCAAGCGAGGAAGCGCAGCGGAAGAAAAACAAGCGGGC
GCGC
>hg38_circ_0003071_junction_seq
CCTTGTTAAACAGGACTACTACCGTTTCCCCTCTGTGTCCAAATTGAAACCAGAGGACTTTGACAATGTTCAGT
CCCTCCTGACAAACAGTATTTACTTACAAGATTCGGAGGTAACAGTGAAGGGATTCAGGATATACGGTGCACCT
TGACCTGGTTTCCCATTGCAAGCATGGAAGAAACCCTAATGCATGAGTTTCAGAGGAGAAGGTAGCAGTCTGAA
CCTGCTGTGTGGCTCCATCAGCAAGTGTGAGTATTAACGTTAGAGCTGTCAGGACTTGTGAAAATTAATGGAGC
CTGA
>hg38_circ_0003340_junction_seq
GGATGCGGCTGAACTTCGACCTGATTCAGGAGCTGAGTCATGAAGCGCGGCGCATGATCGAGGGCGTGGTGTAC
GAGATGCGCGTCTACGCGGTCAACGCCATCGGCATGTCCAGGCCCAGCCCTGCCTCCCAGCCCTTCATGCCTAT
CGACGTGCCAGACGCACCTGCGGCCCCCAAGATCAGCAACGTGGGAGAGGACTCCTGCACAGTACAGTGGGAGC
CGCCTGCCTACGATGGCGGGCAGCCCATCCTGGGTGAGTGCAAGGGCACCGGATGGAGGTGTGAGGGCGCCAAA
CAGA
>hg38_circ_0004879_junction_seq
TAAGTATGTGCTTAATATGTATATTTCTGTTACAGTCAATTTTGCCCATCTTTAGATGTCTGAATTCCCTTGAA
AGAAATATAGAGAAGTGCAGAATATTCACCAGATCTGATAAATGCAAAGTAGTTATTCAATTCTTCTACAGACA
TGTATTTGGGAAAGCAGTTCAAGCTCTATCACGAATTAGTGACGAGTTCTGGCTAGACCCATCTAAAAAAGGTG
TAAGTAAGAAAGTTAACAGATCACGTATCAAGGCAAGAGGAGATGTTTAAAGATCCCAGTCCAGATTGAATGTT
AACT
>hg38_circ_0004898_junction_seq
TTTCTCCCCAGCTCGGAGTCCCTGAGTGTGTGTTTCCTACCCTAGGGCGAGTGAACGTGAAAAATGAAGAAATT
GATGAAATGATCAAGGAGGCTCCGGGTCCAATTAACTTTACTGTGTTCCTCACAATGTTTGGGGAGAAACTTAA
GGGCCTTCACTATCATGGACCAGAACAGGGATGGCTTCATTGACAAGAACGATCTGAGAGACACCTTTGCTGCC
CTTGGTATGTCACCCTCTCTAGCCCTGCATGAGGGTCTCTTTATCTCACATCCATGAGGAGCAGGAGGTGGGAC
CATC
>hg38_circ_0005451_junction_seq
TTCCCAAGGATCGCGGAGGCCATGCATCACCAGGACTGCCTGCTGTTCGCCACCAGCCACCTGATTTCCCTGGA
GGTGTCCCCACCACCCACCCTGTCTCAGAACCTGTCGGGCTCTCCACTCATTACGGTCCACCTCAAGCACAGAT
TGAGTTTTCCGTGGCCAAAATCCTGCCCAAGACCGTGAATTCCTCCCATTACCGCTTCCCGGCCCACGGGCAGA
GCTTCATCCAGATCCCCCACGAGGCCTTCCACAGGCACGGTGAGTGTGGCTGCGCTGGACTCCCTTCCGGGGCG
GCTC
>hg38_circ_0006667_junction_seq
AAAATGTGGTCATTAAAAATGAAAGCATCTCCAGTGAGACCAGCAGATACAGCTTCCACTCTCTCAAGTCCGGC
AGCCTGTACTCCGTGGTGGTAACAACAGTGAGTGGAGGGATCTCTTCCCGACAAGTGGTTGTGGAGGGAAGAAC
AGTGCCTGCCCAAGTGACTGACTTGCATGTGGCCAACCAAGGAATGACCAGTAGTCTGTTTACTAACTGGACCC
AGGCACAAGGAGACGTAGAATTTTACCAAGTCTTACTGATCCATGAAAATGTGGTCATTAAAAATGAAAGCATC
TCCA
>hg38_circ_0006768_junction_seq
TTTTGGGTTGAATATAAAAATATAGAAAAATGTTTAAGCTTTAAGATTATGAATTTTTATTCAAATATTTATAT
TTTACTTTGTAGTTATGATTTTGGATGACAACATGCAAAAACTGAAGGCTCGGTGTTTAGGAAATATTGTGGTA
AAGTTCAAAACATTATTTGTGGCTGGAGAACGATGTGATGTAGAGACCCTGGAATGGTCCAAAAATGTCTTCAG
AGTACCTGTCTTAGACCATTGGTGGCAAACTGGTAAGCATTTTCCTAGCATGTACATAAATAGTAAAGAAATGT
TCCA
>hg38_circ_0006783_junction_seq
AACCATCTAAAATTTACATTGGAAAAACTAAAAATTTATTCAATTTTGTTTTACAGTGATCGGCATGTTGGTAA
AATTTATTCCAAATGTTCTTCTTTTCTGGATTATGTCAGACGGTCTCTAAAGAAGCTTGGATTAGATGAGTCCA
AGAACGTACTCAGGAAAAGTGGGGATTTCCAATGTGCCACTATTTAAAGGAAGAGAGATGGTGCTGTGGTCGTA
ATGCCAGAATGTCAGCATGTTTGGTATGGTTATATTTTTTCCTGGAGTAACAGCTTTATCCAGATGTTGTAGAA
TTTA
>hg38_circ_0006951_junction_seq
CAGGCACTACACAGAGAGGAAGTGGATTTGTTCCAAATCAGATATACATGTCTAGATGTTACAGCAAAGCCCCA
ACTAATCTTTAGAAGCATATTGGAACTGATAACTCCATTTTAAAATGAGCAAAGAATTTATTTCTTATACCAAC
AGGTTGTCTGCATTCAAAAGAGCATTCTATTAAAGCTACCTTAATTTGGCGCTTATTTTTCTTAATCATGTTTC

TGACAATCATAGTGTGTGGAATGGTTGCTGCTTTAAGCGGTAAGTGAACTGTAATCTTTATTCATCTAGAGAGA
ATTA
>hg38_circ_0007593_junction_seq
AACTATTTGTCTTCTTACTACCTGAAGTTATGGATGACCCGACAGTTCACCCCTGTGAACCAGCTAATCAGTCC
TCTGATTATGACAGCACAGAAACAGATGAATCTAAGGAGGAAGCTAAAGATGATGGTGCCAAAGAATCAATAAC
TGTTTAAGATTATTTCAGAAGTTTGACAATTTCCGGATTCTTGTTTGTGGAGGCGATGGAAGTGTAGGTTGGGT
TTTGTCAGAAATCGATAAGCTCAACTTGAATAAACAGGCAAGTGCTAATTCTTTTACTTGCTAGTTAACATGAA
TGCA
>hg38_circ_0007794_junction_seq
TATGTAAAGACCAAGACATTTGGAGGAGGTGGTGGTGGTGCTAGAAGTAATCTCAATATGAATGCTGCTGGTAA
CCGAAATAGGGAAGTTTTACAGAAAGAAAAGTCAACCAAATCAGAGGGAAAACATGAAGGTGTCTATAGAGAAC
TGCTCGAAGGTCCATGTGTTTTGCAAATTCAAAAAATTCGCAATGTTGCTGCACCAAAGGATAATGAAGAATCT
CAGGCTGCACCAAGGATGCTGCGATTACAGATGACTGATGGTCATATAAGTTGCACAGCAGTAGAATTTAGTTA
TATG
>hg38_circ_0008321_junction_seq
GTGATTGCATTTGCTCCCCCTCCAAACCAGCTTCTCCCACCCTCCCACCCCCAGACAGAGGAAGACAAAAAGAA
CCTGCTGCGGCTACAGGACCTGGTGGACAAGCTGCAACTGAAGGTCAAGGCCTACAAGCGCCAGGCCGAGGAGG
CGGAGGAGCAAGCCAACACCAACCTGTCCAAGTTCCGCAAGGTGCAGCACGAGCTGGATGAGGCAGAGGAGCGG
GCGGACATCGCCGAGTCCCAGGTCAACAAGCTGCGGGCCAAGAGCCGTGACATTGGCACGAAGGTGGGTCCCTC
TTTT
>hg38_circ_0008324_junction_seq
TCATTTTTCTCTCTCCATGTCTAGAACACCAGCCTCATCAACCAGAAGAAGAAGATGGAGTCGGATCTGACCCA
GCTCCAGTCGGAAGTGGAGGAGGCAGTGCAGGAGTGCAGAAACGCCGAGGAGAAGGCCAAGAAGGCCATCACGG
ATGCCGCCATGATGGCAGAGGAGCTGAAGAAGGAGCAGGACACCAGCGCCCACCTGGAGCGCATGAAGAAGAAC
ATGGAACAGACCATTAAGGACCTGCAGCACCGGCTGGACGAAGCCGAGCAGATCGCCCTCAAGGGCGGCAAGAA
GCAG
>hg38_circ_0008325_junction_seq
GATGCAGAGACACGCAGCCGCAACGAGGTCCTGAGGGTGAAGAAGAAGATGGAAGGAGACCTCAATGAGATGGA
GATCCAGCTCAGCCACGCCAACCGCATGGCTGCCGAGGCCCAGAAGCAAGTCAAGAGCCTCCAGAGCTTGCTGA
AGGACACCCAGATTCAGCTGGACGATGCAGTCCGTGCCAACGACGACCTGAAGGAGAACATCGCCATCGTGGAG
CGGCGCAACAACCTGCTGCAGGCTGAGCTGGAGGAGTTGCGTGCCGTGGTGGAGCAGACAGAGCGGTCCCGGAA
GCTG
>hg38_circ_0008327_junction_seq
AGCTTTCTGGCCCTCTGGTCCCCAGAGGAAATCTCGGACCTTACTGAGCAGCTAGGAGAAGGAGGAAAGAATGT
GCATGAGCTGGAGAAGGTCCGCAAACAGCTGGAGGTGGAGAAGCTGGAGCTGCAGTCAGCCCTGGAGGAGGCAG
AGGCCTCCCTGGAGCACGAGGAGGGCAAGATCCTCCGGGCCCAGCTGGAGTTCAACCAGATCAAGGCAGAGATC
GAGCGGAAGCTGGCAGAGAAGGACGAGGAGATGGAACAGGCCAAGCGCAACCACCTGCGGGTGGTGGACTCGCT
GCAG
>hg38_circ_0008335_junction_seq
ATGGCCCACAAGCCCCTCATTTCACCTCCAGGAGAGTTGGCCCGGCAGCTAGAGGAAAAGGAGGCGCTAATCTC
GCAGCTGACCCGGGGGAAGCTCTCTTATACCCAGCAAATGGAGGACCTCAAAAGGCAGCTGGAGGAGGAGGGCA
AGGCGAAGAACGCCCTGGCCCACGCACTGCAGTCGGCCCGGCATGACTGCGACCTGCTGCGGGAGCAGTACGAG
GAGGAGACGGAGGCCAAGGCCGAGCTGCAGCGCGTCCTTTCCAAGGCCAACTCGGAGGTGGCCCAGTGGAGGAC
CAAG
>hg38_circ_0008336_junction_seq
TGAGCCCCAGAGGAAGGAAGGCTACCTGTCACTCCCCCACCCCCACCCTTCTCCTGCAGGAAGGAGTTTGACAT
TAATCAGCAGAACAGTAAGATTGAGGATGAGCAGGTGCTGGCCCTTCAACTACAGAAGAAACTGAAGGAAAACC
AGGCACGCATCGAGGAGCTGGAGGAGGAGCTGGAGGCCGAGCGCACCGCCAGGGCTAAGGTGGAGAAGCTGCGC
TCAGACCTGTCTCGGGAGCTGGAGGAGATCAGCGAGCGGCTGGAAGAGGCCGGCGGGGCCACGTCCGTGCAGAT
CGAG
>hg38_circ_0008342_junction_seq
GGGCTGGATGAAATCATCGCTAAGCTGACCAAGGAGAAGAAAGCTCTACAAGAGGCCCATCAGCAGGCCCTGGA
TGACCTTCAGGTTGAGGAAGACAAGGTCAACAGCCTGTCCAAGTCTAAGGTCAAGCTGGAGCAGCAGGTGGATG
ATCTGGAAGGATCCCTGGAGCAAGAGAAGAAGGTGCGCATGGACCTGGAGCGAGCGAAGCGGAAGCTGGAGGGC

GACCTGAAGCTGACCCAGGAGAGCATCATGGACCTGGAGAATGACAAGCAGCAGCTGGATGAGCGGCTGAAAAA
GTAC
>hg38_circ_0008352_junction_seq
GGCTGACCCTTTCCCCTTCCCTCCTGACACAGGGGTGATGGACAACCCCCTGGTCATGCACCAGCTGCGCTGCA
ATGGCGTGCTGGAGGGCATCCGCATCTGCAGGAAGGGCTTCCCCAACCGCATCCTCTACGGGGACTTCCGGCAG
AGGTATCGCATCCTGAACCCAGCGGCCATCCCTGAGGGACAGTTCATTGATAGCAGGAAGGGGGCAGAGAAGCT
GCTCAGCTCCCTGGACATTGATCACAACCAGTACAAGTTTGGCCACACCAAGGTGAGGAAAGGAGACTAATTAA
TTAA
>hg38_circ_0008353_junction_seq
GGTTTAAGGGCCTAAGGGATGGTCCTTGTGGCTCCTAACTCCCCACCTTCATCTGCCTCCAGGAAAATCTCAAC
AAGCTAATGACCAACCTGAGGACCACCCATCCTCACTTTGTGCGTTGCATCATCCCCAATGAGCGGAAGGCTCC
AGGGGTGATGGACAACCCCCTGGTCATGCACCAGCTGCGCTGCAATGGTGTGCTGGAGGGCATCCGCATCTGCA
GGAAAGGCTTCCCCAACCGCATCCTCTACGGGGACTTCCGGCAGAGGTGGGTATGAGGGTGCACCAGAGCTCAT
AGAA
>hg38_circ_0008361_junction_seq
GTGTACTACTCCATCGGGGCTCTGGCCAAGGCAGTGTATGAGAAGATGTTCAACTGGATGGTGACGCGCATCAA
CGCCACCCTGGAGACCAAGCAGCCACGCCAGTACTTCATAGGAGTCCTGGACATCGCTGGCTTCGAGATCTTCG
ACTTCAACAGCTTTGAGCAGCTCTGCATCAACTTCACCAACGAGAAGCTGCAGCAGTTCTTCAACCACCACATG
TTTGTGCTGGAGCAGGAGGAGTACAAGAAGGAGGGCATCGAGTGGACATTCATTGACTTTGGCATGGACCTGCA
GGCC
>hg38_circ_0008372_junction_seq
CTCCAGACGGTGACTGTGAAGGAGGACCAGGTGTTGCAGCAGAACCCACCCAAGTTCGACAAGATTGAGGACAT
GGCCATGCTGACCTTCCTGCACGAGCCCGCGGTGCTTTTCAACCTCAAGGAGCGCTACGCGGCCTGGATGATAT
ATACCTACTCGGGCCTCTTCTGTGTCACCGTCAACCCTTACAAGTGGCTGCCGGTGTACACTCCTGAGGTGGTG
GCTGCCTACCGGGGCAAGAAGAGGAGCGAGGCCCCGCCCCACATCTTCTCCATCTCCGACAACGCCTATCAGTA
CATG
>hg38_circ_0008376_junction_seq
AGCATCACCTCAGAGGGCAGGAGAAGCAGAGCCCTGAGTAGGGGAGGGTGCAACAGCAGGTGCCTCTCCCAGGG
TGGAGGAGAGGAGCGGGGGTAGGGAGGGGGGCTGCAGAGGACAAAGCCACTCGCTGGAGCCTGGGCTCCCTCAG
GAGTGAGAACTGCCCCCATTTTCTCTGCAGAGACTGGGGCATGCTTCTCCTGGGAGCCGGATTGCTGGACCAGG
GGTCTGCTGTCCCAAGCACTCAGCGCCAACCCTTAGCATACTCCAGCCAATGCCACCCCAGGGAAACCCCTTAC
AGAG
>hg38_circ_0008401_junction_seq
GTGACTCTGGACACTTCCCTCCTCAGGTATCGCATCCTGAACCCAGCGGCCATCCCTGAGGGACAGTTCATTGA
TAGCAGGAAGGGGGCAGAGAAGCTGCTCAGCTCCCTGGACATTGATCACAACCAGTACAAGTTTGGCCACACCA
AGGGGTGATGGACAACCCCCTGGTCATGCACCAGCTGCGCTGCAATGGTGTGCTGGAGGGCATCCGCATCTGCA
GGAAAGGCTTCCCCAACCGCATCCTCTACGGGGACTTCCGGCAGAGGTGGGTATGAGGGTGCACCAGAGCTCAT
AGAA
>hg38_circ_0008403_junction_seq
TCCACTATGCCGGCATCGTGGACTACAACATCATTGGCTGGCTGCAGAAGAACAAGGATCCTCTCAATGAGACT
GTCGTGGGCTTGTATCAGAAGTCTTCCCTCAAGCTGCTCAGCACCCTGTTTGCCAACTATGCTGGGGCTGATGC
GCCCCATGGGCATCATGTCCATCCTGGAAGAGGAGTGCATGTTCCCCAAGGCCACCGACATGACCTTCAAGGCC
AAGCTGTTTGACAACCACCTGGGCAAATCCGCCAACTTCCAGAAGCCACGCAATATCAAGGGGAAGCCTGAAGC
CCAC
>hg38_circ_0008412_junction_seq
CCCAGAGTGTTGGAATTGAACCAAGGATGTTGGGACGAGGTTAGAGTGTAACCCTGCCTAGACACAAACAGAAG
ACACTCTTGGCTCCTGGGGTGGACATGGATGGAGCAAGAACAGAGATCCCAACGTAGGGCCAGGTGCAGCACTC
ACTGACTTGCCAGTTGCGAAGGGGGAGGGCTGGTGATTTTGGGAGTTAGAGAAAGATCCCAGGAGAGAAAGAAG
AGAAAGGAAAACCTCTGCATGCACTCAATCTGAGTAATGCCAGTCCCCAGAGTGTTGGAATTGAACCAAGGATG
TTGG
>hg38_circ_0008576_junction_seq
GGGGTGATATGTGTGGCTTCCACACTGGGGCAATAAAGAACCAATGTCTTACTTCCCTTGTTCTTGCTTCCTCT
GCCATGTGCCCCCAAAGGTACAGCTACAAGATGGAGGAAGCTATTTGACCTGCATCAGACTTTACGTGAACAAA
AGGGTGTATCAACCATTTTAAATCTGCCCTCAAAAGCACAGAACAAATCAGACAGCATTTTGCTCACAGAGGAG

AGGGTGTATGAGAACCAAATTCCAGCTTCAGCACATGGCCTTGGGTAAGTCATTTGCTTTCTCTACACCTTAGC
TTTC
>hg38_circ_0008641_junction_seq
GGCAATAGTTATAATAGCTCAGGTCACAGGTGGAATAACTGAATGTCATTAACAATGACATTTGATTATGTTTC
TTCATTTTTTCAGATTATATGTCAAGTTCTGTGTGAAATGAGAGACCATGGGGATTCATTATTGCTGGAGTTGA
CGGGAGAAGGTGTGACGTTTCTTGGTCATAGGAGGGAGTGTGATCTGACACCAGAGGACTTAATATAACATTGC
AGTGTTAACATCTTCACTGGCAGATTCATGGACTTTCCCCCTCCTGAATGGTAACGTATCTTCTTATCTGAATC
AAGG
>hg38_circ_0008778_junction_seq
AATGCCCTCTTACCTCTTTTTCCCTTCCTGAAACAGTTGGCAAAATCAAGTCTTTTAGAGCATAGAATTGCGAC
GATGAAGCAGGAACAGAAATCCTGGGAACATCAGAGTGCGAGCTTAAAGTCACAGCTGGTGGCTTCTCAGGAAA
AGCTGCACAGATGTCCCGATCTCTCTGACTTCCAGCAAAAAATCTCTAGTGTTCTAAGCTACAACGAAAAACTG
CTGAAAGAAAAGGAAGCTCTGAGTGAGGAATTAAATAGCTGTGTCGATAAGGTAGTAGAAGTAATAAGACTTTA
CCAT
>hg38_circ_0008900_junction_seq
GTTATTCTAACTTTTAGTACTGAAGATGAAAAGGAAGAAGAAAGCAGTGAAGAGGAAGATGAAGACAAGCGCCG
TCTCAATGATGAATTACTAGGAAAAGTTGTAAGTGTGGTGTCTGCAACGGAGAGGACTGAATGGTATCCTGCTT
TGTGTTTGATGATGGTGATGAGCGAACATTGAGACGTACCTCACTTTGTCTGAAAGGAGAGAGACATTTTGCAG
AGAGTGAGGTAGGGTGACACGGATGGACGATTTGGATTGAACTACAGGTACTGATCTAGAGGTAAAACGTAATT
TAAT
>hg38_circ_0009264_junction_seq
GGAATGGGCTTGGAGCCAGGGCTCCCAGTTACGGATGTGCAGCCAGCCCCATCAGTGGCTTCATCCTCTGTACT
GGGCTCCTGCATCTCCGGGGCTGCTTCCCACCCAGCCCCCACATTCCCAGGAAGAGTACGCTAGAGTTCCTCAC
CTTAAAAGTGTCTGCCATCACCTCCATTCACCCACCCCAGTAGAACTGGCTCCTGCAGCTTCCCTGCTTCCTGG
CACCCCACTGTGAAACCATTTCTGACCTGCAGTTGCAGACTAGGTGTGACTTGCATTTAAAATAAGATCCTCAG
CAAG
>hg38_circ_0010585_junction_seq
GCCAAATAATGCCAAAATATAGGTCTAAAAAGAGTGAGAACGGAGATGGTTGCTCCCAACTTCTGTCCTCATGG
ATCATCACTGTTTCCCAATCCTCATTGCAGGAAACGGCTCACAATCCAAGAGGCTCTCAGACACCCCTGGATCA
CGCTTAAGTGGAGCATCCCCTTTCCTGGGAGACACGAAGCAGGAAACACTGGCAAATATCACAGCAGTGAGTTA
CGACTTTGATGAGGAATTCTTCAGCCAGACGAGCGAGCTGGCCAAGGACTTTATTCGGAAGCTTCTGGTTAAAG
AGAC
>hg38_circ_0010795_junction_seq
CAGAGGACCCGTGATGTGAAGTGTGTGAGCAACATTGGGGATGTGGTTGACGATGAGGAATGCAACATGAAGCT
CCGGCCGAATGACATTGAGAACTGCGACATGGGACCCTGTGCCAAGAGCTGGTTCCTCACCGAGTGGAGCGAAA
GGGGGAACCCTTCAATGGCCAGATGGTGACAGAAGGCAGGAGCCAGGAGGAGGGAGAACAGAAAGGGAGGAACG
AGGAGAAGGAAGACTTGCGTGGGGAGGCCCCTGAGATGTTCACCTCAGAATCGGCACAGACCTTCCCAGTCAGG
CATC
>hg38_circ_0011145_junction_seq
GGCAGAAGTGATGGTATGTGACCTGGAAACTAGATCATAAATACAGTGTGGCTTCTACTCTTATTTCTCTGTTT
CTCTGGCTTCTTTCTCTCAGATCACTCACTCTGGCAGAAACCATGTTATAATCAGCGCTGTGAAATGGCTCATG
AGGTAAGGACACTAACAGACACACAGGAGAGGCTTAGTAATTTGCTGTGGTGGCCAGACTCCACGATGGTCCTG
GTGAGATCCGCTTCCTGGTATTCATGTCTTTATTTCATCCTCTCCCCTTGAGTGTGGACTGGACTTAGTGACTT
CCTT
>hg38_circ_0011200_junction_seq
CTATTTTTCCTTCATTTATTTATTGAACAAGGGTACTATTGAGAACCAACTAACTTGGAGCCGTGTATGAGCCA
CTGGGAATAGACTAAATGAGATAATCAACGTCTTGATCTCATATTCTTTCATTCTACAGAAGGAAGCAGGCAAC
AGCCAGATTGCCATGCCTAAACCACTTTCTTTGAAGTTTATGGAAGCACCACTGCATTTATGCCAGAAGGTGAA
AGTCCAGAACAGGTGGGTGAATCTCCAGGTGGGTGATATCGTTCTCAGTAGCACAGATACATGAAAGCAAGCAA
GGGG
>hg38_circ_0011716_junction_seq
TCACAGGCAGAAAAAGTACATGAGTTAAATGAAGAAATAGGAAAACTCCTTGCTAAAGCCGAACAGCTAGGGGC
TGAAGGTAATGTGGATGAATCCCAGAAGATTCTTATGGAAGTGGAAAAAGTTCGTGCGAAGAAAAAAGAAGCTG
AGCGCATGGATTTAGGAGAATGTACCAAAATCCACGACTTGGCCCTCCGAGCAGATTATGAGATTGCAAGTAAA

GAAAGAGACCTGTTTTTTGAATTAGATGTAAGTTTTATTTAATGTTGATTTATTCCTACTGCGAAGAACCCAAT
ATTG
>hg38_circ_0011717_junction_seq
CTTGGTTCACCCCAGCCCTGGCATCTGCCTCAGAACCCTTCATCCTCCAGGTATCGCATCGGGCCTGCAGAGGT
TGAAAGCGCTTTGGTGGAGCACCCAGCGGTGGCGGAGTCAGCCGTGGTGGGCAGCCCAGACCCGATTCGAGGGG
AGGGTGACCCAGAGAAGACAGCTAAAGTGGAATGTGGGGACTTCTACAACACTGGGGACAGAGGTAAGATGGAT
GAAGAGGGCTACATTTGTTTCCTGGGGAGGAGTGATGACATCATTAATGCCTCTGGGTAGGTGTGGCTGATGCT
GGGC
>hg38_circ_0012001_junction_seq
TTGGACCGCGAGGCAGAGCGTGTACGGGGTGAGGCAGGGGTCGCCTTGCGCCGGGAGCTGGGGAGCCTGAACTC
TTACCTGGAGCAGCTGCGGCAGATGGAGAAGGTCCTGGAGGAGGTGGCGGACAAGCCGCAGACTGAGTTCCTCA
TGACACAGCTGCCACAGCAGAAACTGCAGCTGCAGGAGGCATGCATGCGCAAGGAGAAGAGTGTGGCTGTGCTG
GAGCATCAGCTGGTGGAGGTGGAGGTGAGGACTTCACAGGGCCATGTCTGAGGGCTGGGGGCCAGGCTAGGGGT
CTCC
>hg38_circ_0012289_junction_seq
CCAACAAAGAAATATATTGTCACATGACTTGTGCCACAGACACGAATAACATCCAGGTGGTGTTCGACGCCGTC
ACCGACATCATCATTGCCAACAACCTCCGGGGCTGCGGCTTGTACTGACCTCTTGTCCTGTATAGCAACCTATT
TGAACCGCATGCACGAGTCTCTCATGCTCTTCGACTCCATCTGTAACAACAAGTTCTTCATCGATACCTCCATC
ATTCTCTTCCTCAACAAGAAAGATCTCTTTGGCGAGAAGATCAAGAAGTCACCTTTGACCATCTGCTTTCCTGA
ATAC
>hg38_circ_0012392_junction_seq
GTCACCATCCATCCTGCCAGCAGACTGTGATCTCCTGAGGGGGACTGTCATAGCCCAGCCCCAGCCCAGAGCCT
GTCACATAGGATGACATCAGGAAATGTCTGCTATGGAATGAAGGCAGGGTGGCTCTATTTCCTCTACATGCCTC
CGCCTGAACTGGAAACCAAATGGCCATCAACAGGTGAAGGGATAAAGAAACTTCCATCCATACAATGGAATACT
ACTCAGCAAGAAAAAGGAACGAACCATTGATAAACAAAGCAATGTGGATGCATTCTGCAGACAGCATGCTGAGG
GAAG
>hg38_circ_0014084_junction_seq
GCCCGAAGGGAGATCGGCACCTTAGCCACTGTCCAGCGGCTGCCCCCCGGCCAGAAGGTCATCGCCCCAGAGAA
CCTACCCCCTCTCACGCCCTACTGCAGGAGACCCCTCAACTTTGGCTGCCTGGACGACATTGGCCATGGGATCA
AGGCCACAGACAAGCGGAAGGCGCTGGAGGAGACCATGGCCTTCACTACCCAGGCACTGGCCAGCGTGGCCTAC
CAGGTGGGCAACCTGGCCGGGCACACTCTGCGCATGTTGGACCTGCAGGGGGCCGCCCTGCGGCAGGTGGAAGC
CCGT
>hg38_circ_0014370_junction_seq
GGCCAGAGCAGCTCGCCTCTCAGGTGCTGAACCAGATGATGAAGAGTATCAGGAATTTGAAGAGATGCTGGAAC
ATGCAGAGTCTGCACAAGTAAGAACAGTGGGGATAGAAAATAGAACACTTTACTTCTTCCTAAAGAGGCTATTA
AGGGTCCACATATAGCCTCAGTTACATTAGCTGCTTATGAATGCAATTCAGTTAATTTTCCCGAACCACCCTAT
CCTGATCAGATTATTTGTCCAGATGAAGAGGGCACTGAAGGAACCATTTCTTTGTGGAGTATCATCTCAAAAGT
TAGG
>hg38_circ_0014555_junction_seq
AGAAGGACAAGGCGATTGTGGCCCACAACAGACATGGCAACAAGGCTGTGTACTCAGTCATGGCCATGGTGGAG
CACAGTGGCAACTACACGTGCAAAGTGGAGTCCAGCCGCATATCCAAGGTCAGCAGCATCGTGGTCAACATAAC
AGAATCCTTCTCTACACCCAAGTTCCACATCAGCCCCACCGGAATGATCATGGAAGGAGCTCAGCTCCACATTA
AGTGCACCATTCAAGTGACTCACCTGGCCCAGGAGTTTCCAGAAATCATAATTCAGAAGGACAAGGCGATTGTG
GCCC
>hg38_circ_0015207_junction_seq
TTTTAGAAACAGATCTCCCACAGAGTGTGGTCTATCAAAATGAAGAGGGTAGGTGGGTCACAGACCTTGCCTAT
TACACATCTTTTAATAGCAAACAAAATTTAAATGTGTCTCTAAGTGATGAGATGAATGAAGACTTCAGATCTGG
TTTGAGATGGAAGATTTTCGAGGTATAGCAGAAGAATCATTTCCAAGCTTTCTCACCAATTCATTATTTGGTAA
CAGTGGGATTTTGGAAAATGTCACTCTTTCTTCAAATCTTGGCTTGCCTGTTGCTGTTTCTACACTTGCTAGGG
ATAG
>hg38_circ_0015231_junction_seq
CTTGTAGACAGGCACGTCACCCAGTGGATGAGCTCTATCACGACACCGTCTCCCTATTTGGAGAAATATTCTGC
AGCGTATTTACTGACCAATATTTGCTGAATACCTGTTGTTTACTAGGACTTCGCTAAGTATTGGGCATCCAGGG
ATGGAAGGAGCAAAAGCCCCTTGCCATCCCTGTGTGACAGCAATGAGGTGAACCCAGGGGCCGAAGCCCACCCA

GGGAGTTGATGGTAAGACTCACCTTCGTTTTCCTTCCCCTCCTCAGAAGTGTGGCTCCTTCCTCTGCATTCGCT
GGGT
>hg38_circ_0015472_junction_seq
GACTCAATGGTTCTTGGATGGAAGCAACCAGATAAGATTGGAGGGGCAGAAATTACTGGCTATTATGTGAACTA
TCGCGAGGTCATTGATGGGGTACCAGGAAAATGGAGAGAAGCCAATGTCAAGGCTGTCAGTGAGGAGGCATACA
AGATTCACTTGTCATGGATTAGTGACTGGTCAGAGTTATATTTTCCGGGTCAGAGCAGTCAATGCAGCTGGACT
TAGTGAATATTCCCAGGATTCAGAAGCTATTGAAGTCAAAGCTGCTATTGGTAAGTTCCTTATGCATAACTGTA
TGGG
>hg38_circ_0015569_junction_seq
GATTAGCTCAGAGAGTTGCATGCCGCTTCTGATGTCAGCTGCTTCTTTCTTAGACAGCGCAAAGATGAGCTGGA
ACAGAGAATGTCTGCTCTCCAGGAGAGCCGGAGAGAGCTAATGGTCCAGTTGGAGGGTCTCATGAAGCTACTAA
AGCATGCTTGAGAGTTCAAACCGGCTTGATGAAGAACACAGGCTAATTGCCAGGTATGCGGCAAGGCTGGCAGC
AGAGTCCTCTTCGTCTGTAAGTAGTTGGAGTAAAAGGATTCGTCTGTTGGCATCTGGGATCCTTGAATTTTATA
TGTC
>hg38_circ_0015718_junction_seq
CAGCAAAGACTCACAGACCTCAGAGTTTGGTCCACTGGACCCAGGCCCACTGCTGGATAAAAGGTGCAGCACCC
ACTGGCCACTTCCACGGTTTACATCTTGCTTCAAGGCCTTGTGCCCGATCAGCTGAGCCCCTGTGCCAACCCTC
AGGATCCACACATCTGTGTATCTCACTGTCGGTGTGGTGTTTGATTTTCTGGTGGCCATGGAACTCTCCGTTGT
AAAGAAAAACATCTTCTCTCAGCAAGTAGTAAGTATTCTGTGTACAATACTTTGACACCATAGGAATACCCAAT
TCCT
>hg38_circ_0015928_junction_seq
CGGGCCGTGATCACCACCATGAAGGAGCGCCGGGTGGGCAGGATCGTGTTTGTGTCCTCCCAGGCAGGACAGTT
GGGATTATTCGGTTTCACAGCCTACTCTGCATCCAAGTTTGCCATAAGGGGATTGGCAGAAGCTTTGCAGATGG
AGGCACAGGAGAAACTGGGTCCAGTGGACATGCTGGTAAATTGTGCAGGAATGGCAGTGTCAGGAAAATTTGAA
GATCTTGAAGTTAGTACCTTTGAAGTAAGTAAAAATGTTTGCTCACTGCAGTAGCACATATGCTAAAAGCAGAG
CAAG
>hg38_circ_0016025_junction_seq
TATATATAGGAGATACTTGGTAAAAGTAAGACAAAATGAATAAATAATGTAAATCTTTCTTAATTTTCCAGGTG
ATTTTTGAAGTGATAACTTCTGGACATCAAGGCTATCTCGCTATCGATGAGGTGAAGGTGTTAGGACATCCATG
TAGTTCTTTCATGCTGGTGAATGCCTCTGGGAGACCTGAGGGGCAGAGAGCCCACCTGCTCTTACCCCAACTTA
AAGAAAATGACACCCACTGCATCGATTTTCACTATTTTGTGTCCAGCAAGAGTAATTCTCCTCCGGGGTTACTC
AATG
>hg38_circ_0016043_junction_seq
ATGACCGGCAGCCAGTCGATTCCACGAGCTATAAAATTGGACACCTTGACCCAGATACAGAATATGAGATTAGT
GTGCTCCTGACCAGGCCAGGGGAGGGTGGCACTGGCTCTCCTGGTCCAGCTCTCAGGACAAGAACAAAGTGTGC
TGCCAGGACTCCTCACTTCCTGCGGATTCAGAATGTGGAAGTTAATGCTGGCCAGTTTGCTACCTTCCAGTGCA
GTGCCATCGGCAGGACCGTGGCAGGAGACAGGCTCTGGTTACAGGTACAGTAACTCATTTTCATCAGTTGATGA
GAGT
>hg38_circ_0016067_junction_seq
TCAGAACAGTGAGATCCTTCCTTATCAGTGCTATTTAAATAGAATTACAGATATTTCTATTTGTCTTAAAGAAA
ACATGAATAATGATTTTTCCCTCTCTTTATTTGACACAGGAAACCAAAATAGACTGTGTCCAAGTGGCCACAAA
AGTGTCTATCAAATAGTTGTTGAGGAAGAACGTCCTCGAAGAACTAAAAAGACGACAGAAATCTTAAAGTGCTA
CCCAGTGCCAATTCACTTCCAGAATGCTTCTCTGCTGAACTCACAGTACTACTTTGCTGCAGAATTTCCTGCAG
ACAG
>hg38_circ_0016325_junction_seq
CCACGCCCCTCCCACCCCCAGAAGTCTGTCCAATCATGTCCCACTCATTTGTTCCCCGTTTCCTCATCCTTGCC
AGCTCCAAAAGATGTGTGGGTATCAGGGAACCTCTGTGGGACGCCTGGAGGAGAGGAACCTTTGCTTCTATGGA
AGCTGGAACCGGAGCTGAAGACCATACCCCTGACCCCTGTTGAGATCCAAGATTTGGAGCTAGCCACTGGCTAC
AAAGTGTATGGCCGCTGCCGGATGGAGAAAGAAGAGGATTTGTGGGGCGAGTGGAGCCCCATTTTGTCCTTCCA
GACA
>hg38_circ_0016424_junction_seq
AGCCCCCTCTCTTCTCCTTCGCAACAGGACACTGGACCTGCAGGAGCAGCTGCAGTCTCTGCGCAGGGAGCTGG
AACAGGTGGCTCAGAAGGGGCGAGCCAGACGGTCTCAGAGTGCCGAGCTGAACAGGGATTTATGCAAAGCCCAC
AGGGCCTTCCTGCTGTCCCTGGTGGCACCAGTGGCATTGATGGTGGCCAGGTGGGCAGAGCCTGGGACCCAGAG

AAGTTAGCCCAGGAACTGGCAGCATCGCTCACCAGGTGTGTGCCTGGGTCCTGGGCTGTGCGGGGGGTGGGCAG
GCAG
>hg38_circ_0016462_junction_seq
CCTGCCTCGGCCCACGGTGCCCCATCATCACGGTCTTCACCTTCTCTCCGCCCCAGTTCAAGCGGATCCTGAAC
CGGGAGTTGACCCACCTGTCCGAAACCAGCCGCTCCGGGAACCAGGTGTCCGAGTACATCTCCCGGACCTTCCT
GGCTTTGACCTGGAAAATGGGCTCTCGTGTGGGAGGAGGGCCCTGGACCCTCAGTCCAGCCCTGGCCTGGGCCG
GATTATGCAGGCTCCAGTCCCGCACAGCCAGCGGCGCGAGTCCTTCCTGTACCGCTCAGATAGCGACTATGAAC
TCTC
>hg38_circ_0016603_junction_seq
AAGTTTGCACGTTTTCCTTCTCTGGCCCAGGTGGTGAAGTGTCTGTTAGATTCGAATGCAAAACCCAATAAGAA
GGACCTCAGTGGAAACACGCCCCTCATTTACGCCTGCTCCGGTGGCCATCACGAGCTTGTGGCACTGCTGCTAC
AGAGGCTGGCGAAGGTTCCTGCCAGTGGGCTTGGTGTGAACGTGACCAGCCAGGACGGCTCCTCCCCGCTGCAT
GTCGCCGCCCTGCACGGCCGGGCGGACCTCATCCCCCTCCTGCTGAAGCACGGGGCCAACGCAGGTGCCAGGAA
CGCA
>hg38_circ_0016928_junction_seq
TTCTCACAGTGCCTCACACCTCTCCTTTGCTCCCTGCAGAAAGACGAGCTTTACCTAAATCTGGTGCTGGAATA
TGTGCCCGAGACAGTGTACCGGGTGGCCCGCCACTTCACCAAGGCCAAGTTGACCATCCCTATCCTCTATGTCA
AGGTGACAGCGGGAAGGTGACCACAGTCGTAGCCACTCTAGGCCAAGGCCCAGAGCGCTCCCAAGAAGTGGCTT
ACACGGACATCAAAGTGATTGGCAATGGCTCATTTGGGGTCGTGTACCAGGCACGGCTGGCAGAGACCAGGGAA
CTAG
>hg38_circ_0016983_junction_seq
CCACTGGGCTCACAGGGGCTGTGAAGTTGGCCAAAGGGACTGTCCAGACCGGCATGGACACCACCAAGACTGTG
TTAACTGGTACCAAGGATGCTGTGTGCAGTGGGGTGACCGGTGCTGCGAATGTGGCCAAGGGGGCCGTCCAGAT
GGGTGTAGACACGGCCAAGACCGTGCTGACCGGCACCAAGGACACAGTCACTACTGGGCTCATGGGGGCAGTGA
ATGTCGCCAAAGGGACTGTCCAGACCAGTGTGGACACCACCAAGACTGTCCTAACTGGTACCAAGGACACCGTC
TGCA
>hg38_circ_0017433_junction_seq
CTCCTTACAGCCAAGGCTGATGACATTGTTGGCCCTGTGACGCATGAAATCTTTGAGAACAACGTCGTCCACTT
GATGTGGCAGGAGCCGAAGGAGCCCAATGGTCTGATCGTGCTGTATGAAGTGAGTTATCGGCGATATGGTGATG
AGGCCATCTCGGAAACGCAGGTCCCTTGGCGATGTTGGGAATGTGACGGTGGCCGTGCCCACGGTGGCAGCTTT
CCCCAACACTTCCTCGACCAGCGTGCCCACGAGTCCGGAGGAGCACAGGCCTTTTGAGAAGGTGGTGAACAAGG
AGTC
>hg38_circ_0018575_junction_seq
TGTGCCTCTTCAGGGACCGCTTGGAGCAGTTAGAAAGGAAGCGGGAGCGGGAGCGCAAGATGCGGGAGCAGCAG
AAGGAGCAGCGGGAGCAGAAGGAGCGCGAGCGGCGGGCAGAGGAGCGGCGCAAGGAGCGGGAGGCCCGCAGGGA
AGTGTCTGCACATCACCGAACGATGAGAGAGGACTACAGCGACAAAGTGAAAGCCAGCCACTGGAGTCGCAGCC
CGCCTCGGCCGCCGCGGGAGCGGTTCGAGTTGGGAGACGGCCGGAAGCCAGGTGAGGCCAGGCCGGCGCCTGCG
CAGA
>hg38_circ_0019311_junction_seq
ACCATACGAAAAAAACTGTCCCAGTTAAGGCCCAGGCTCAGCCTATACAGCTGACAGACCTCTATCCCGGGATG
ACATACACGTTGCGGGTTTATTCCCGGGACAACGAGGGCATCAGCAGTACCGTCATCACCTTTATGACCAGTAC
AGCCCCTGATGAGCAGTCCATATGGAACGTCACGGTGCTCCCCAACAGTAAATGGGCCAACATCACCTGGAAGC
ACAATTTCGGGCCCGGAACTGACTTTGTGGTTGAGTACATCGACAGTAAGCATTGCTGTGCGGGGTGGTGGTGG
CGGC
>hg38_circ_0020161_junction_seq
GAACTTTTTCATGTTTCACATATCCGTATATCTGCAGGGGAGGCTTGTTGGTGGACCATACACCCTGCCTCTAA
GCAGCGATCAGAAGGAGAAAAAGTACGAGTTGGAGATGACCTCATCTTAGTTAGCGTGTCCTCTGAAAGGTACT
TGTATCTGTGCTGCCTGTCCACCTCCCGGTCTTCAACTGATAAGCTGGCTTTTGATGTTGGCTTGCAAGAGGAC
ACCACAGGTAAGCATCTTGTGCTGCGGGAAGCCAGGTTCAGAGAGAACCCTGCAGGGGTTGGATTGGAAGAAGC
CGGG
>hg38_circ_0020181_junction_seq
GGAAGCCACATAAGATGGGGACAGCCATTCCGACTACGCCATGTCACAACAGGAAAATACTTGAGTCTCATGGA
AGACAAAAACCTTCTACTCATGGACAAAGAGAAAGCTGATGTAAAATCAACAGCATTTACCTTCCGGTCTTCCA
AGAACTGTTCATTATGAAGGTGGCGCTGTGTCTGTTCATGCACGTTCCCTTTGGAGACTAGAGACGCTAAGAGT

TGCGTAAGTAGAACTTCTAAACACAGCCTAATGCACCAAGTGTACCAAATAGCTCAGCGTTGTGCTTCTGTGTC
AGCC
>hg38_circ_0020269_junction_seq
TTTAAAGTATAAGTTAAGTCTCTCTTCACAAGGTTTTTAATGAGGCACTGTTTTTTCACACAAATGATCTAGGT
TTCTGTGGACGCTGCCCATGGTTACAGTCCCCGGGCCATTGACATGAGCAATGTTACACTATCTAGAGACCTGC
ATTTGGCAAATGGATGGATTTATGGAGAAATATATTCAGACTCTTCTAAGGTTCAGCCATTAATGAAGCCATAT
AAGCTATTGTCTGAAAAGGTAAGGATTTTTTGTTTGTTTTAGTTTGTAAAGTTTTTATTCTTTTGAAAAATGTG
TTTT
>hg38_circ_0021276_junction_seq
TTCATAGTGGTTAAACTATCAAAGCCTCATAATGTAGTAAATAATGCAGTGCTAAATAAATATTGTAATCCTTT
CTTGTGTGTCTTTTGTCTTCCAGCATTCAGCTATTTTGAAATGGCCAACTCCAGAGTGTGAGATGGTAGCATAC
AGAACAATATAGCTGAAGTACAAGAATTACATTCTGAATTAATGTGGAAAGATTTTATAGACCACATCAGTAGT
CTCCTACACAGTGTGGAAGTGGAAGTCAGTTACTTTGCAGCTGGAATTATTGCCCATTTAATATCCAGAGGTGA
ACAA
>hg38_circ_0021284_junction_seq
GAAATTTTGTTCCCCCCTCTTAGTTTTCAGATAGAACCATTCCCACTGATAAGCATGTTGACCTCCTGATCAAT
AAGTATGGATTGTCTGCTCACCCAGTTGCTCCTCAGATGTTTGGGTATGCTGGAAAAGAACATATGGAAAAATA
TGGTGTGGCAGAATGTGTCTTGGCTCTTGGGTTTGAGAAGATGAGTAAGGGAAGCCTTGGAATAAAAGTGAGTG
TTATTTTGGCATAGTTACTAAATGAGCTAATATAACCCAAAAGTTGAAAGGTGATACTTACATGTATGCATAGA
TCTT
>hg38_circ_0021425_junction_seq
GACCCTCATTTCAAGATCAACAATGCAGTTTCCAATATCATTTGCTCCATCACCTTCGGAGAACGCTTTGAGTA
CCAGGATAGTTGGTTTCAGCAGCTGCTGAAGTTACTAGATGAAGTCACATACTTGGAGGCTTCAAAGACATGCC
AGGATTGATTATGTCAAGTGGCCAGGCATGGAAGGAGCAAAGAAGGTTCACTCTGACAGCACTAAGGAACTTTG
GTTTAGGAAAGAAGAGCTTAGAGGAACGCATTCAGGAGGAGGCCCAACACCTCACTGAAGCAATAAAAGAGGAG
AACG
>hg38_circ_0021510_junction_seq
CCCACACACACACTTTCACCGCCCTTCGTTTCCCAGAGCTGAATGGAGGCCATTCCTACTGCCGCAACCCAGGG
AATCAAAAGGAAGCTCCCTGGTGCTTCACCTTGGATGAAAACTTTAAGTCTGATCTGTGTGACATCCCAGCGTG
CGAGATGAGTATGAAGAAGATGGATTCTGTCAGCCATACAGAGGGATTGCATGTGCAAGATTTATTGGCAACCG
CACCGTCTATATGGAGTCTTTGCACATGCAAGGGGAAATAGAAAATCAGATCACAGGTAGGTAGCACCAATGAA
ATTA
>hg38_circ_0021663_junction_seq
TAACAGCAGTGGGTCTCTCTCACCTTCTTCTTCTTCTGATTGCCTGGTGAACCGGGGAGGACCTGGCCGGAGTC
ATGTGGCAGCATTAAGAAGTCGTTTCGAATTGGAATATGCTCTAAATGCAAGGTCCTATGCTGCTTTGTCCCAA
AGTCACAATATTCTTCTCTATGAGGATGGGCATGCTGTGGTGGCAGATTTTGGAGGTGAGATACCCCAAAATGG
CATCCTTTTTTTCTTTGTTCCTAGCTGGTACAATATGGTGCCTGATATTGTACCATGAAACTGAACACTGAACA
CTGA
>hg38_circ_0021668_junction_seq
TAACAGCAGTGGGTCTCTCTCACCTTCTTCTTCTTCTGATTGCCTGGTGAACCGGGGAGGACCTGGCCGGAGTC
ATGTGGCAGCATTAAGAAGTCGTTTCGAATTGGAATATGCTCTAAATGCAAGGTCCTATGCTGCTTTGTCCCAA
AGGTCATGAGGCATTTTGGTAATTTCTACTGTTCAATGAGCATCTTGGTGTTCTCTTAAGATGCCAGGTAAATA
ACTACATTCCTTAATTTGCATAGAATTTAAACTGAAATGTTAAAGCATTTGACTATAAATTCAGAGAGCTCACA
TCCA
>hg38_circ_0021761_junction_seq
GGCACCAAAATTACTTTTCCAAGAGAAGGAGATGAAACACCAAATAGTATTCCAGCAGACATTGTTTTTATCAT
TAAAGACAAAGATCATCCAAAATTTAAAAGGGATGGATCAAATATAATTTATACTGCTAAAATTAGTTTACGAG
AGGGTTGAAAGGAGGAGCAGGAGGTACTGATGGACAAGGAGGTACCTTCCGGTACACCTTTCATGGCGATCCTC
ATGCTACATTTGCTGCATTTTTCGGAGGGTCCAACCCCTTTGAAATTTTCTTTGGAAGACGAATGGGTGGTGGT
AGAG
>hg38_circ_0021918_junction_seq
GAGGGGTGGTGCACTTAAGAAAGGACCAAATGCCAAAGCATTGCAAGTCATGAAGCAGACATTACCCTATAGTG
TGGCAGACCTTGAATGGGATGCAGGTCATAAAACCAATGTCCAGCAGTGTTACTGCTATTGTGGAGGCCCTGGA
GAGAGCCACTGGAAGTGGGGAAATGGTCTGTACAATATGTCAAGAAGAGTATTCAGAAGCTCCCAATGAAATGG

TTATATGTGACAAGTGTGGCCAAGGTAACCATTGATTTCTTTTAATCTTGTTACATACTGAATGACTATGTTGA
AGAT
>hg38_circ_0022137_junction_seq
TGTCTTCGTATTTTTTTTCCAGATGAGTTATGCTTTAAACCATACAAACAGAAAACTAACATTGGAACCTAAAA
TTACTGTCAATGCCAAGATCATTGTGGTTGGTGCATCCAGTGTTGGAATTTCCTTCCTAGAGACATTGGTATTT
TGGACATTGAGTACATACGGTCCCATTACAACATTGAAGATTTCATCTACTTCAGTCACCACCAGCGCGAAGAA
CACGGGCACATGCATCACTTTGCCCTCAACCCCATTTTCCGGCACTACACCAAGTTCTTTCTGAAGGAGATCCT
GCGT
>hg38_circ_0022551_junction_seq
TTGTTTGTGTTTTCATCTTACAGACTGAGGAACTAATCAAAGCTTTGCAGGATCTGGAAAATGCCGCATCAGGG
GATGCTACTGTCCGACAGAAAATTGCTTCTCTGCCCCAGGAAGTGCAAGATGTTTCTCTATTGGAAAAAAATAAC
AGCCAAATCAAATAGAAAGCTTACTTTTCTGTATTTAGCGAATGATGTCATCCAAAACAGTAAAAGGAAAGGAC
CTGAATTCACTAGAGAATTTGAATCTGTCCTTGTGGATGCTTTTTCTCATGTTGCCAGGTATGTTGTCTGTTTT
TTGG
>hg38_circ_0023237_junction_seq
ACACTGCTGTTTCCTCCCACCTAGGAAACCCGCAGACCTGCAGAACCTGGCTCCCGGAACAAACCCTCCTTTCA
TGACTTTTGATGGTGAAGTCAAGACGGATGTGAATAAGATCGAGGAGTTCTTAGAGGAGAAATTAGCTCCCCCG
AGGCTGGTTATGATGGTGAGAGTATCGGAAATTGCCCGTTTTCTCAGCGTCTCTTTATGATTCTCTGGCTGAAA
GGCGTTATATTTAATGTGACCACAGTGGACCTGAAAAGGTAAGACAAGGCGTCTGCCTTACTGAAATAACTGTA
CCTA
>hg38_circ_0023577_junction_seq
AATTTTTTTATCTGATAGGTATTCTTGGATCCCCATCACAAGAAGACCTGAATTGTATAATAAATTTAAAAGCT
AGGAACTATTTGCTTTCTCTTCCACACAAAAATAAGGTGCCATGGAACAGGCTGTTCCCAAATGCTGACTCCAA
AGATCTGTGACTTTGGCCTGGCCCGTGTTGCAGATCCAGACCATGATCACACAGGGTTCCTGACAGAATATGTG
GCCACACGTTGGTACAGGGCTCCAGAAATTATGTTGAATTCCAAGGTAAGGATGAAGTTTGCATAAAAAGAAAT
CCAG
>hg38_circ_0024492_junction_seq
AGCTCTTGGAGGAAGTTGGCCAGAATGGCAGCCGAGCTCGGATCTCTGTTCAAGTCCACAATGCTACGTGCACA
GTGAGGATTGCAGCCGTCACCAGAGGGGGAGTTGGGCCCTTCAGTGATCCAGTGAAAATATTTATCCCTGCACA
CGCCCCATCAGTAGCACCTTTAAATGTCACTGTGTTTCTGAATGAATCTAGTGATAATGTGGACATCAGATGGA
TGAAGCCTCCGACTAAGCAGCAGGATGGAGAACTGGTGGGCTACCGGATATCCCACGTGTGGCAGAGTGCAGGG
ATTT
>hg38_circ_0024608_junction_seq
TTTCAGGTCAAAGTTGCAATCCTGAAATACATTGAGTCTCTGGCCAGACAGATGGATCCAACAGATTTTGTAAA
CTCTAGTGAGACAAGGCTTGCTGTTTCTAGAATCATAACCTGGACAACAGAACCAAAGAGTTCAGACGTGAGAA
AGATCGGTTTGGGCTTGGCCAGCCAGGAAGAATACCTGGTTCTGTGAATGCCATGAGAGTTCTGAGCACAAGTA
CAGATCTTGAAGCTGCTGTTGCTGATGCTTTGGTAAGAGGCACAGTATGTATGTACACAGTCTTTTTGAAATAC
TCAT
>hg38_circ_0024720_junction_seq
ACGTCACAGGGTGGCCTTGTTTGATGTTGCTGTTTTATGAAATTACACTATCCATGTACAACATAAGGAAAATG
TGACCCAGCTGAGAGCGCCAGGCCAGATTCTGGCAACACCGATGTCTGGATGTCAGTGCCAAACCAGCTCCCTG
ATGGTTGTAGGATTTTTCTTCTCAGTCACTTTGCAAGCCAGGGACCCTCGATCGATGACGCCCCGCCCGGGCCT
TGCTCTGCCACGCTGCATGCCCCAGCTCGCCTGTGTGATAGCTTGTACCCACGTTCAGTGGTTCCTGAGCTTTT
GCAC
>hg38_circ_0024722_junction_seq
GTTCTGAAGTATCTTATCCTAATAGTATGCCAAAATAATGTCTAACTTTAGCAAAATCATAGTTCACAGGTAAA
TTGATCAGGAATAGTTCTGAAAAGTGTACTGCAGTTGAAGAAAGCACTATCTAGAGAGACGAAGACAATCATTC
AGGGTGGCCTTGTTTGATGTTGCTGTTTTATGAAATTACACTATCCATGTACAACATAAGGAAAATGTGACCCA
GCTGAGAGCGCCAGGCCAGATTCTGGCAACACCGATGTCTGGATGTCAGTGCCAAACCAGCTCCCTGATGTGAG
GCTC
>hg38_circ_0024783_junction_seq
TATTCATAAACAGTGTTATTTTTTCCCACATAGGAATTGAATACCAGCTTTGAAGAGTGGGAAAAAATAATGAG
AACTCCAAATTCAGGTGCCATGGAGACAGTCATGGGGTTGATAACTCGCATGTTTAAACAAACTGCTATTGCCA
AGATATTTTGGTAAAAGACCTCAGTGGGATTGTAGAGGGGCATCAAATCTTAATGAACTTCACCAGCTATTAAG

TGACATAATGATTAGAAGATTAAAGACTGAAGTTTTAACCCAGCTACCCCCTAAAGTCAGACAGCGTATTCCAT
TTGA
>hg38_circ_0025066_junction_seq
CTTAAATCATCCTAGATGATGGAAGAATCAGAAATGTGCGCAGTGCCTGGTGGTTTGGCCAAGGTGAAGAAACA
ATTTGAGGACGAAATTACTTCTTCCCGTAATACCTTTGCTCAATACCAATATCAACATCAGAACAGATCTGAGC
AGCGACTGCTGGCCCTAATAAGCCTGAGAGTGGATTTGCAGAAGACAGTGCTGCTCGGGGCGAGGGTGTGTCAG
ACCTCCACGAAGTGGTCTCCCTGAAGGAGCGGATGGCGAGGTACCAGGCAGCTGTTTCCAGGGGTGACTGCCGC
AGCT
>hg38_circ_0025881_junction_seq
AATATTGGGTGCCACATTAATTTAATTGATTTTTAAAAATTCATAGCAGTCCATGTAAAGAAACTACTTCAACC
TTCTTGGTAAATATGGAGATGTACCCCAGATTCTCCTCATCAGGGAAGGCCTTACTATGCAACAGCAGCGGGGG
AGAAACTGTGTTGTTTGGGACAGTGATGGCACCAGCTGGATATGATTCTCAGGTTCCCTTGAAATTAGGGATGG
CCTTATGATAGTTCTTGTCCATCAGGCATAAGCAGAAATGTTGGCCATTTGGGAAAAGCTTCAGTTTTATCAGG
TAGG
>hg38_circ_0026650_junction_seq
AGGATTGCCGAATAAGAACTGAGTTTTTCTGGGTTCCTCCAGGTTCTTCGCAGCAATAGCCTGCTGGAGTCAAC
AGACTACTGGTTGCAGAATCAGAGGATGCCCTGCCAAATTGGTTTTGTAGAAGACAAGTCTGAAAACTGTGCTT
CTCAACTTGGAGTCATCACGGATGTATGACGTTTTGGAACCGCAGCAGGGCAGAGGCTGTGGCAGCTCAGGAAG
CGGCCCGGGGAACTCCATCACAGCCTGTAAGAAGGTAACCTTGCCTTACGCCACTTTCTTCTTGTCAGTTGAAC
TTGA
>hg38_circ_0026651_junction_seq
AGGATTGCCGAATAAGAACTGAGTTTTTCTGGGTTCCTCCAGGTTCTTCGCAGCAATAGCCTGCTGGAGTCAAC
AGACTACTGGTTGCAGAATCAGAGGATGCCCTGCCAAATTGGTTTTGTAGAAGACAAGTCTGAAAACTGTGCTT
CTCCATGTGGGTAGTTGAACCAAACTCACCTACCTGCCTCTCTGCTGCCTTGCCCACCAGGAGACACTCCCAGG
AATCCAGGTTGGTTGTGGGGTGTGGGGTCCAGGGGATTATAAAGCCAAGTCGTGATCAGGGCCTCCTCTTCCAT
TCCA
>hg38_circ_0027148_junction_seq
CAAGAGCCGGTAAACCCAGAAGCTTGGCCGTCCATTCCTGGAAGCAGACATTGTGTCTTCGTCCACCAAGCCCC
TGGCTCTCAACACTCACTGTGGTGGCAAGAACATGGCACCTTGACCCTACACAGTTCTTCCATTTTTCAGAAGC
AGATGAGAAGAGAATGAGCCATGTGTTTCCTGAAACATCCAGTGGCCTCACCGGAGAATCTGCAAGAGCCGGTA
AACCCAGAAGCTTGGCCGTCCATTCCTGGAAGCAGACATTGTGTCTTCGTCCACCAAGCCCCTGGCTCTCAACA
CTCA
>hg38_circ_0028297_junction_seq
TTGTGTGCAGTTTTTATTCAGATAGGAAAGGCTGTTAAAATCTTAACACCTAACTGGAAGAAGGGTTTTAGAGA
AGTGTGGTTTTCAGTAAGCCAGTTCTTTCCACAATCCAAGAAACGAAATAAATTTCCAGCATGGAGCAGTTGGC
AGTTCAGTGTTACCTAAATTTACATGAAGACCCAGAGGCCCACGCTCCTTCATTTTGGGCTCCACCGACCTCCA
AGGTTTCAGGGCCCTCTGCCCCGCCTTCTGCACCCACAGGGGAAGAGAGTGGAGGATGCACACGCCCAGGCCTG
GAAG
>hg38_circ_0028322_junction_seq
GACGTGTTATTTTTGGTTTGAAAATGATAACTTTTAAAATATATGTTCAAGGATAATTGGACAGCACTTATATC
TGCATCGAAAGAAGGGCATGTGCACATCGTAGAGGAACTACTGAAATGTGGGGTTAACTTGGAGCACCGTGATA
TGGTATTCAAATAAAGTTAATTGCAGCTTTCTGTGAAAATGTCAGTTTTGATATCACAGAGCGTCATAAATTAT
GTAGAGGAAGAAAACATTCCTGCTCTGAAAGCTCTTCTTGAAAAATGCAAAGATGTAGATGAGAGAAATGAGGT
AAGA
>hg38_circ_0028496_junction_seq
TTGCATTTTTCACAAACTACTAAATATTATGTCTATAATATTTCAGAGTTCTTGGAAAATACTCCATCCAGTCT
GAATATAGAAGATATAGAAGACCTTTTCTCTCTGGCTCAGTATTATTGCAGCAAAACACCGGCTTCTTTTAGGA
AGAAGTAAAAATGGAAGCTTTAAAGAGGTTGACATGTCCTAAGTCACACAGTTAATATGTGGCTCCATTGTCCC
TTGAATTCACATGTTTTGCCTTTGGATTTCATACACTAAGATTAAGGTACGCATTAAATATCAGGTGCAGATAA
TTTG
>hg38_circ_0028591_junction_seq
TTGTGTCCTTAGTTGGATATGGAATGTGCTCTTTTGGATGGAGAACAGAAATCTGAAACAACTGAACTTATGAA
GGAGAAGGAGATTTTGGATCATCTAAACCGGAAAATAGCTGAACTGGAAAAGAACATTGTTGGTGAAAAGACCA
AGAATAACAAGCTGAAGCTATGAAAGGTCATCTTTTCCATCAGATCACGAAGACCAAGGCTTAAATCATCTTAG

GCAGAGATTTCAATTTACAAGTTGTCTTGGGAGAAAGGAAAATATTCAGGCATACCTCTGAGATATTGTGGGTT
TGGT
>hg38_circ_0028615_junction_seq
AATTTGGGGTTCCATCAGATGATGAAAATTTTATGAAGTATATGATGAGTGAGGAGTTGGTGCTGGGCATTTTG
CTACAGGATTTCCGTGATCAGAATATACCTGGTTGTGAGAGTCTGGGGCTTGATCCAACATCCCTCTTGCTCTA
TGCTCACAGATGTGTACAGTACATCGCCCTCTCTGGGTCGTTATTTTACTTCAGTTGAAATAGTGGACTTCAGG
TAAGAGTGTGGAACATTACATTTTGCTCTCTTTCTCTGGAAAGAGATCCCCCCACCCCACCCTTCGTCATTTAC
CTAG
>hg38_circ_0028988_junction_seq
TTGACGTGGTGGAGATCTACAACCCAGATGGGGACTTTTGGCGAGAGGGCCCTCCCATGCCAAGTCCCCTCCTC
TCACTCCGCACCAATTCCACCAATGCAGGGGCAGTGGATGGGAAACTCTATGTCTGCGGGGGATTCCATGGAGC
AGATGGATCTTCCTGATGAAGAACCTGATCGATTAAGCAACAAACTGTTGCAGTATGACCCCAGCCAAGATCAA
TGGAGTGTGCGGGCACCCATGAAGTACTCTAAGTACCGATTCAGTACAGCTGTAGTCAACAGTGAGATTTATGT
TTTG
>hg38_circ_0029662_junction_seq
CCAACAAATCATTTTATTGTTTAAGCTACTTTATGTTGGGTTATCTGTTTCTTGCAGGCAAAAACATGCTAACT
GACAAGAAAATCATGGGTTTTATACAAGGATAACAAGTGCTGGGTTGCTTAGCTATGAGGAAGGCTAGGCTCAC
TGGAATCTCTGAAGAAGAGCAACTTTTCAGACAGGTAGAGTGTGCCTTGCTCCCAAGGAAAAGAGTTCTTGAAA
AAAACAAATGGGAAACATGGCTACAAGAAAACTGGGAAGACTGCAAGGTGAGACCTTTTTCATTTAAAAAAAAA
AAAA
>hg38_circ_0029669_junction_seq
CTCTCAACTCTATTCTCCCCACTCCTCATTCAACCTTCTTTTAGCTATCGCTGCAGTGGGAACTTGTTTCAATG
GAAGAAAATGCTCAGATGGTGAAGGCCAGTAGTGCCAGTGTTCAGAGGGATTTCATGGACCCCACTGTCAATAA
CAGCCAAGTGTTTGTGAAGAACAGCAAGTGACTGTGGTTGGACTTGCACACCCATGTGTTTGGGCATTCACACA
CATTGTCAAGCTCTGCAAACAAGGCTGTACTGGCTCAAGTTGGTATGTGAGCTATGAAAGAAGGTAAGAGTGCT
TCTG
>hg38_circ_0030203_junction_seq
GTGGGGTTGGGAGGCAGAAATGAGGACAGGAACATTTACCTTGTGTTTCTCTCAGCTTCTCAGATGACCAAGAT
GGTGCTGCAGATGGTGCTGAGGATGGAGAACCCACCAAGCCCCGCTAGGAGCCACCTAGACTGGATGCAGAGCA
TGGCAGCTGCTCCCAACCCCAGAACCTTTGCTGTCTTGGGACGGATCACCGCTGCAAGAGGGGAAGTTGCTACT
GTGATGAATTCTGCCATGTGGCACCAGACTGCCACCCAGACCACAGTGTCCTCTGCAACCCTGGTAACTCACAT
ACAG
>hg38_circ_0030354_junction_seq
GGCGCAGCACGTTGAAAAATGAACAGTCGTCGCCACATCTCATCCAGACCACTTGGACTAGCTCAATATTCCAT
CTGGACCATGATGATGTGAACGACCAGAGTGTCTCAAGTGCCCAGACCTTCCAAACGGAGGAGAAGAAATGTAA
AGAAAATGGCCTTACAGCCTGGGACAAACCGAAGCACTGTCCAGACCGAGAACACGACTGGAAGCTAGTAGGAA
TGTCTGAAGCCTGCCTACATAGGAAGAGCCATTCAGAGAGGCGCAGCACGTTGAAAAATGAACAGTCGTCGCCA
CATC
>hg38_circ_0030357_junction_seq
GCAGCTCGCCAGAGAGGCAAACTCCAGCATTTTATATTTCATCTGCCCCCAGTGACTTCACTGTAGGAAGAGGA
AATGCAGAAAAACAAACGTCTTGAAGACTGAAAGCCTGTTATTCAAAGAGGTTAGCAGTAGACTGTTGGAAGTA
AGATCTGCGATTTCCTTCTGGTTGGCTGTCCTGCGTGGGTGCCAAGTTCCACACATGATTTAATGAATAAGAAG
GTCAGATGTCAGCTTGACTATTTGCAGACTTGTGATCTTTGCTATTAATTGTCCCTTTTTAGCATTATTGGAAT
TATC
>hg38_circ_0030598_junction_seq
GGCCTGTCATTACGGTGGATGTCTCCATCTTCCTCCCGGGCTCCATCAACATCACAGCGCCTCAGTGTCACGAC
GGACAGCAGCCTGTGAACTGCCTGAACGTCACCACCTGCTTCAGCTTCCATGGCAAACACGTTCCAGGAGAGAT
TGAAACTGTCTGGGCAGAAGATAAATCCAGTGCTCCGGATGTTTGGTCAGTCCATATCGGGAGGCATTGATATG
GATGGAAATGGCTATCCTGGTAAGCTGTTTTTCTTTAAAGGCACATGAGATAAATGAAGATAAATGCATGAAGT
AGAA
>hg38_circ_0030600_junction_seq
GTTCTGATGGCTGACGTGGCCAAAAAGGAGAAGGGCCAGATGCCCAGGGTCTACTTTGTGCTGCTGGGAGAGAC
CATGGGTCAGGTCACAGAGAAGCTGCAGCTGACTTACATGGAGGAGACGTGTCGTCACTATGTGGCCCATGTGA
AGAGCAAGGCCTGTCATTACGGTGGATGTCTCCATCTTCCTCCCGGGCTCCATCAACATCACAGCGCCTCAGTG

TCACGACGGACAGCAGCCTGTGAACTGCCTGAACGTCACCACCTGCTTCAGCTTCCATGGCAAACACGTTCCAG
GAGA
>hg38_circ_0031448_junction_seq
CTTTCCATGATTGAAATCAGTGATTCATTGGTTTCTTTGTGTGGGCTTTTGAAAGATCATTAGAAACCAACCTG
AAGATCATGAGATTCTTGTGGACACAGGAGCAATGAGTGGCAATACAGTGCTTTCTAATGCAACCTACTTTAAA
AGGATTTCATCAAATGGTCTGCTGCACTGTGCTGGAATTTTGAACTCCCCAGAGTAGTAAACAACATCTTCTCA
AGGGTATACAAGTCATCAGATAGAGAAAACACCCATTATAGAGCTGTGGAGCCCTTACGATATAGTGCTATGCT
GCAA
>hg38_circ_0032125_junction_seq
CCTGAAAACACAGCCAAAGCAAAGGAAGTTCTCAGCAACATCAATCAACTACAACCTCTTATAGCAACCCATGC
AGACCTACTGCTTAATTCTGCAAGCCAGCATTCTCCAGACAGCTTGAAGAATTCTTTAAAGATGCTTTCAGAAA
AAATGCTCTCTACGATGTCATCACTGTGGGAGCCCCAGCTGCCCATTTTCAGGGATTTAAGAATGGTGGTCTTC
GGAAGCTACTCCATAGATTTGAAACAGAAAGAAGAAAGTAAGTAAAGACAGGAGGAAAGCACAGATTCCATTAA
TAAC
>hg38_circ_0032354_junction_seq
TTATTCTTCCTTAGATTCTTCTTTGGAGCAGAATAAAAGATCTGGCCCATCAGTTCACACAGGTCCAGCGGGAC
ATGTTCACCCTGGAGGACACGCTGCTAGGCTACCTTGCTGATGACCTCACATGGTGTGGTGAATTCAACACTTC
CAACATGTAGACTGCCAAAGTGTATGGGATGCTTTCAAGGGTGCATTTATTTCAAAACATCCTTGCAACATTAC
TGAAGAAGACTATCAGCCACTAATGAAGTTGGGAACTCAGACCGTACCTTGCAACAAGGTAATTGGGGGCATGC
CATT
>hg38_circ_0032802_junction_seq
TGTATAGGGGCTGCTGATTTTCCCTTTACATATTTCCCCATCTCCCTTCCCCAGGTGTTGCTGCCATCACTACC
TTTCCCTTCCTCCCCTTTCCTCATGGCAAGACGCATGGAAGAAGAGTGCTGTTAAGGCCTCTCCATTAATCAAC
AGCCATTTCGTAGAGTGACGTTTTCTGTTGTGAGTCAGCCTCAGGACCCACATCAGGGGTCACTGCAGAGTTGC
TATGACAGCGGGCTGGAGGAGTCAGAAACACCAAGCAGTAAGAGTTCATCAGGGCCAAGACTGGGTGCGCTTCC
ACTC
>hg38_circ_0033081_junction_seq
CTAGGGCATTGTGTGAACACTCTAAAGAACGCTGTGAGTCTGTTCTTGGGATTGTGGGCCTACAGTGGCCTGAA
GACACAGATTGCAGTCAATTTCCAGAGGAAAATTCAGACAATCAAACCTGCCTGATGCCTGATGAATATGTGGA
AGACTGCAGCGAGAATCTGTTTCACTGTCACACAGGCAAGTGCCTTAATTACAGCCTTGTGTGTGATGGATATG
ATGACTGTGGGGATTTGAGTGATGAGCAAAACTGTGGTAAGTAGCATTGTTTCCCCAAAGGTTTTAATTTAAAA
TCAT
>hg38_circ_0033082_junction_seq
TGTAGATGATGAAAGCCAATGTGCCTTTTCCTCCCTCTGTAGTTCAGACTTCATGTCAAGAAGGAGACCAAAGA
TGCCTCTACAATCCCTGCCTTGATTCATGTGGTGGTAGCTCTCTCTGTGACCCGAACAACAGTCTGAATAACTG
TACCTGTCACAGCCAGGGTCTGGTGGAATGCAGAAATGGACAATGTATCCCCAGCACGTTTCAATGTGATGGTG
ACGAGGACTGCAAGGATGGGAGTGATGAGGAGAACTGCAGCGTCAGTAAGTGTGTCCCACCACCCCAGAACATT
CCTT
>hg38_circ_0033095_junction_seq
GACACCTCTCCTCTCAGTTGTCAGAAACATGGAAATGGAAAAGTTCCTCAAGTTTTTCACATATCTCCATCGCC
TCAGTTGCTATCAACATATCATGCTGTTTGGCTGTACCCTCGCCTTCCCTGAGTGCATCATTGATGGCGATGAC
AGGAACATTACAAAAGGTCTATTTTAAATCAAATGGGAGTGAACCTTTGGTCACTGATGGTGAAATCCAAGGGT
CCGATGTTATTCTTACAAATACAATTTATAACCAGAGCACTGTGGTGTCTACTGCACATCCCGACCAACACGTT
CCAG
>hg38_circ_0033988_junction_seq
CTCTAGAAGCATTGGAGCACTTAATAAGATAAACTTCAATACACGTTTTGTTATGAAGACTTTAATGACTATAT
GCCCAGGAACTGTACTCTTGGTTTTTAGTATCTCATTATGGATAATTGCCGCATGGACTGTCCGAGCTTGTGAA
AGTTGTTCATGGTGGACAATGGAGCAGATGACTGGAGAATAGCCATGACTTATGAGCGTATTTTCTTCATCTGC
TTGGAAATACTGGTGTGTGCTATTCATCCCATACCTGGGAATTATACATTCACATGGACGGCCCGGCTTGCCTT
CTCC
>hg38_circ_0034015_junction_seq
TTCTCTTAATCATACAGTGCCCTTTCAGATGTTCTAAAATGGCCAAAATAGCAGTGACTCCTATTTTGTAACTT
GGTGATTCTTAATACAGCTTCCATTTAATGGTCTAAGATTAGGAGACCACAAGTCCCTGGAGCAACAGGAGCAA
ATTCCCAAGGCACGTAGATAGTACCTATACATCTCATCAGGAACATGGATGGTTCTCTGCTATCAGCTGAAGTG

CTGTGACCACAAAACTGAAAATTCCCATTCTGTGTCCTTGCCAATAAAGAATGTCCATCAGAGCACGCCCAGTA
TCCT
>hg38_circ_0034294_junction_seq
AAGAAGTTGATCTTCAGACAGCCCTTACAGGGTATCAAACAAAACAGCGAAAGCTTCTAGAACCAGTTGATCAT
GGAAAAATTGAGTATGAGCCATTTAGGAAAAACTTCTATGTTGAAGTTCCAGAACTAGCAAAAATGTCTCAAGA
AGAAGTCTGGGCCAACGGTCACAAAAGTTGTCACTGTTGTGACAACCAAAAAAGCAGTTGTGGATTCTGATAAG
AAGAAAGGTGAGCTGATGGAGAATGACCAGGATGCCATGGAGGTGATTTTTCTTAATTTTGACTTTGTTTATGA
TACT
>hg38_circ_0034628_junction_seq
CGTTTCTTAACACAGGGAGATGCAACAATAAGAAAGATGCTGAGCTTCTGGTGGCCTTTGGCTCTAATTCTGGC
CACACAGAGAATCAGTCGGCCTATTGTCAACCTCTTTGTTTCCCGGGACCTTGGTGGCAGTTCTGCAGCCACAG
AGGTTGTTTTTGTAGCCATTTTGCTTCACAGTCACCTGGAATGCCGGGAGCCCCTGCTCATCCCGATCCTCTCC
TTGTACATGGGCGCACTTGTGCGCTGCACCACCCTGTGCCTGGGCTACTACAAGAACATTCACGACATCATCCC
TGAC
>hg38_circ_0034663_junction_seq
GTCTAGTGCGTTAAACTACCATGGTTCTTCTCAGTAGCACTGAGTATATCTTTTTTATTTTATTCTTTTCCACA
GGCTGCAACAAAGAAACAAAAATATGAAAAGATTAGTGAAAAGAAGATGTCCACGCCTGTTGAAGTTTTATGTA
AGTTATTCCTTATTGATTTTGGTTTGGCCAAAAAGTACAGAGACAACAGGACAAGGCAACACATACCATACAGA
GAAGATAAAAACCTCACTGGCACTGCCCGATATGCTAGCATCAATGCACATCTTGGTATTGAGCAGAGGTAAGT
TCGA
>hg38_circ_0034669_junction_seq
CCCTGCTCTTTCCTCAGGATAGAGACCGAATTGCTACCCAGATCTGGAGTAGGAGACCAGAGACGAGACCTGAG
AGACTTTCACAAATGGTGGGATAAAGCCATTGATTAACTGTCCCCAAAGGCCTGTGATGACTGTTTGGTGTTTT
AGGCTTGGGGCAGGGGGCCCTTGGAGATACAGGTACACCTTGCCATTTCCAGAAGAGAGGAAGCTGAGGTCCAG
CTGCTTGGCTGGAGGAAGCCCAGGCCCCTGACTCCCTGGCAAGTTCATTTCCAGTGTCAGGTCTTGCGAGGTGC
GGTG
>hg38_circ_0034774_junction_seq
ATGAAAATTAACCTCTGGGTCCGGGAGTGCTGAAGGTGCCAGAACAGGAAGGGAGGTGGTGAGCCTGAAGATCT
CACAAGCTTCCCTCCTGGTGTGTGTTTCCAGACCGTCAAGGGTCCTGGCTGCTCATGGTCTATCTCAGTTGCCC
AGATCTCTTGGACTCAGTGCCTTTAGACTTACAGGTTCCTAAACACAGGTGGGAGGTTGACAAGGTAAGGGAGG
AAAGCGGTGCTGTTTCTTACCTTCATGGAAACACGTAGGAAGAGGTTTTCTACCTTCTTGCTTATGTGTAACCA
CATT
>hg38_circ_0035378_junction_seq
ATAGTTATGCCAATTCTAAATAAAAATTGCTCCCTAACTACTCTTTTTGATGTTTTCTTTTTAAAACAGGGAAA
TGCACATAAAATCATGGAGAAATGTACATTACCATTGACTGGAAAGCAATGTGTCAACCGCATTATTACTGAAA
AGGTTGAAGAAATTGTGGATATTGGAGCATTTGCTCCAGAAGACATCCATATTCCTCAGATTTATGTACATCGC
CTTATAAAGGGAGAAAAATATGAGAAAAGAATTGAGGTAATTGACTTAGCTGCTTTGTCAGTATTCATGGATCC
AGTT
>hg38_circ_0035648_junction_seq
CCTCTCCCCATTTTAGTCATATGGCCTTGAACCCACAGTGAATTGAAGAGAGAAAGAAATGGATATGTCTGACC
CCAATTTTTGGACTGTGCTCTCAAACTTTACTTTGCCTCATTTGAGGAGTGGGAACAGGCTTCGGCGAACACAA
AGATACCAAAAGGAAACTCGGCACTGGGCATGTTCGGATGTGAATGGTTTTGCTGATAAGCAACAGCCTCCGGA
ACCAAAGCCTCAAAATAGTCGTCTCCCCCGCCCCTCGCAGAAGCGGCGAGTGGTATCTGCCAAACAGATGACAA
AGTA
>hg38_circ_0035978_junction_seq
GGGTGAGCCAGTTATTTATAATTCTACCTTTCTCTGTTTCTAGGGAAACTGCAATCTGTTCTGAAATGACTGCT
GACCGAGATCCAGTCTATGATGAGAGTACTGATGAGGAAAGTGAAAACCAAACTGATCTCTCTGGGTTGGCTTC
AGTTCAGACTTGGTTCCTTCAACCTAGAGAAAGTTGAAAACCCAGCTGAAGTCATTAGAGAACTTATTTGTTAT
TGCTTGGACACCATTGCAGAAAATCAAGCCAAAAATGAGCACCTGCAGAAAGAAAATGAAAGGCTTCTGAGAGA
TTGG
>hg38_circ_0036343_junction_seq
CCGGGACCTACCCCAATCACACTGTTTGCGAAAAGACAATTACAGTACCAAAGGGGAAAAGACTGATTCTGAGG
TTGGGAGATTTGGATATCGAATCCCAGACCTGTGCTTCTGACTATCTTCTCTTCACCAGCTCTTCAGATCAATA
TGCCAGCCAGGTCCAGGCATTGGGGTTTAGTGGCCAGAGCTGGGATTATTTCGGCTCCTTATGCCCTGTTCTTC

TGGATCCGTTTAACTAAAGGTACATATTTACATAGATTGTTTCACATAATTTTGACCTTTCATAATAAGACATT
AAGT
>hg38_circ_0036543_junction_seq
ATCCGTAACCAAGAAATTTTGATTCCAACTCACTTCTTTATTGTGCTAACAAGCTGTAAAGATACATCTCAGAC
GCCTTTGCACTGTGAAAACCTAGACACCTTAGCTTTCATTTTGCCTCACAGGACTGATAACAGCGAGAGCTGTG
TGTTATATGGCGCTACTTTCATGACACCCTACTGCGAAAGTATGCTGAAGAAAGAAATGGTGTCAATGTCGTCA
GTGGTCCTGTGTTTGACTTTGATTATGATGGACGTTGTGATTCCTTAGAGAATCTGAGGCAGTAAGAACATATT
TCAT
>hg38_circ_0036568_junction_seq
CGACCTCAGCATCCTTGCTAAATGCTGGATTAAGCCACACTGAATACCAGATAGTCACAGACCCTGGGGACGTC
CCAGCTTTTTTGAAATGGCTGTCCTTAGCCAGCTTGCTTGTTTATGTTGCTGCTTTTTCAATTGGTCTAGGACC
AAGCTGCGGCATGTTTACCTTCCTGTCATCTGTCACTGCTGCTGTCAGTGGCCTCCTGGTGGGTTATGAACTTG
GGATCATCTCTGGGGCTCTTCTTCAGATCAAAACCTTATTAGCCCTGAGCTGCCATGAGCAGGAAATGGTTGTG
AGCT
>hg38_circ_0037547_junction_seq
AGGCTGGGGTGGTGTCCACAGGCCTGATTCAGAATGGAGACTGGACCTTCCAGACCCTGGTGATGCTGGAAACA
GTTCCTCGAAGTGGAGAGGTTTACACCTGCCAAGTGGAGCACCCAAGCGTGACGAGCCCTCTCACAGTGGAATG
GAGAGCACGGTCTGAATCTGCACAGAGCAAGATGCTGAGTGGAGTCGGGGGCTTTGTGCTGGGCCTGCTCTTCC
TTGGGGCCGGGCTGTTCATCTACTTCAGGAATCAGAAAGGTGAGGAGCCTTTGGGAGCTGGCTCTCTCCATAGG
CTTT
>hg38_circ_0037549_junction_seq
AGGCTGCGGTGGTCTCCATAGGCCTGATCCAGAATGGAGATTGGACCTTCCAGACCCTGGTGATGCTGGAAACA
GTTCCTCGGAGTGGAGAGGTTTACACCTGCCAAGTGGAGCATCCAAGCGTGACGAGCCCTCTCACAGTGGAATG
GAGAGCACGGTCTGAATCTGCACAGAGCAAGATGCTGAGTGGAGTCGGGGGCTTTGTGCTGGGCCTGCTCTTCC
TTGGGGCCGGGCTGTTCATCTACTTCAGGAATCAGAAAGGTGAGGAGCCTTTGGGAGCTGGCTCTCTCCATAGG
CTTT
>hg38_circ_0038387_junction_seq
GGAGGAAACCGGGAAGTTCCATGTGAAGGTTACTGTGATACCCTAGGTGAGCAGCGTTGGAGAATTGGATGAGA
ACACAAGAGGAGAGACAGACAGTAATAGAGTCAAGAAATATAAAGCAGAATAATAAAGCTTGCTGATGGATTGG
AGGGTTTTGTGATTGACTGGGTTCAGCTGGATTCCTGGAAGAGGTCTCTCATTCAATTGCAGTCAAATTGTAGC
TGAGACTACAATATGAAGACTCAGTTGGATTCGGATAGCTTATTCACATGGTTGATTGTTGATATTGGCTATTG
ACTA
>hg38_circ_0039368_junction_seq
GGAGCAGCCGCTCGCAGTCTTCCACCGAGGATGACTCAGTGGACTCTCTGCTCTCTGACAGATATGTGGTGGTG
TCCGGGACCCCGGAGAAGATTTTGGAGCACCTTTTGAATGACTTGCACCTGGAAGAAGTCCAGGACAAAGAAAC
AGGCAGGGGTGATGTGCAAGCTCCAGGAAAGAGATGAAATCGGACGAATTGAACTAGTCCAGAAGCTGGCAAAA
GAAAACTATCAGTTTTTGCAGACGGACAAAAAAGAACAGGAGAAGTCTGAACACGTAAGCCTTTTGCCTCTTGG
GGAT
>hg38_circ_0039497_junction_seq
GTGGCAGCTGCCCTGACAGAGCGCTCCTTGATGGGCATGGACTGGAAAGGATCCCAGGAATACAAGAAGGCAGA
AAAAAAAGTTTGGAAGATCTTTAAATCTGACAGTGAAGTGGCTGGTTACATCCGGCAAGCGGGTGACTTCCATC
AGATACTGGATAAACTACTAGATGGCGACTTAACAAGTGATCCTTCTTACTTCCAGAATGTTACAGGATGTAGT
AATTACTATAACTTTTTGCGGTGCACGGTAATGACATTTTAAAAACCATAATAATGTTTGCTTAAAACTTTTGG
CAAA
>hg38_circ_0039548_junction_seq
CAGACTTAAAGATGTTTATAACAACCCCCTCAGCTGCATACATATACTGTTTGTCATGTTTATTACTTTCAGGT
GACTTCAAAGATGGTAAAAATAAGACAGACAAGAAGGATCACTCTAACATCGGAAATGATTCAAAGAAAACAGA
TGAGGAGAAGGCCAAGAAGGAAGCAGAGGAAAAGGCTCGCCTGGCCGCAGAGGAGCAGCAAAAGGAAATGGAAG
CCAAAAGCCAGGCTGAAGAAGGCGCATCTGGCAAAGCTGAGAAAAAGACGTCTGGAGAAACTAAGAATCAAGTC
AATG
>hg38_circ_0039552_junction_seq
AGGCTGAAGAAGGCGCATCTGGCAAAGCTGAGAAAAAGACGTCTGGAGAAACTAAGAATCAAGTCAATGGAACA
CGGGCAAACAAAAGTGACAACCCTCGTGGGAAAAACTCCAAAGCCGAGAAGTCATCAGGAGAACAGCAACAGAA
TGGTTTCATTGATTTCATCGTGGAACCCACCTTCACTGTGCTTACGGACATGACCGAGAAGATTGTGAGTCCAT

TAATCGATGAAACCTCTCAAACTGGTGGGACAGGACAGAGGCGTTCGAGGTCAGTGGGGAAGCTTGAGGGCTGA
GGGT
>hg38_circ_0039558_junction_seq
CCAAGCGATCAGGTGTCAAGACCTCTGGTTCAGAGGGAAGTGCCCCGATCAACAATTCTGTCATCTCCGTTGAC
TATAAGAGCTTTAAAGCTACTTGGACGGAAGTGGTGCACATCAATCGGGAGAGATGGAGGGCCAAGGTACCCAA
AGGGTGACAGAGAAGCAGAGCTGGGGCTGCCTTTTTCTCCTCTGTGTGACCGAAAGTCCACTATGGTTGCTCAG
TCACAAGTAGGTACGTGTTTGCAGCTCTTTGGATAGAAGCACCAAAGCAGGAAGCCTGTAGAAGTAAGCACAGA
TGAT
>hg38_circ_0039640_junction_seq
AGATATGGGAAGTTTCATGGATACGGACCAGAGGAAAACAGTTTCTCAAGGACGTGCAGCGATTCTAAACCTGC
TGCCCATCACCAGCTACCCCAGACCTCAAGTGACTTGGTTTAGAGAAGGGCACAAGATTATTCCAAGCAACAGA
ATATGATGTTGCTCCATATTTTAAAACGGAGCCAGGCCTACCACAGATCCACCTGGAAGGGAACCGCCTTGTTC
TCACCTGCCTTGCCGAAGGGAGCTGGCCTTTGGAGTTCAAGTGGATGCGCGATGACAGTGAGCTCACCACCTAC
AGCA
>hg38_circ_0039926_junction_seq
TTGGTAATCAGATTATCCTAGCAAGAGTCATGTTATTTTTTTCTAATAAAACAGGTCTTGCTATCTTTAAGCCA
GACTTGATTACCTATCTGGAGCAAAGAAAAGAGCCTTGGAATGCGAGGAGACAGAAGACAGTAGCCAAACACCC
AGCGAGTGCTAACATTCAGGGATGTGGCTGTAGAATTCTCCCCAGAAGAGTGGGAATGCCTGGACTCTGCTCAG
CAGCGTTTGTATAGGGATGTGATGTTAGAGAACTACGGAAACCTGTTCTCCTTGGGTGAGAATAACTTCAATAT
ACAA
>hg38_circ_0040200_junction_seq
GCTCCTTTTAAACCCAGTGAACCTGGAGCCAATATGAGGCACATAAGGAAACCTGTTATAAAGCCAGTTGAAAT
CTGAATATGTGAACAAATCCAGGCCTCTCAAGGAAAAGACTTCAACCAGGCTTCCTTGTACCCACAGGTGAAAA
ATATGAATGGCTATCAAAGAAGGATCAAAAATGCAACTGAGAAAATGATGGCTCTTGTTGCTGAGCTGTCCATG
AAACAAGCCCTAACCATTGAACTCCAAAAGGAAGTCAGGGAGAAAGAAGACTTCATCTTCACTTGCAATTCCAG
GATA
>hg38_circ_0041346_junction_seq
ATGGTGCGCATCCCCGTGGGATCAAGAGAGACCTTCTACAGGATCTCCAAGACGGATGGCGTTTGTGAATGGAA
ATTTTTCTCTTCCATGCCCCGTGGATCTGCCAGGATTACAGTGTCCAAAGACCAATCCTCCTGTACCACAGCCC
GGGGTCTTTCTTCCCTGATTTCAAGCCCTCTGAAACAGTTTTTAAAATAGTATTTTGGCTCGGATATCTAAACA
GCTGCATCAACCCCATCATATACCCATGCTCCAGCCAAGAGTTCAAAAAGGCCTTTCAGAATGTCTTGAGAATC
CAGT
>hg38_circ_0041573_junction_seq
TGCTGCTGTCTGCAGGTGGAGCTGAGAGGGAGTGGCCTACAGCCGGACCTGATAGAGGGCAGGAAGGGGGCGCA
GATAGTGAAGCGGGCCAGCCTGAAAAGGGGGAAACAGTGACCCCGAGCCGCTCTCCTTGGAGTAGCCTCTCGGG
AGGGGAATGAGTTTCAGAATATTCCAGGGGAGCAGGTGACAGAGGAGCAATTCACGGATGAGCAGGGCAACATT
GTCACCAAGAAGGTGGGTGCAGAGTGTTCCCCTCTGTGCTGGGGAGAGGCAGGTGGGCTAGAAGCTAAAAGGTG
GTAG
>hg38_circ_0041674_junction_seq
GTGTGAGCAGTTATTGGGGTCACCAGCCACCAGCTCCTCCCAAAGTGTGGCTCCCAGGCCTGGAGCTGGCCTGA
AAGTTCTCTTCACCAAGGAGACTGCAGGCTACCTCAGGGGCCGTCCCCAGGACACTGTCCGGATCTTCCCTCCC
TGCCAAGTTCTATAGAAATTTTAGAGTATTCATCAGATAGTGAAAAAGAAGATGATTTGGAAAATGTCCTACTC
ATTGATTCAGAATCCCCTCACAAATACCACGTGCAGTTTGCATCGGATGCAAGACAGATTATGGAGAGACTGAT
AGAT
>hg38_circ_0041718_junction_seq
TCCCTGTGGAAGGCCAGCATACAGTTCTCTCCTCTGGCACTTTGAGAATTGACCGTGCAGCACAGCACGATCAA
GGCCAATATGAATGTCAAGCAGTCAGTTCGTTGGGGGTGAAAAAGGTGTCTGTGCAGCTGACTGTAAAACCCAA
AGCCACTCATTGGATTTGGAAGATGATACTCGACTTAATGTGTTTGATGATGGCACACTCATGATCCGAAACAC
CAGAGAGTCAGACCAAGGTGTCTATCAGTGCATGGCCAGAAATTCCGCTGGGGAAGCCAAGACACAGAGTGCCA
TGCT
>hg38_circ_0041754_junction_seq
TTGATGAAATAACTGGGAAGGTTCTTATTCTTACTGTTTTTATTCAGTTCCTTTATTTATAGGCACAGCTGCTT
AAAGACTGTTACCGGAAAGGAATCCTGATCCAGACGCCAAGAGAGGGTTCTTGGACTCATGCAAGAAAGACTTC
AGACATCTGCAATGGCAGTTCACATGGCTCAGATAGCTCACTTCTGCAGAGAGCTGAATTTAAGTGGGACAGGG

TAGCAAGAATAATTACTTTGACAATCATTTTGCCACCTGATGACTGTGCTGCCTGTCCTGAGCTGAGGTGTGTT
GTGA
>hg38_circ_0041994_junction_seq
CACTCTCTTTCATGTAACCTGAAGTATACATGTTCTTCACCTGTCATATTCTTATTTTAGGATCCACCCACTTC
AGTTTCCCTTGGACTGCGAATGGAAGAAATGATTTTCAACTTGGCAGACACACATTTATTTTTTAATGACTTAG
AAAGTACAGCACAATCCACAGCCCATCAACACCCATTAAAGATTCAGATTCTGATCGATTGCGTCGAGGTTCAG
ATGGGAAATCACGTGGACGGGGCCGAAGAAACAATAATCCTTCACCTCCCCCAGATTCTGATCTTGAGGTACGT
CATA
>hg38_circ_0042157_junction_seq
TCACCAAGAATATTATTTTTGGTATTTGGAATTGAATTTGCCAGGAATGCAAGCACTGCTACCTAACTAAAGAC
CTTGGCTTTGGAAACATCTTACCCCTTGGCAGCTCTCCAGAGTTTAGACTTGGCAGCATCAAAGACACAACGCA
AGTCACCTCGTGAGACAGGTATTAGAACTCCGGAGTGGCATCAAAATGAGGGAAAGGCCTCAGGGCCAGCGCCT
TTGCGTCCTGGACCGGCGTACAGACGGGAGAAAGGCCGACCAATCCGAGAGCAGGCTGAGGCCTGGTCCCGCCC
CTGG
>hg38_circ_0042430_junction_seq
GTTCTACTTTAAAGATAAGTAATTCTCTTTTTCGTTTTTGTGCAGATGATCATCTGGTTGGAGAAGATGTTAGA
TAAAATAATTAGCATTTTCATCATATTTTTGTTAGTGATAGGAACTCTTCTTTTAGCCCTACTCCTGACTGCAA
AGCAGTGGACAGGGGAGAATCCGCTCCAACGTTGTCCACCTCCCCTTCACCCTCCTCCCCTTCACCCACTTCCC
CTTCACCTACTCTGGGCAGACGAAGGCCTGAAATAGGAACGTTTCTTAGAAAGAAGAAAACTAGTGACATCTAC
TTTG
>hg38_circ_0042445_junction_seq
CTGTCTTTTCTGGTGAACTTTATCATCAATGCAGTGCCGTCTCTAACCAGGGGTGTTTGTCTGTCTCCCTAGAG
CCCCTCTGACCGAGGAGGTGCCTTTACCCTGGAGCCGGGTGATCTTCTGATGGATTTCACAGAAGCCACTCCTC
TGAAGTCCTATTCCTATTCGTGTGGAAACTGCCCAGCCAGCTGTGGAAAAGCCGGAAATCAAGCCTCCCCGAGT
GAGGAAGTTAACAAGACAGTATAGTTTGTGAGTTCTGTACTGCAACTTTTTCAATTAAAACAACTATGATGACT
AATT
>hg38_circ_0042667_junction_seq
CTCTCTTCCTAACAGTCTACTCTGGATGATGCCACTGATGCCTGGGGAATAAAGGTGGAGCGTGTGGAAATTAA
GGATGTGAAACTACCTGTGCAGCTCCAGAGAGCTATGGCTGCAGAAGCAGAAGCGTCCCGCGAGGCCCGCGCCA
AGGTTTGTTTTTTATTCTGCCATGCACTGACAGCTTCATCAAAGTGGACATGAGAACTATTTCATTTGATATTC
CTCCTCAGGAGGTAAGGTTTTCTTTGAATAACTGAGGTATCATATGTGAAAGTGCTTTACAATGAGGGACAGAA
TAGT
>hg38_circ_0043481_junction_seq
TTACATTTTTTTTAAATGTTGACTGTTTCAATCTCAGAGTTGGATGCACTAGGTGATGAGCTTCTGGCTGATGA
AGACAGTTCTTATTTGGATGAGGCAGCATCTGCACCTGCAATTCCAGAAGGTGTTCCCACTGATACAAAAAACA
AGGTATGAGCAGCAGCGGGACAATCTTGCCCAACAGTCATTCAACATGGAACAAGCCAATTATACCATCCAGTC
TTTGAAGGACACCAAGACCACGGTACTCCCAAAACACCTTTTCCTGTTATTTCATGTATTTATTCTTATTCTCT
TTTC
>hg38_circ_0043582_junction_seq
AGATCATGCTTTCCCTTTCTCCTCCCACACCTGCACACCCAGGAAAGGCATGCAGCAAGAAGCTCAAAATCTAC
AAGCCAGGAAGAGAGCCCTCACCAGAAACGGAAACAGCTGGCACTTTGATCTTGGACTTCCCAGGCCTCCAGAA
CGGTCAAATCCCGAGGTGTGGTCAGCCCACAGAAGAATTTTGGAGGGCCCAGCACTTTCCAGGGCCTGTCACTT
GGACATTAAAACCTGCGATCATCCAAGAGAAACAGAGGTTGGCCCCCCAAGTATCCCTGTTACAGTCCAGGAAG
GTGA
>hg38_circ_0043995_junction_seq
GTTATAACTTTTGAGAAATTCTACTGTATTTATTACTTTTATTTATTTATTTATTTTTTAGATTGTTCCCTAAG
ATTATATGGGTCATCCTGTAGCAGATTGGGTTTCAAAAATTCGGATGTAAACATTGACATCCAGTTTCCAGCCA
TTAAAACAAGAAGCAGAGGTCCAGACCTAGGAAGCCACGGAAGACTAGAAATGAGGAAAATGAGCAGGATGGAG
ACTTGGAAGGCCCTGTGATCGATGAGTCTGTACTTTCAACGAAGGAGCTGCTAGGCTTACAGCAGGCTGAGGAG
AGAC

Table S3. Construct sequences

CP28-circItga9
tctcgagacacttagccgtgttctttgcactttctgcatgtcccccgtctggcctggctgtccccagtggcttccccagtgtgacatggtgcatctctgccttacagagcaaggcctgtcat
tacggtggatgtctccatcttcctcccgggctccatcaacatcacagcgcctcagtgtcacgacggacagcagcctgtgaactgcctgaacgtcaccacctgcttcagcttccatggca
aacacgttccaggagagattggcctgaattatgttctgatggctgacgtggccaaaaaggagaagggccagatgcccagggtctactttgtgctgctgggagagaccatgggtcagg
tcacagagaagctgcagctgacttacatggaggagacgtgtcgtcactatgtggcccatgtgaaggtgagtggcccgctacctcttctggtggccgcctccctccttcctggcctccc
ggagctgcgccctttctcactggttctctcttctgccgttttccgta**ggatcc**t

CP90.hucircItga9-mut-TPM3
Tctcgagacacttagccgtgttctttgcactttctgcatgtcccccgtctggcctggctgtccccagtggcttccccagtgtgacatggtgcatctctgccttacagagcaaggcctgtca
ttaTAAtggatgtctccaGGttcctcccgggctccatcaacatcacagcgcctcagtgtcacgacggacagcagcctgtgaactgcctgaacgtcaccacctgcttcagcttccat
ggcaaacacgttccaggagagattgAATtgaattatgttctgatggctgacgtggccaaaaaggagaagggccagatgcccagggtctactttgtgctgctgggagagaccatgg
gtcaggtcacagagaagctgcagctgacttacatggaggagacgtgtcgtcactatgtggcccatgtgaaggtgagtggcccgctacctcttctggtggccgcctccctccttcctgg
cctcccggagctgcgccctttctcactggttctctcttctgccgttttccgta**ggatcc**t

Sequence of mutant circItga9 (red nucleotides were mutated)
agcaaggccugucauuacgguggaugucuccaucuuccucccggg cuccaucaacaucacagcgccucagugucacgacggacagcagccugugaacugccugaac
gucaccaccugcuucagcuuccauggcaaacacguuccaggagagauuggccugaauuauguucugauggcugacguggccaaaaaggagaagggccagaugccca
gggucuacuuugugcugcugggagagaccaugggucaggucacagagaagcugcagcugacuuacauggaggagacgugucgucacuauguggcccaugugaag

CP120.musTPM3-His-pcDNA3.1
gccgccaccatgatggaggccatcaagaaaaagatgcagatgctgaagttagacaaagagaatgttctggaccgagctgagcaagctgaagctgagcagaaacaggcagaagaa
agaagcaagcagctagaggatgaactagcaaccatgcagaagaagctgaaagggacagaggatgagctggacaagtattcggaagctttaaaggatgctcaggagaagctggag
ctagcagagaagaaggcagccgacgctgaagctgaggtggcctccttgaaccgcaggatccagctggttgaagaggagctggaccgtgcgcaggagcgccttgccactgctttg
cagaagctggaggaagcagagaaggctgctgatgagagtgagagaggtatgaaggtgattgaaaatcgggctctaaaagatgaagaaaagatggaactccaggaaatccagcta
aaggaagcaaagcacattgcagaagaggccgataggaagtatgaagaggtggctcgtaagttggtgattattgaaggagacttggaacgcacggaggaacgtgctgagctggca
gagtctaagtgttctgagctggaggaagagctgaagaatgtcaccaacaacctcaagtctcttgaggctcaggcggagaagtactctcaaaaagaagacaagtatgaagaagaaata
aagattcttactgataaactcaaggaggcagagacccgtgctgagtttgctgaaagatcggtagccaagctggagaagaccattgatgacctggaagacgagctctatgcccagaaa
ctgaagtacaaggccattagcgacgagctggaccacgccctcaatgacatgacctctatacaccatcaccatcaccatcactaa

circItga9 probe for in situ hybridization
Cccgggaaaccc actatgtggcccatgtgaagagcaagg**cctgtcattacgg**actatgtggcccatgtgaagagcaagg**cctgtcattacgg**
actatgtggcccatgtgaagagcaagg**cctgtcattacgg**actatgtggcccatgtgaagagcaagg**cctgtcattacgg** tttcccgggccc

Table S4. Primer sequences

32.78.HuMu.cir.0064840-F	5’ tccaacctccaggccaaaggca
32.79.HuMu.cir.0064840-R	5’ cttaaacacagccccaggagactt (119 bp) circ-Itga9
33.1.humu.hg38cir.30598-F	5’ ggacagca**gcctgtgaactgcctg**
33.2.humu.hg38cir.30598-R	5’ gccatttccatccatatcaatgcc (160 bp) circ-Itga9
33.3.humu.hg38cir.30600-F	5’ gagaagctgcagctgacttacatgga
33.4.humu.hg38cir.30600-R	5’ ttgccatggaagctgaagcaggtggtg (108 bp) circ-Itga9
33.28.Hu.cir.Itga9-actin-intron-F	5’ tgcatctctgccttacag agcaaggcctgtcattacggtgga
33.29.Hu.cir.Itga9-actin-intron-R	5’ aggtagcgggccactcac cttcacatgggccacatagtgacg
33.30.Hu.cir.Itga9-no-intron-F	5’ tgcatctctg ctcgag agcaaggcctgtcattacggtgga
33.49.cir-Itga9-exon-R	5’ catccaccgtaatgacaggccttg
33.50.cir-Itga9-exon-F	5’ catggcaaacacgttccaggagag (205 bp)
34.5.circItga9-Northern-F	5’ tcgtcactatgtggccca
34.6.circItga9-Northern-R	5’ atccaccgtaatgacagg (large scale)
34.25.mu.circItga9a-R.	5’ cagctgttcctcgagggctc
34.26.mu.circItga9a-F.	5’ gtctacatctatcatggtga (72 bp)
34.27.mu.circItga9b-R	5’ gacttgctcattagatccaac
34.28.mu.circItga9b-F	5’ gccgatctgcttttcagtctatta (111 bp)
34.29.mu.linear-Itga9-R	5’ gtgcacctcagtaaagctgcag (combine with 34.28, 157 bp)
34.42.mu.circItga9c-R.	5’ aggaagatggagacatccacc
34.43.mu.circItga9c-F.	5’ gtgtcatcactacgtggccca (67 bp)
34.54.hu-circItga9-probe-F	5’ **tgcagctaac**actatgtg
34.55.hu-circItga9-probe-R	5’ cattctggaaccgtaatg (large scale, 180 bp)
34.70.mu.circItga9c-F2.	5’ ctacgtggcccacgtcaagggc (to combine with 34.42.mu.circItga9c-R, 58 bp)
**HPLC purified blocking oligo**	
37.19.mus-cirItga9-Tpm-blocking	5’ tgttgatggagcctggcaggaagatggagacatccaccgt
37.11.cirItga9-Tpm-blocking	5’ gggaggaaCCtggagacatccaTTAtaatg

Table S5. siRNA sequences

1.mus-si-TPM3
5' ccugaagcugaccuugaaauu
3' ccggacuucgacuggaacuuu

2.mus-si-TPM4-2
5' cccaggccaaagaagagaauu
3' ccggguccgguuucuucucuu

1. Mus-si-Itga9
5' gcccacgucaagggccagauu
3' ggcgggugcaguucccggucu

2. si-hg38_circ_0028615
5' Cccucuugcucuaug**cucauu**
3' ccgggagaacgagauacgagu

3. si-hg38_circ_0007593
5' Caauaacuguuuaagauuauu
3' ccGuuauugacaaauucuaau

4. si-hg38_circ_0025066
5' Gaucugagcag**cgacugcuuu**
3' ggCuagacucgucgcugacga

5. si-hg38_circ_0035378
5' cugaaaagguugaagaaauuu
3' ccgacuuuuccaacuucuuua

6. si-hg38_circ_0017433
5' gaugag**gccaucucggaaauu**
3' ggcuacuccgguagagccuuu

7. si-hg38_circ_0005451
5' caagcacagauugaguuuuuu
3' ccguucgugucuaacucaaaa

8. si-hg38_circ_0039574
5' ccaaaggauaucuguguuuuu
3' ccgguuuccuauagacacaaa

9. si-hg38_circ_0008333
5' gaggaggagggcaag**gcaauu**
3' ggcuccuccucccguuccguu

10. si-hg38_circ_0006743
5' gaucuuugcaa**guucauaauu**
3' ggcuagaaacguucaaguauu

11. si-hg38_circ_0042258
5' ccugcagcc**agauagacuauu**
3' ccggacgucggucuaucugau

12. si-hg38_circ_0034177
5' gugacuggucuaag**gagaauu**
3' ggcacugaccagauuccucuu

13. si-hg38_circ_0033095
5' ggcgaugacag**gaacauuauu**
3' ggccgcuacuguccuuguaau

14. si-hg38_circ_0015928
5' gauggag**gcacaggagaaauu**
3' ggcuaccuccguguccucuuu

Table S6: The circITGa9-TPM3 distance table reporting a list of atoms "in contact" (within the distance cutoff) with relative distances less than 3.5Å. The hydrophobic/hydrophilic character of the corresponding residues is also specified. Hydrophilic (phil) and hydrophobic (phob) interactions with calculated distances between interacting residues. AA, type of amino acid; N, type of nucleotide.

N (residue number)	Atom	Chain	AA (residue number)	Atom	Chain	Distance	Type
C 18	O3'	A	LYS 141	NZ	B	3.26	phil phil
C 18	C5'	A	ARG 134	NH1	B	2.14	phil phil
C 18	O2	A	ASN 133	O	B	3.27	phil phil
C 18	O2'	A	ASP 138	OD2	B	2.27	phil phil
C 18	O2	A	LYS 137	CG	B	2.46	phil phil
G 19	O4'	A	LYS 137	CB	B	1.78	phil phil
G 19	OP1	A	ASP 138	OD1	B	2.28	phil phil
G 19	O5'	A	LYS 141	NZ	B	0.93	phil phil
G 20	P	A	LYS 141	CD	B	2.13	phil phil
U 33	C4'	A	ALA 156	CB	B	3.2	phil phob
C 34	OP1	A	ASP 169	OD2	B	2.77	phil phil
G 45	OP1	A	GLN 148	NE2	B	2.96	phil phil
A 54	O4'	A	LYS 137	NZ	B	3.47	phil phil
A 54	C2	A	ARG 134	CG	B	3.02	phil phil
A 54	N3	A	ASN 133	CB	B	3.06	phil phil
C 55	O4'	A	ASN 133	CG	B	0.95	phil phil
C 55	O2'	A	LYS 129	C	B	2.41	phil phil
C 55	O2'	A	VAL 130	N	B	2.44	phil phob
G 156	OP2	A	ARG 91	NE	B	2.74	phil phil
G 156	C5'	A	ASN 90	OD1	B	3.08	phil phil
G 156	O3'	A	GLN 94	NE2	B	1.54	phil phil
C 157	OP2	A	ARG 91	CG	B	3	phil phil
C 157	OP1	A	GLN 94	CB	B	1.92	phil phil
C 158	OP1	A	GLU 98	CG	B	3.29	phil phil
C 158	OP1	A	LEU 95	CD2	B	2.42	phil phob
G 196	OP1	A	ARG 92	NE	B	2.49	phil phil
U 292	O2	A	GLU 123	CB	B	2.32	phil phil

Table S7: CircITGa9-TPM3 interaction overview

Number of interacting residues circITGa9 13
Number of interacting residues TPM3 17
Number of hydrophilic-hydrophobic interaction 03
Number of hydrophilic-hydrophilic interaction 24
Number of hydrophobic-hydrophobic interaction 0

Table S8: The ASA (Accessible Surface Area) table of circITGa9-TPM3 complex.

Buried area upon the complex formation (A^2) 2445.0
Buried area upon the complex formation (%) 2.94
Interface area (A^2) 1222.5
Interface area circ-ITGA9 (%) 2.17
Interface area TPM3 (%) 4.54
POLAR Buried area upon the complex formation (A^2) 1104.9
POLAR Interface (%) 45.19
POLAR Interface area (A^2) 552.45
NON POLAR Buried area upon the complex formation (A^2) 1340.1
NON POLAR Interface (%) 54.81
NON POLAR Interface area (A^2) 670.05
Residues at the interface total (n) 61
Residues at the interface circ-ITGA9 (n) 29
Residues at the interface TPM3 (n) 32

Table S9a. Summarizing demographic information of human participants

Diseases (N)	Males (N)	Females (N)	Mean Ages (SD)	Race (N)	Genetic ancestry
Normal (25)	21	4	36.4 (15.2)	Asian (25)	Han Chinese (25)
Hypertrophic diseases (73)	48	25	32.2 (18.8)	Asian (73)	Han Chinese (73)

Table S9b. Summarizing demographic information of human participants for sequencing

Diseases (N)	Males (N)	Females (N)	Mean Ages (SD)	Race (N)	Genetic ancestry
Normal (2)	1	1	23.5 (10.6)	Asian (2)	Han Chinese (2)
Hypertrophic diseases (8)	5	3	29.1 (17.7)	Asian (8)	Han Chinese (8)

Table S10. Patient information

**Normal, heart donation** (n=25)	SAMPLE ID No	AGE	SEX	PCR (n=25)
Normal	40L	48	F	real-time PCR
Normal	44L	51	M	real-time PCR
hypertension, brain death	51	28	M	real-time PCR
premature infante	65	1	M	real-time PCR
brain trauma	61	46	M	real-time PCR
brain hemorrhage	62	37	M	real-time PCR
car accident	68	47	M	real-time PCR
brain hemorrhage	69	32	M	real-time PCR
car accidnet	70	49	M	real-time PCR
car accident	71	57	M	real-time PCR
Brain death	74	1.5	M	real-time PCR
Brain tumour	77	23	M	real-time PCR
brain hemorrhage	79	42	M	real-time PCR
car accident	94	47	F	real-time PCR
car accident	95	24	M	real-time PCR
brain hemorrhage	104	37	F	real-time PCR
car accident	107	61	M	real-time PCR
trauma	119	19	M	real-time PCR

trauma	132	34	M	real-time PCR
brain diseases	137	35	M	real-time PCR
brain hemorrhage	139	23	M	real-time PCR
brain hemorrhage	145	50	M	real-time PCR
brain hemorrhage	146	40	M	real-time PCR
brain hemorrhage	147	55	M	real-time PCR
brain hemorrhage	153	22	F	real-time PCR
**Hypertrophic-related diseased heart** (n=73)	SAMPLE ID No	AGE	SEX	PCR (n=73)
	32	65	F	real-time PCR
	34	13	M	real-time PCR
	89	24	F	real-time PCR
	90	9	F	real-time PCR
	125	19	M	real-time PCR
	134	56	M	real-time PCR
	141	10	M	real-time PCR
	142	29	F	real-time PCR
	2	61	F	real-time PCR
	28	64	F	real-time PCR
	31	68	F	real-time PCR
	4	71	M	real-time PCR
	7	53	F	real-time PCR
	27	55	F	real-time PCR
	33	65	F	real-time PCR
	123	64	M	real-time PCR
	129	65	M	real-time PCR
	26	30	M	real-time PCR
	21	65	M	real-time PCR
	1	6 M	M	real-time PCR
	3	11 M	F	real-time PCR
	5	7 M	F	real-time PCR
	8	11 M	M	real-time PCR
	9	1	F	real-time PCR
	11	2	F	real-time PCR

	HF9	66	M	real-time PCR
	HF10	12	M	real-time PCR
	11L	50	M	real-time PCR
	12L	54	M	real-time PCR
	13L	64	M	real-time PCR
	14L	37	M	real-time PCR
	15L	52	M	real-time PCR
	16HF	41	M	real-time PCR
	17 HF	29	F	real-time PCR
	18HF	51	F	real-time PCR
	19HF	45	M	real-time PCR
	20HF	52	F	real-time PCR
	HF21	55	M	real-time PCR
	HF22	59	M	real-time PCR
	HF23	60	M	real-time PCR
	HF24	61	F	real-time PCR
	HF25	40	M	real-time PCR
	HF26	23	M	real-time PCR
	HF27	49	M	real-time PCR
	HF28	11	F	real-time PCR
	HF29	64	M	real-time PCR
	HF30	51	M	real-time PCR
	HF31	31	M	real-time PCR
	HF32	68	M	real-time PCR
	HF33	28	M	real-time PCR
	HF34	38	F	real-time PCR
	HF35	53	M	real-time PCR
	HF2	23	M	real-time PCR
	HF3	55	M	real-time PCR
	HF4	44	M	real-time PCR
	HF5	42	M	real-time PCR
	HF6	54	M	real-time PCR
	HF7	55	M	real-time PCR
	HF8	54	M	real-time PCR

	72	23	F	real-time PCR
	73	9 M	M	real-time PCR
	75	1	F	real-time PCR
	76	6 M	M	real-time PCR
	78	7 M	M	real-time PCR
	80	6M	F	real-time PCR
	81	2	M	real-time PCR
	82	1 M	M	real-time PCR
	83	8 M	F	real-time PCR
	84	2	F	real-time PCR
	86	7 M	M	real-time PCR
	87	6 M	F	real-time PCR
	88	7 M	M	real-time PCR
	91	6 M	F	real-time PCR
